# Bayesian factor analytic model: An approach in multiple environment trials

**DOI:** 10.1371/journal.pone.0220290

**Published:** 2019-08-22

**Authors:** Joel Jorge Nuvunga, Carlos Pereira da Silva, Luciano Antonio de Oliveira, Renato Ribeiro de Lima, Marcio Balestre

**Affiliations:** 1 Department of Statistics (DES), Federal University of Lavras, Lavras, Minas Gerais, Brazil; 2 Eduardo Mondlane University, Chibuto College of Business and Entrepreneurship, Chibuto, Maputo, Mozambique; 3 Faculty of Exact Sciences and Technology (FACET), Federal University of Grande Dourados, Grande Dourados, Mato Grosso do Sul, Brazil; Yunnan University of Finance and Economics, CHINA

## Abstract

One of the main challenges in plant breeding programs is the efficient quantification of the genotype-by-environment interaction (GEI). The presence of significant GEI may create difficulties for breeders in the selection and recommendation of superior genotypes for a wide environmental network. Among the diverse statistical procedures developed for this purpose, we highlight those based on mixed models and factor analysis that are called factor analytic (FA) models. However, some inferential issues are related to the factor analytic model, such as Heywood cases that make the model non-identifiable. Moreover, the representation of the loads and factors in the conventional biplot does not involve any measurement of uncertainty. In this work, we propose dealing with the FA model using the Bayesian framework with direct sampling of factor loadings via spectral decomposition; this guarantees identifiability in the estimation process and eliminates the need for the rotationality of factor loadings or imposition of any *ad hoc* constraints. We used simulated and real data to illustrate the method’s application in multi-environment trials (MET) and to compare it with traditional FA mixed models on controlled unbalancing. In general, the Bayesian FA model was robust under different simulated unbalanced levels, presenting the superior predictive ability of missing data when compared to competing models, such as those based on FA mixed models. In addition, for some scenarios, the classical FA mixed model failed in estimating the full FA model, illustrating the parametric problems of convergence in these models. Our results suggest that Bayesian factorial models might be successfully used in plant breeding for MET analysis.

## Introduction

The recommendation of new genotypes for commercial use requires confident and accurate estimations of genetic parameters such as marginal genotypic values, stability, adaptability, disease and environmental stress resistance. This information can be obtained by analyzing the genotypes across different environments; such an analysis is called multi-environment trials (MET). These trials are required to isolate the effect of the genotype-by-environment interaction (GEI), which means the differential genotypic responses on different environments. In general, the GEI hinders the breeder’s work on the selection and recommendation of the best genotypes for a wide class of environments. Thus, it is used to investigate efficient methods that identify stable genotypes (those that do not contribute to GEI) and positive effects of GEI for specific groups of environments aimed at regionalized recommendations.

Several statistical methods have been proposed. One is the additive main effect and multiplicative interaction (AMMI), and another is the genotype plus genotype x environment interaction biplot (GGEbiplot) [1–3]. These methods have been widely applied in plant breeding programs for the identification of mega-environments, which-won-where patterns, ideal genotypes, and specific adaptability, among others. Limitations inherent to these fixed-based parameters models (such as the lack of flexibility to treat unbalanced data and heterogeneity of variances) have motivated the development of more flexible methods, such as those based on mixed models. Piepho [4,5] and Smith et al. [6] proposed the MET analysis based on multivariate mixed models; it uses factor analysis (AF) structures that consider environments/genotypes and interaction as random effects.

In the literature, these models have been frequently referred to as factor analytic (FA) models [6–10]; they have shown great versatility for genotype selection since they combine the stability and adaptability studies into a single approach. The advantage of this approach is related to its ability to address missing data and the heterogeneity of residual and genotypic (co)variances. These models are also notable for allowing the inclusion of heteroscedastic residues of genetic values, which constitute an important aspect to be considered in the analyses [11,12]. It is known that the heterogeneity of variances among genotypes is affected by the heterogeneity of variances among environments and vice versa [13]. Furthermore, this model has been useful for summarizing the covariance pattern in multivariate data [14].

Despite the recognized advantages offered by FA models, the method also has limitations, such as the need to impose some constraints and, under some scenarios, the non-identifiability in parameter estimation. It is worth highlighting the difficulty to construct exact confidence intervals for the components of variance, since they are approximate and require assumptions of asymptotic normality. In addition, there is a great demand for computational resources and efficient algorithms to avoid the occurrence of solutions outside the parametric space (the so-called Heywood cases), among other aspects [15,16].

An interesting alternative to the frequentist or likelihood approaches is the use of Bayesian inference. Bayesian analysis allows greater flexibility for the construction of credible intervals for unknown parameters, since all inference processes are based on the posterior distribution. The flexibility of the Bayesian method for GEI analysis was partly illustrated by Crossa et al. [17], Oliveira et al. [18], Perez-Elizalde et al. [19] and Silva et al. [20], which incorporated credibility regions for the bilinear parameters in the AMMI model. It is known that inferences about genotypic and environmental scores in linear-bilinear models present great difficulties for frequentist methods or non-parametric approaches [17,18,21,22].

Using Bayesian inference in AMMI models, Perez-Elizald et al. [19] have shown how historical information can easily be incorporated into the model. Recently, Jarquin et al. [23] showed how this information could be included in the sites regression (SREG) model using the multilevel (hierarchical) Bayesian approach. Methodological innovations and applications of factorial analysis have been rapidly designed in recent years, partly due to access to computational tools for numeric integration in the Bayesian framework. In particular, it is possible to highlight the use of the Markov chain Monte Carlo (MCMC) in classical factor analysis [24].

The Bayesian analysis for FA model was presented by de Los Campos and Gianola [25]. These authors proposed prior distributions based on the assumptions of the classical factors analysis that avoids the imposition of constraints and reduces the computational requirements that have restricted the use of these models. However, this approach it not founded on the initial FA structure described in Smith et al. [6]. Instead, de Los Campos’s and Gianola’s [25] model accounts for the decomposition of the genetic variance, ignoring a more general structure for residual variances and performing a two-stage adjustment for the parameter of the model. In addition, only the balanced scenario was considered, since it does not consider situations where missing data are present, which is the great appeal of the FA models for MET data analysis.

Nevertheless, the advantages of the FA models in summarizing the MET network and finding data patterns from breeding programs are remarkable. Several successful applications can be found in Burgueño et al. [7,26], Kelly et al. [9], Meyer [16], Smith, Cullis and Thompson [6,27], Smith et al. [28], Stefanova and Buirchell [29] and Tyrisevä et al. [30].

This study proposes the Bayesian approach for the FA model applied in MET data using spectral decomposition to ensure the model identifiability. Additionally, we sought to evaluate the Bayesian FA predictive ability on unbalanced data with respect to the classic FA models through REML.

## Material and methods

### Material

#### Simulated data

We simulated a dataset with 20 genotypes (G1-G20) that were evaluated in five environments (E1-E5) using a randomized complete block design with two replicates. Five genotypes had interactions simulated from the Gaussian distribution with large variances (unstable genotypes) and positive marginal effects. Five more genotypes were marginally negative and contained large Gaussian variances (unstable). 10 genotypes were a standard Gaussian distribution, and the unstable genotypes had variance of 2 (three times the standard Gaussian distribution. Therefore, the stability and instability in this study were considered as a function of the size of the variability across the environments and the genotypes’ marginal effects as Gaussian realizations.

#### Experimental data

The experimental data were described by Melo et al. [31]. These data are related to grain yield traits measured in 50 maize single-cross hybrids (G1-G50). The experiment was conducted using incomplete block designs with 2 replicates, and each plot had 5-m rows with 70-cm spacing between rows. The grain yield was evaluated and adjusted for 13% moisture and converted into t∙ha^-1^. Come from.

These hybrids are originated from crossing among lineages of different backgrounds (tropical–Flint, Lancaster and Stiff Stalk Synthetic sources–see Melo et al. [31]). The crossing design was in a partial diallel system. These hybrids were evaluated during the agricultural years of 2013 and 2014 in 10 environments (E1-E10) representative of the Southeast and Southern regions of Brazil. Details on the environmental characteristics are given in the S1 Table.

### Method

#### Statistical model

The classical multivariate linear mixed model under the unstructured covariance matrix can be described as:

y=Xβ+Zu+ε (1)

where **y**_(*n*×1)_ is the vector of observations for *p* environments, *q* blocks and *m* genotypes. Vectors **β**_(*pq*×1)_, **u**_(*mp*×1)_ and **ε**_(*n*×1)_ are fixed, random and residual vector effects, respectively. The matrices **X**_(*n*×*pq*)_ and **Z**_(*n*×*mp*)_ denote information regarding **β** and **u**, respectively. For simplicity, it is assumed that **u** represents the additive genetic effects. Moreover, **ε**~*N*(**0**,**R**) and **u**~*N*(**0**,**Σ**⊗**I**_*m*_). In the FA framework, we can approximate the vector related to additive genetic effects by common and specific factors using **u** = (**Γ**_*p*×*k*_⊗**I**_*m*_)**f**+**δ**, where the covariance matrix **Σ** is represented by a factor analytic structure (**Σ** = **ΓΓ**^⊤^+**Ψ**). Therefore, model (1) can be rewritten as:

$$y=X\beta +Z\left(\Gamma_{p\times k}⊗I_{m}\right)f+Z\delta +\varepsilon$$ (2)

This has been referred to in the literature as the mixed model factor-analytic structured model or simply the factor analytic (FA) model. **Γ**_(*p*×*k*)_,**f**_(*mk*×1)_ and **δ**_(*mp*×1)_ are the loading matrix (*k* = 1, …,*p*), the factor vector related to additive genetic effects and the vector of specific effects, respectively. Furthermore, **ε** is the vector of residuals, **X** is the fixed-effect matrix referring to **β**, and **Z** is the genetic matrix referring to **f** and **δ**, where *k* represents the number of multiplicative terms. The **Z**_(*n*×*mp*)_ is a block diagonal matrix matching the vector **y**_(*n*×1)_; this is equivalent to assume that **Z** = (**Z**_1_,⋯,**Z**_*p*_) with **Z**_*k*_ the matrix matching *i*^th^ genotype at factor *k* with dimension (*n*×*m*), for *k* = 1,…,*p*, and $f=(f_{1}^{⊤},\cdots ,f_{p}^{⊤})$ with **f**_*k*_ a *m*-dimensional vector, then

$$Z\left(\Gamma ⊗I_{m}\right)f=\sum_{k=1}^{p}Z_{k}\Gamma f_{k},$$

Given that **ΓΓ**^⊤^ is a symmetric matrix, we can rewrite the model (2) observing that the matrix of factor loadings can be obtained by $\Gamma =V\Lambda^{\frac{1}{2}}$. In this expression, **V** represents the matrix of singular vectors and **Λ** a diagonal matrix formed by the eigenvalues obtained by the spectral decomposition of the loading matrix. The spectral decomposition of the unstructured covariance matrix can be approximated by

$$\Sigma =\Gamma \Gamma^{⊤}=V_{p}\Lambda_{p}V_{p}^{⊤}=\sum_{k=1}^{p}\lambda_{k}^{2}\alpha_{k}\alpha_{k}^{⊤}$$ (3)

where *λ*_*k*_ is the *i*-th singular value and **α**_*k*_ the *i*-th singular vector (or eigenvector) of the spectral decomposition, respectively.

A model with *k* = *p* (*p* as the matrix rank) multiplicative terms is called full rank, and the specific effects are assumed as null. However, the advantage of using factor analysis occurs when *k* is significantly smaller than *p*. If so, the number of parameters in the factor analysis *k*(*p*+1) becomes much smaller than those *p*(*p*+1)/2 parameters of **Σ**.

Using principal components to describe the factor loadings and considering the spectral decomposition properties and appropriate linear transformations, we can rewrite the Eq (2) that involves the loadings as follows:

$$ZL=Z\left(\Gamma_{m}⊗I_{p}\right)=\sum_{k=1}^{p}\lambda_{k}diag\left(X_{2}\alpha_{k}\right)Z_{f}$$ (4)

where $\Gamma_{m}=V_{p}\Lambda_{p}^{1/2}=\sum_{k=1}^{p}\lambda_{k}\alpha_{k}$. Further details on these properties and the theorem’s demonstration can be found in Songgui and Suju [32] and in (S1 Text). Replacing the Kronecker product into (2) with the sum in (4), the FA model can be expressed by

$$y=X_{1}\beta +\sum_{k=1}^{p}\lambda_{k}diag\left(X_{2}\alpha_{k}\right)Z_{f}f_{k}+Z\delta +\varepsilon$$ (5)

The terms *λ*_*k*_ and **α**_*k*_, as already specified in (3), are the *k-*th singular values and eigenvectors of the spectral decomposition of **Σ**, the matrices **X**_1_, **X**_2_, **Z** and **Z**_*f*_ are design matrices destined to distribute the FA effects for experimental unity (see S1 Text). By the summation, we can efficiently distribute the effects by avoiding using the Kronecker product or other artifices in the conditional distributions. This facilitates algebraic manipulations and computational implementations. Thus, the model presented in (5) is more treatable from a Bayesian point of view with respect to its equivalent (2). The conditional likelihood has a multivariate normal density as follows

$$y|\beta ,\lambda ,\alpha ,f,R∼N\left[X_{1}\beta +\sum_{k=1}^{p}\lambda_{k}diag\left(X_{2}\alpha_{k}\right)Z_{f}f_{k}+Z\delta ,R\right].$$

More details about the likelihood in factorial models can be obtained in supplemental material (S2 Text):

#### Prior distributions

In this study, the prior distributions for the FA parameters were established based on the assumptions of the factor analysis model through the maximum restricted likelihood (REML) [6]. In this sense, equivalent Jeffrey’s prior (Gaussian with large variance) was used for the fixed effects and Bayesian AMMI priors for the loading parameters approximated by the eigenvalues and eigenvectors. The prior distributions for each parameter were given by $\beta |\mu_{\beta },\sigma_{\beta }^{2}∼N(\mu_{\beta },I_{pq}\sigma_{\beta }^{2});I_{pq}$ is an identity matrix; **μ**_*β*_ = **0** and $\sigma_{\beta }^{2}=10^{12}\Rightarrow \beta ∼\mathrm{constant}.$

$f|\mu_{f},\sigma_{f}^{2}∼N\left(0,I_{m}\right)$, where: **I**_m_- is an identity matrix.

$\delta |\mu_{\delta },\sigma_{\delta }^{2}∼N\left(0,\Psi \right)$, where: **Ψ** = *diag*(*ψ*_11_,…,*ψ*_*kk*_), *k* = 1,…,p is a diagonal matrix in which the elements *ψ*_*ii*_ are the specific variances from each environment.

**α**_*k*_~Uniform spherical in the corrected subspace. The uniform spherical distribution is a special case of von Mises-Fisher distribution [15].

$\lambda_{k}|\mu_{\lambda_{k}},\sigma_{\lambda_{k}}^{2}∼N^{+}\left(0,\sigma_{\lambda_{k}}^{2}\right);\;\sigma_{\lambda_{k}}^{2}=10^{12}\;\Rightarrow \;\lambda_{k}|\mu_{\lambda_{k}},\sigma_{\lambda_{k}}^{2}∼constant$, where *N*^+^ indicates a positive Gaussian distribution truncated on *λ*_1_≥…≥*λ*_*p*_≥0.

Here, to simplify the notation, it will be assumed that **R** is a diagonal block matrix composed of $\sigma_{e_{k}}^{2}$, with *k =* 1, …, *p*. For this parameter, we assume an inverse scaled chi-squared prior distribution denoted by

$$\sigma_{e_{k}}^{2}∼Scale-\chi^{-2}\left(\nu_{e},S_{k}^{2}\right),\;\nu_{e}=0,S_{k}^{2}=0\;\Rightarrow \;\sigma_{e_{k}}^{2}∼\frac{1}{\sigma_{e_{k}}^{2}}$$

Since **Ψ** is also a diagonal matrix composed of diagonal elements *ψ*_*kk*_ with *k = 1* …, *p*, we also assumed the inverse scaled chi-squared prior distribution for *ψ*_*kk*_ as follows:

$$\psi_{kk}∼Scale-\chi^{-2}\left(\nu_{k},S_{k}^{2}\right),\;\nu_{k}=0,S_{k}^{2}=0\;\Rightarrow \;\psi_{kk}∼\frac{1}{\psi_{kk}}$$

All previous prior distributions satisfy the model constraints and are conditionally conjugated.

The Jeffrey’s prior for the fixed effects is |*I*_(*β*)_|^1/2^, where *I*_(*β*)_ is the expected Fisher information about **β**, used for environmental effects is proportional to a constant given that the information about **β** in the likelihood does not depends on **β**. Some equivalence is obtained in the posterior distribution by assuming a prior normal distribution with large variance, i.e 10^12^. For the singular values *λ*_*k*_, the truncated normal distributions were used in order to ensure the model identifiability for positive and sorted singular values, i.e., *λ*_*k*_>0 and *λ*_1_>*λ*_2_>…>*λ*_*k*_. For the singular vectors **α**_*k*_, the orthonormality restriction request that the coordinate be distributed on the hypersphere space; here an uniform hypersphere was assumed, in which is equivalent to a von Mises-Fisher with concentration parameter equal to zero[15].

For the factor model parameters (**f**, **δ**), the priors were assumed according to the classical factor model assumptions about this effects [6]. The prior hyperparameters for the variance components were chosen in order to impose large entropy and little weight of prior on the posterior distribution.

The data likelihood given above may be simplified as

$$L(y|\phi )=\frac{1}{\sqrt{2\pi^{n}\left|R\right|}}\exp\left\{-\frac{1}{2}{\left(y-\theta \right)}^{⊤}R^{-1}\left(y-\theta \right)\right\}$$ (6)

where *ϕ* = (**β,λ,δ,α,f,R**) and $\theta =X_{1}\beta +\sum_{k=1}^{m}\lambda_{k}diag\left(X_{2}\alpha_{k}\right)Z_{f}f_{k}+Z\delta$

#### Full conditional posterior distributions for the FA parameters

Applying Bayes’ theorem on the likelihood and priors for **φ**= (**β,λ,δ,α,f,R,Ψ**), the joint posterior distribution is given by

p(φ|y)∝L(y|ϕ)p(β)p(λ)p(α)p(δ)p(f)p(R)p(Ψ) (7)

The full conditional posterior distributions for the Bayesian factorial analytic (BFA) parameters were derived from (7) as follows:

i) Complete conditional a posteriori distribution for **β**

$$\beta |\ldots ∼N\left[{\left(X_{1}^{⊤}R^{-1}X_{1}\right)}^{-1}X_{1}^{⊤}R^{-1}A_{0},{\left(X_{1}^{⊤}R^{-1}X_{1}\right)}^{-1}\right]$$ (8)

where: $A_{0}=y-\sum_{k=1}^{m}\lambda_{k}diag\left(X_{2}\alpha_{k}\right)Z_{f}f_{k}-Z\delta$.

ii) Complete conditional a posteriori distribution for *λ*_*k*_

The full conditional posterior distribution for the singular value is denoted by:

$$\lambda_{k}|\ldots \propto N^{+}\left[{\left(A_{2k}^{⊤}R^{-1}A_{2k}\right)}^{-1}A_{2k}^{⊤}R^{-1}A_{1k},{\left(A_{2k}^{⊤}R^{-1}A_{2k}\right)}^{-1}\right]$$ (9)

where $A_{1k}=y-\left[X_{1}\beta +\sum_{k\neq k'}^{p}\lambda_{k'}diag\left(X_{2}\alpha_{k'}\right)Z_{f}f_{k'}+Z\delta \right]$ and **A**_2*k*_ = *diag*(**X**_2_**α**_*k*_)**Z**_*f*_**f**_*k*_.

iii) Complete conditional a posteriori distribution for **α**_*k*_

$$\alpha_{k}|\ldots ∼N\left[{\left(\Delta_{k}^{⊤}R^{-1}\Delta_{k}\right)}^{-1}\Delta_{k}^{⊤}R^{-1}A_{3k},{\left(\Delta_{k}^{⊤}R^{-1}\Delta_{k}\right)}^{-1}\right]$$ (10)

where $A_{3k}=y-X_{1}\beta -\sum_{k\neq k'}^{p}\lambda_{k'}diag\left(X_{2}\alpha_{k'}\right)Z_{f}f_{k'}-Z\delta$ and **Δ**_*k*_ = *λ*_*k*_*diag*(**Z**_*f*_,**f**_*k*_)**X**_2_.

Although the prior **α**_*k*_ presented spherical isotropy, it is not possible to obtain a conjugate von Mises-Fisher distribution similar to those obtained in Viele and Srivasan [33], Crossa et al. [17], or Oliveira et al. [18] for the AMMI model or in Crossa et al. [23] and Oliveira et al. [34] for the GGE model. This fact is because the previous-mentioned studies assumed the homogeneity of variances, which is different from the FA proposed here in which different variances were assumed for the environmental network. Instead, the conditional posterior will be a multivariate normal, which can violate the spectral decomposition constraints such as orthonormal eigenvectors.

To overcome these difficulties, the sampling will be performed in the corrected space free of the orthogonality constraint by using auxiliary variables (which will be defined in the further section); the vectors will be returned in the correct subspace in ${ℜ}^{m}$ through orthogonal linear transformations.

iv) Conditional a posteriori distribution for factor scores **f**

The complete posterior conditional distribution for **f** is given by

$$f|\ldots ∼N\left[{\left(I+A_{4}{}^{⊤}R^{-1}A_{4}\right)}^{-1}A_{4}{}^{⊤}R^{-1}A_{5};{\left(I+A_{4}{}^{⊤}R^{-1}A_{4}\right)}^{-1}\right]$$ (11)

where $A_{4}=\sum_{k=1}^{p}\lambda_{k}diag\left(X_{2}\alpha_{k}\right)Z_{f}$ and **A**_5_ = **y**−**X**_1_**β**−**Zδ**.

v) Complete conditional distribution a posteriori for specific variances **δ**

$$\delta |\ldots ∼N\left[{\left(Z^{⊤}V^{-1}Z+M^{-1}\right)}^{-1}Z^{⊤}R^{-1}A_{7},{\left(Z^{⊤}V^{-1}Z+M^{-1}\right)}^{-1}\right]$$ (12)

where $A_{6}=\sum_{k=1}^{p}\lambda_{k}diag\left(X_{2}\alpha_{k}\right)Z_{f}f_{k}$, **A**_7_ = **y**−**X**_1_**β**−**A**_6_ and **M** = **I**_m_⊗**Ψ**.

vi) Complete conditional a posteriori distribution of **Ψ**

Since **Ψ** is diagonal matrix with the independent elements **Ψ** = *diag*(*ψ*_11_,…,*ψ*_*kk*_) with *k = 1*,*…*,*p*, it was considered a scaled inverse chi-squared distribution for each diagonal element. The complete conditional distribution posterior for each *ψ*_*kk*_ is

$$\psi_{kk}|\ldots ∼Scale-\chi^{-2}\left(m_{k}+\nu_{k},\frac{m_{k}\left(\delta^{⊤}\delta /m_{k}\right)+\nu_{k}S_{k}^{2}}{m_{k}+\nu_{k}}\right)$$ (13)

where *m*_*k*_ is the number of genotypes in the environment and *ν*_*k*_ = 1.

vii) Complete conditional a posteriori distribution for $\sigma_{e_{k}}^{2}$

Similar to **Ψ**, the **R** is also a diagonal matrix and the independent priors also have scaled inverse chi-squared distributions. The conditional posterior distribution obtained for each $\sigma_{e_{k}}^{2}$ is given by

$$\sigma_{e_{k}}^{2}|\ldots ∼Scale-\chi^{-2}\left[n_{k},\frac{{\left(y_{k}-\theta_{k}\right)}^{⊤}\left(y_{k}-\theta_{k}\right)}{n_{k}}\right]$$ (14)

where *n*_*k*_ is the number of observations in each environment.

#### Sampling parameters requesting orthonormal basis

As all conditional distributions were obtained in a closed form, the distributions have known shapes that allow for direct sampling using the Gibbs sampler. However, as was previously highlighted, the conditional distribution for the vector **α**_*k*_ is a multivariate Gaussian distribution instead of a von Mises-Fisher. Nevertheless, this distribution and its parametric space do not ensure the model’s constraints since the vectors must have unitary norms and be orthogonal to each other.

However, the sampling can still be performed from the normal multivariate in the corrected subspace by adding two further steps: normalize the **α**_*k*_ vector and return it into the orthogonal basis using the correct subspace through a linear transformation. Viele and Srinivasan [33] have shown how to perform the sampling of the spherical uniform distribution using the standardized Gaussian distribution and how the vectors can be placed in the correct subspace using the Gram–Schmidt orthonormalization process.

Assuming that **α**_*k*_ is an unit vector of *m-s* dimension in space ${ℜ}^{m}$, $\left(\alpha_{k}^{⊤}\alpha_{k}=1\right)$ is orthogonal to vector *S (v*_*1*_, *v*_*2*_, …, *v*_*s*_*)* in ${ℜ}^{m}$. Further suppose that the vectors **α**_1_,**α**_2_,…,**α**_*s*_ are a set of orthonormal vectors in the subspace generated by *v*_*1*_, *v*_*2*_, …, *v*_*s*_. In these terms, given the matrix **H**_*s*_ = **α**_1_,**α**_2_,…,**α**_*s*_, there is a matrix **H**_*k*_ with the dimensions *m*×(*m*−*s*) so that **H**_*m*_ = (**H**_*s*_,**H**_*k*_) is an orthonormal matrix. This matrix can be obtained by the *Gram-Schmidt* orthonormalization process.

Thereby, we obtained the vector ${\tilde{\alpha }}_{k}$ by the linear transformation ${\tilde{\alpha }}_{k}=H_{k}^{⊤}\alpha_{k}$ so that ${\tilde{\alpha }}_{k}\in {ℜ}^{m-s}$. In other words, the sampling will be performed in the "corrected" subspace without the constraints imposed by the spectral decomposition. We can easily show that $\alpha_{k}=H_{k}^{}{\tilde{\alpha }}_{k}$. Therefore, we retrieve the vector in the correct subspace in ${ℜ}^{m}$ and orthogonal to *s* vectors by applying the inverse operation, satisfying the model’s constraints.

The posterior distribution for the sampling process can be obtained from the kernel of the full conditional posterior distribution of **α**_*k*_ as follows:

$$\exp\left\{-\frac{1}{2}\left({\left[\alpha_{k}-{\left(\Delta_{k}^{⊤}R^{-1}\Delta_{k}\right)}^{-1}\Delta^{⊤}R^{-1}A_{3k}\right]}^{⊤}\left(\Delta_{k}^{⊤}R^{-1}\Delta_{k}\right)\left[\alpha_{k}-{\left(\Delta_{k}^{⊤}R^{-1}\Delta_{k}\right)}^{-1}\Delta_{k}^{⊤}R^{-1}A_{3k}\right]\right)\right\}$$

By solving within the brackets, we have

$$\begin{array}{l} \alpha_{k}^{⊤}\left(\Delta_{k}^{⊤}R^{-1}\Delta_{k}\right)\alpha_{k}-\alpha_{k}^{⊤}\left(\Delta_{k}^{⊤}R^{-1}\Delta_{k}\right){\left(\Delta^{⊤}R^{-1}\Delta_{k}\right)}^{-1}\Delta_{k}^{⊤}R^{-1}A_{3k}-\\ -{\left[{\left(\Delta_{k}^{⊤}R^{-1}\Delta \right)}^{-1}\Delta_{k}^{⊤}R^{-1}A_{3k}\right]}^{⊤}\left(\Delta_{k}^{⊤}R^{-1}\Delta_{k}\right)\alpha_{k}+\\ +{\left[{\left(\Delta_{k}^{⊤}R^{-1}\Delta_{k}\right)}^{-1}\Delta_{k}^{⊤}R^{-1}A_{3k}\right]}^{⊤}\left(\Delta_{k}^{⊤}R^{-1}\Delta_{k}\right){\left(\Delta_{k}^{⊤}R^{-1}\Delta_{k}\right)}^{-1}\Delta_{k}^{⊤}R^{-1}A_{3k} \end{array}$$

Using the identity $H_{k}H_{k}^{⊤}=I$ and dividing each part of the kernel, we get

- $\alpha_{k}^{⊤}H_{k}H_{k}^{⊤}\left(\Delta_{k}^{⊤}R^{-1}\Delta_{k}\right)H_{k}H_{r}^{⊤}\alpha_{k}={\left(H_{k}^{⊤}\alpha_{k}\right)}^{⊤}H_{r}^{⊤}\left(\Delta_{k}^{⊤}R^{-1}\Delta_{k}\right)H_{k}H_{k}^{⊤}\alpha_{k}=$
  $={\left({\tilde{\alpha }}_{k}\right)}^{⊤}H_{k}^{⊤}\left(\Delta_{k}^{⊤}R^{-1}\Delta_{k}\right)H_{k}{\tilde{\alpha }}_{k}$
$-\alpha_{k}^{⊤}\left(\Delta_{k}^{⊤}R^{-1}\Delta_{k}\right){\left(\Delta_{k}^{⊤}R^{-1}\Delta_{k}\right)}^{-1}\Delta_{k}^{⊤}R^{-1}A_{3k}=$
  $=-\alpha_{k}^{⊤}H_{k}H_{k}^{⊤}\left(\Delta_{k}^{⊤}R^{-1}\Delta_{k}\right)H_{k}H_{k}^{⊤}{\left(\Delta_{k}^{⊤}R^{-1}\Delta_{k}\right)}^{-1}\Delta_{k}^{⊤}R^{-1}A_{3k}=$ $=-{\tilde{\alpha }}_{k}^{⊤}H_{k}^{⊤}\left(\Delta_{k}^{⊤}R^{-1}\Delta_{k}\right)H_{k}H_{k}^{⊤}{\left(\Delta_{k}^{⊤}R^{-1}\Delta_{k}\right)}^{-1}\Delta_{k}^{⊤}R^{-1}A_{3k}$
$-{\left[{\left(\Delta_{k}^{⊤}R^{-1}\Delta_{k}\right)}^{-1}\Delta_{k}^{⊤}R^{-1}A_{3k}\right]}^{⊤}\left(\Delta_{k}^{⊤}R^{-1}\Delta_{k}\right)H_{k}H_{k}^{⊤}\alpha_{k}=$
  $=-{\left[{\left(\Delta_{k}^{⊤}R^{-1}\Delta_{k}\right)}^{-1}\Delta_{k}^{⊤}R^{-1}A_{3k}\right]}^{⊤}\left(\Delta_{k}^{⊤}R^{-1}\Delta_{k}\right)H_{k}H_{k}^{⊤}\alpha_{k}=$ $=-{\left[{\left(\Delta_{k}^{⊤}R^{-1}\Delta_{k}\right)}^{-1}\Delta_{k}^{⊤}R^{-1}A_{3k}\right]}^{⊤}H_{k}^{⊤}\left(\Delta_{k}^{⊤}R^{-1}\Delta_{k}\right)H_{k}{\tilde{\alpha }}_{k}$
- ${\left[{\left(\Delta_{k}^{⊤}R^{-1}\Delta_{k}\right)}^{-1}\Delta_{k}^{⊤}R^{-1}A_{3k}\right]}^{⊤}\left(\Delta_{k}^{⊤}R^{-1}\Delta_{k}\right){\left(\Delta_{k}^{⊤}R^{-1}\Delta_{k}\right)}^{-1}\Delta_{k}^{⊤}R^{-1}A_{3k}=$
  $={\left[{\left(\Delta_{k}^{⊤}R^{-1}\Delta_{k}\right)}^{-1}\Delta_{k}^{⊤}R^{-1}A_{3k}\right]}^{⊤}H_{k}H_{k}^{⊤}\left(\Delta_{k}^{⊤}R^{-1}\Delta_{k}\right)H_{k}H_{k}^{⊤}{\left(\Delta_{k}^{⊤}R^{-1}\Delta_{k}\right)}^{-1}\Delta_{k}^{⊤}R^{-1}A_{3k}$
  In this way,
  $\alpha_{k}^{⊤}\left(\Delta_{k}^{⊤}R^{-1}\Delta_{k}\right)\alpha_{k}-\alpha_{k}^{⊤}\left(\Delta_{k}^{⊤}R^{-1}\Delta_{k}\right){\left(\Delta_{k}^{⊤}R^{-1}\Delta_{k}\right)}^{-1}\Delta_{k}^{⊤}R^{-1}A_{3k}-$ $-{\left[{\left(\Delta_{k}^{⊤}R^{-1}\Delta_{k}\right)}^{-1}\Delta_{k}^{⊤}R^{-1}A_{3k}\right]}^{⊤}\left(\Delta_{k}^{⊤}R^{-1}\Delta_{k}\right)\alpha_{k}+$ $+{\left[{\left(\Delta_{k}^{⊤}R^{-1}\Delta_{k}\right)}^{-1}\Delta_{k}^{⊤}R^{-1}A_{3k}\right]}^{⊤}\left(\Delta_{k}^{⊤}R^{-1}\Delta_{k}\right){\left(\Delta_{k}^{⊤}R^{-1}\Delta_{k}\right)}^{-1}\Delta_{k}^{⊤}R^{-1}A_{3k}=$ $={\left({\tilde{\alpha }}_{k}\right)}^{⊤}H_{k}^{⊤}\left(\Delta_{k}^{⊤}R^{-1}\Delta_{k}\right)H_{k}{\tilde{\alpha }}_{k}-{\tilde{\alpha }}_{k}^{⊤}H_{k}^{⊤}\left(\Delta_{k}^{⊤}R^{-1}\Delta_{k}\right)H_{k}H_{k}^{⊤}{\left(\Delta_{k}^{⊤}R^{-1}\Delta_{k}\right)}^{-1}\Delta_{k}^{⊤}R^{-1}A_{3k}-$ $-{\left[{\left(\Delta_{k}^{⊤}R^{-1}\Delta_{k}\right)}^{-1}\Delta_{k}^{⊤}R^{-1}A_{3k}\right]}^{⊤}H_{k}H_{k}^{⊤}\left(\Delta_{k}^{⊤}R^{-1}\Delta_{k}\right)H{\tilde{\alpha }}_{k}+$ ${\left[{\left(\Delta_{k}^{⊤}R^{-1}\Delta_{k}\right)}^{-1}\Delta_{k}^{⊤}R^{-1}A_{3k}\right]}^{⊤}H_{k}H_{k}^{⊤}\left(\Delta_{k}^{⊤}R^{-1}\Delta_{k}\right)H_{k}H_{k}^{⊤}{\left(\Delta_{k}^{⊤}R^{-1}\Delta_{k}\right)}^{-1}\Delta_{k}^{⊤}R^{-1}A_{3k}$

Thus, the conditional posterior for the auxiliary variable ${\tilde{\alpha }}_{k}$ given the other parameters in the corrected subspace is given by

$${\tilde{\alpha }}_{k}|\ldots ∼N\left[H_{k}^{⊤}{\left(\Delta_{k}^{⊤}R^{-1}\Delta_{k}\right)}^{-1}\Delta_{k}^{⊤}R^{-1}A_{3k},{\left(H_{k}^{⊤}\left(\Delta_{k}^{⊤}R^{-1}\Delta_{k}\right)H_{k}\right)}^{-1}\right].$$

Therefore, the sampling of ${\tilde{\alpha }}_{k}$ is performed in the corrected subspace through the previously obtained conditional. As presented in Crossa et al. [17] and Oliveira et al. [18], we seek the sample vectors that have the norm 1. Thus, the orthogonal vectors must be normalized as $\alpha_{k}^{*}=\frac{{\tilde{\alpha }}_{k}}{\sqrt{{\tilde{\alpha }}_{r}^{⊤}{\tilde{\alpha }}_{k}}}$. Assuming ${\bar{\tilde{\alpha }}}_{k}=H_{k}^{⊤}{\left(\Delta_{k}^{⊤}R^{-1}\Delta_{k}\right)}^{-1}\Delta_{k}^{⊤}R^{-1}A_{3k}$ and $C_{k}=\sqrt{{\tilde{\alpha }}_{r}^{⊤}{\tilde{\alpha }}_{k}}$, through algebraic manipulations, the conditional distribution for the orthonormal ${\tilde{\alpha }}_{k}$ is given by

$${\tilde{\alpha }}_{k}|\ldots ∼N\left[\alpha_{k}^{*},{\left(C_{k}^{⊤}H_{k}^{⊤}\left(\Delta_{k}^{⊤}R^{-1}\Delta_{k}\right)H_{k}C_{k}\right)}^{-1}\right]$$

where $\alpha_{k}^{*}=\frac{{\tilde{\alpha }}_{k}}{C_{k}}$.

To place the singular vector in the correct subspace ${ℜ}^{m}$ satisfying the orthonormal constraints, we apply the inverse transformation $\alpha_{k}=H_{k}^{}{\tilde{\alpha }}_{k}$. The sampled vector is now orthogonal to the other *s* vectors and its transformation preserves the vector norm since

$${\left({\tilde{\alpha }}_{k}\right)}^{⊤}{\tilde{\alpha }}_{k}={\left(H_{k}^{⊤}\alpha_{k}\right)}^{⊤}H_{k}^{⊤}\alpha_{k}=\alpha_{k}^{⊤}H_{R}H_{R}^{⊤}\alpha_{k}=\alpha_{k}^{⊤}I_{m}\alpha_{k}^{⊤}=\alpha_{k}^{⊤}\alpha_{k}=1.$$

Thus, the random *m-s* dimensional vector in ${ℜ}^{m}$ isone-to-one transformation into the same random vector in ${ℜ}^{m-s}$.

From the previously complete conditional distributions, the parameter sampling was performed by the Markov chain Monte Carlo (MCMC) using the Gibbs sampler. The implemented iterative sampling algorithm is illustrated in S3 Text.

After concluded the iterative process and checked the chains’ convergence, the samples were considered to have resulted from the marginal densities. The convergence diagnostic was performed using the Raftery and Lewis [35] Heidelberger and Welch [36] criterion. All inference processes were performed using the R statistical software [37].

#### Inference about the linear and multiplicative parameters of the model

The sampled eigenvectors present orthonormal basis across MCMC process, however, their maximum posterior estimator (MAP) may not be orthogonal. The MAP estimator for the multiplicative terms were constructed according to the method proposed by Chen and Shao [38] implemented in the Bayesian output analysis (BOA) package using the R statistical software (R CORE TEAM, 2016).

#### Bivariate regions of credibility for factor loadings and scores

The biplot credibility regions for factor loadings (*λ*_1_**α**_1_,*λ*_2_**α**_2_) and factor scores (**f**_1_,**f**_2_) were constructed using the Euclidean distances of the sampling points with respect to the distribution center using 5% as the cut off [39].

The FA biplot interpretation was performed similarly to the GGE-biplot, as suggested by Burgueño et al. [7].

#### Model validation in the prediction of missing data

The cross-validation process was performed considering different levels of missing data in the GEI matrix. The sample was randomly divided into *k*-fold of equal size, with *k* = (10, 3, 2) corresponding respectively to the 10%, 33% and 50% levels of random genotype losses in the environments without replacement. Thus, the simulated missing was performing on GEI table cells where some genotypes (lines) information were totally withdraw from specific environments (columns), but keeping all environments in the dataset. In addition, the cross-validation was performed to allow that all genotypes were evaluated in at least one environment. Therefore, the GEI cells were randomly sampled, but observing the restrictions given above.

The BFA’s predictive ability was compared to the two-step FA models using the EM algorithm (FA-EM—expectation maximization) (Nuvunga et al. [40] and the FA via AI algorithm (Average information) (FA-AI) (Smith, Cullis and Thompson [6]) in sparse matrices implemented in Asreml-R [41].

The evaluation of the model’s predictive ability was performed using the average PRESS (*predicted residual error sum of squares*) and phenotypic correlation between the predicted $\left({\hat{y}}_{ij}\right)$ and observed (*y*_*ij*_) values. The PRESS expression is given by

$$PRESS=\frac{1}{n}\sum_{j=1}^{n}{\left(y_{ij}-{\hat{y}}_{ij}\right)}^{2}$$ (15)

#### Model selection or choice of the number of factors *k*

The number of latent factors to be retained in the model was selected using the PRESS criterion, which uses a cross-validation approach [42].The statistical efficiency criterion is given by *SE* = (*PRES*_*full*_)/(*PRESS*_*k*_), which is the ratio between the PRESS of the full model and the PRESS of low-order models.

The model selection for real data was done using the Akaike Information Criterion Monte Carlo (AICM). Δ*AICM* corresponds to the difference between the full model and the competing models, as suggested by Raftery et al. [43]. The AICM is calculated as a version based on a posteriori simulation of the AIC [44].

$$AICM=2\left(\bar{l}-s_{l}^{2}\right)$$ (16)

where $\bar{l}$ is the mean of the marginal log-likelihood and $s_{l}^{2}$ is the posterior variance of the marginal log-likelihood.

Thus, the AICM can be seen as the simplified version of the penalized posterior mean of the log-likelihood. The selected model is the one with the highest AICM and lowest Δ*AICM*.

## Results

### Simulated data

For this scenario, MCMC chains with 65,560 iterations were simulated for the BFA model. As already pointed out, the convergence of the generated chains was monitored by the criteria Raftery and Lewis and Heidelberger and Welch. The first 8,400 observations were burned-in and a thinning for each four observations was performed to ensure the convergence process. The values of burning and thinning were based on a training sample according to the test of Raftery and Lewis. It was also observed that all parameters had a dependence factor I <5. The final chain length was 14,290 samples for each parameter.

In addition, all parameters passed the stationarity test, indicating that convergence was achieved according to the criterion of Heidelberger and Welch and Geweke. That is, the tests indicated good convergence properties for all model parameters. In S4 Text, the traces for the residual variance chains are shown and its pattern corroborates the convergence test results. Another interesting detail, in the Bayesian context, is the computational time of analysis, which for the simulated data was 22.15 minutes.

The simulated parametric values for the residual variance and FA loadings *τ*_*k*_ (recovered by *λ*_*k*_*α*_*k*_) and the MAP estimates are presented in Table 1. We notice that the estimated values and parametric values do not present large differences, which support the BFA’s ability in estimating the loadings of FA models. All values used in the simulations are within the 95% credibility intervals.

**Table 1:** Bayesian maximum a posterior (MAP), simulated parametric value (PV), posterior standard deviation (PSD), credibility intervals (CI 95%, LL: Lower limit, UL: Upper limit).

				HPD 95%	
Parameter	PV	MAP	PSD	LL	UL
$\sigma_{e_{1}}^{2}$	0.546	0.847	1.260	0.299	1.506
$\sigma_{e_{2}}^{2}$	1.209	1.911	1.155	0.846	3.162
$\sigma_{e_{3}}^{2}$	4.690	4.381	2.353	2.209	6.826
$\sigma_{e_{4}}^{2}$	7.377	10.091	19.936	4.938	15.097
$\sigma_{e_{5}}^{2}$	9.026	9.104	3.807	3.503	15.793
$\sigma_{e_{}}^{2}$	2.900	2.848	-	-	-
*λ*_1_*α*_11_	1.468	1.548	0.845	0.500	3.448
*λ*_1_*α*_21_	1.314	1.344	0.761	0.291	3.062
*λ*_1_*α*_31_	2.404	2.353	1.267	0.799	4.916
*λ*_1_*α*_31_	1.771	1.475	1.008	-0.439	3.817
*λ*_1_*α*_51_	2.751	2.191	1.311	0.180	5.022
*λ*_2_*α*_12_	0.579	0.381	0.603	-0.845	1.521
*λ*_2_*α*_22_	0.385	0.436	0.374	0.000	1.154
*λ*_2_*α*_32_	0.257	0.234	0.678	-1.093	1.657
*λ*_2_*α*_42_	1.717	0.307	0.890	-1.466	2.113
*λ*_2_*α*_52_	-1.823	-0.764	1.154	-2.726	1.748

Residual variance mean $\left(\sigma_{e_{k}}^{2}\right)$ simulated factor loadings *τ*_*k*_ and recovered by the BAF model (*τ*_*k*_ = *λ*_*k*_*α*_*k*_).

Specific estimates and credibility confidence regions for the coordinates related to the first two factor scores can be seen in (S2 Table). The posterior estimates presented values very close to those from FA mixed models obtained by the restricted maximum likelihood (REML) estimates using the average information (FA-AI) algorithm. However, the second axes for both methods present large differences for G1, G2 and G5, but they are within the credibility intervals.

**Fig 1:**
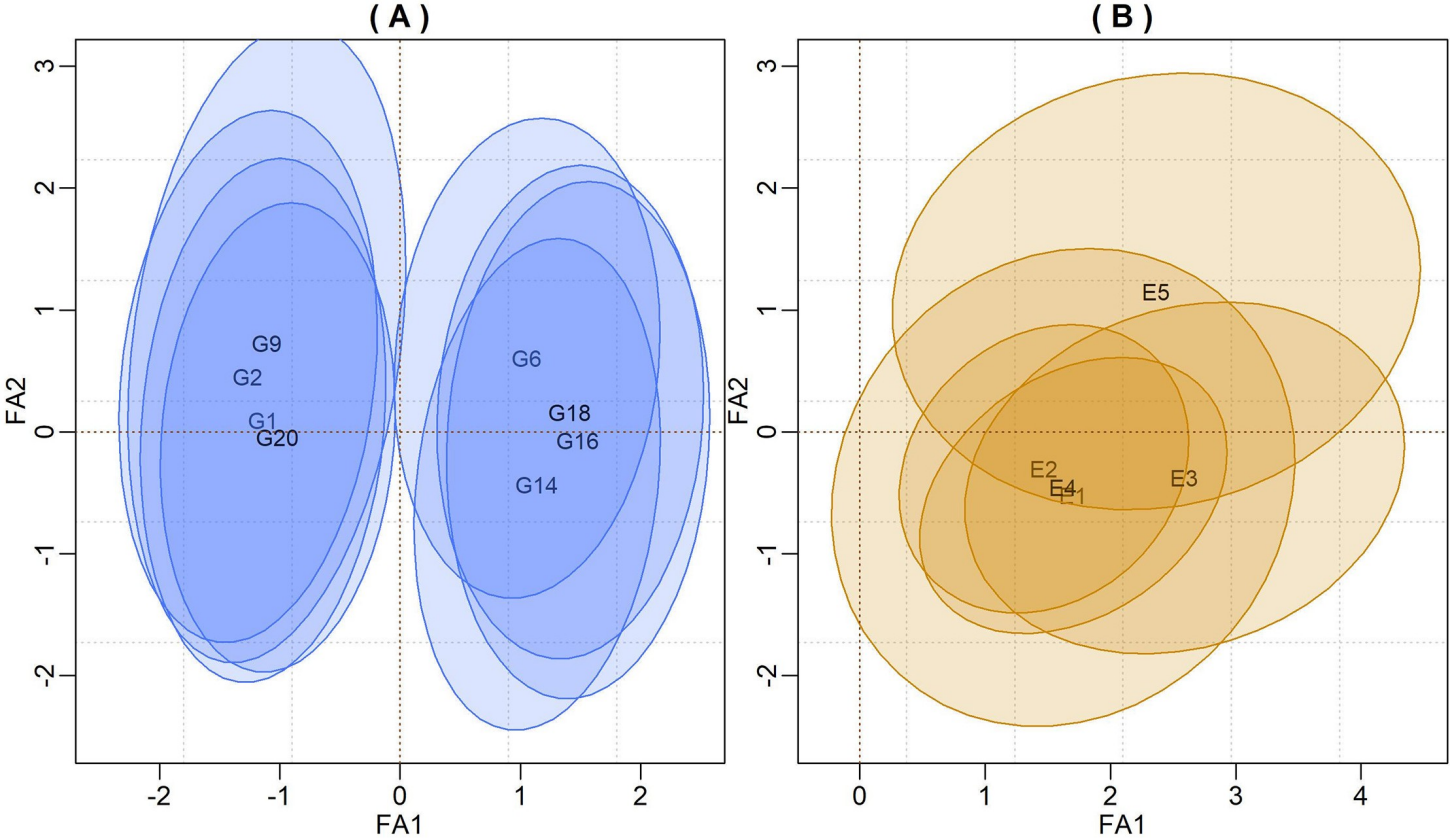
Credibility regions at 95% probability for genotypic factor scores (Fig 1A) and for the factor loadings of environments (Fig 1B) using the first two components. Only the genotypes scores that did not include the biplot origin were represented.

The bivariate credibility regions (highest posterior density—HPD) for the factor loadings (*λ*_1_**α**_1_,*λ*_2_**α**_2_) and genotypic scores (**f**_1_,**f**_2_) that did not included the biplot origin (0,0) are shown in Fig 1. From these biplots, we see the clustering groups with respect to grain yields and/or GEI patterns. In FA models where G is confounded with GE, the first loading tends to present a positive signal (Fig 1B). According to Burgueño et al. [7], Smith, Cullis and Thompson [6] and Stefanova and Buirchell [29], in these situations, the interpretation for the FA biplot is similar to the GGE-biplot. However, it is necessary to be careful when interpreting the intersections among the credibility regions, given that loadings and scores do not have inner-product properties. If so, the biplots are better justified with separate presentations for loading and factor scores to avoid confusion with the AMMI or GGE models.

However, just to illustrate the usefulness of the BFA model, we plotted both factor loadings and scores in a single biplot (Fig 1A). In this Figure, it is possible to see two distinct groups of genotypes with respect to yields, but they are similar with respect to the GEI pattern since all regions cross the first axis.

Fig 1B shows the HPD’s credible regions for factor loadings at 95% confidence. This subgroup of environments has similar effects with respect to the GEI pattern, as indicated by the overlaps between the credibility regions and ellipses that did not encompassed the biplot origin.

Given that the first axis captures much of the main effect of genotypes and the second axis captures the complex part related to the GEI, it was observed that the scale and ranking of the factor scores were equivalent to the marginal E-BLUPS obtained from the mixed model analysis since the regression adjustment between the two estimates was approximately 1 (r^2^ = 0.979).

#### Performance evaluation of the unbalanced model

In addition to evaluating the BFA model in terms of parameter estimations, the Bayesian FA model’s predictive ability was evaluated and compared to the two step FA model [40], and FA-AI [41]. Although it is difficult to adjust a full FA model in the mixed models framework due to computational costs and convergence problems, this problem was not observed for the simulated dataset, given that the set of environments is relatively lower compared to the data set commonly utilized in MET.

The cross-validation results showed that it is possible to predict the performance of hybrids using FA models with high accuracy, reaching up to 0.82 in some folds, as explained in S1–S6 Figs.

**Fig 2:**
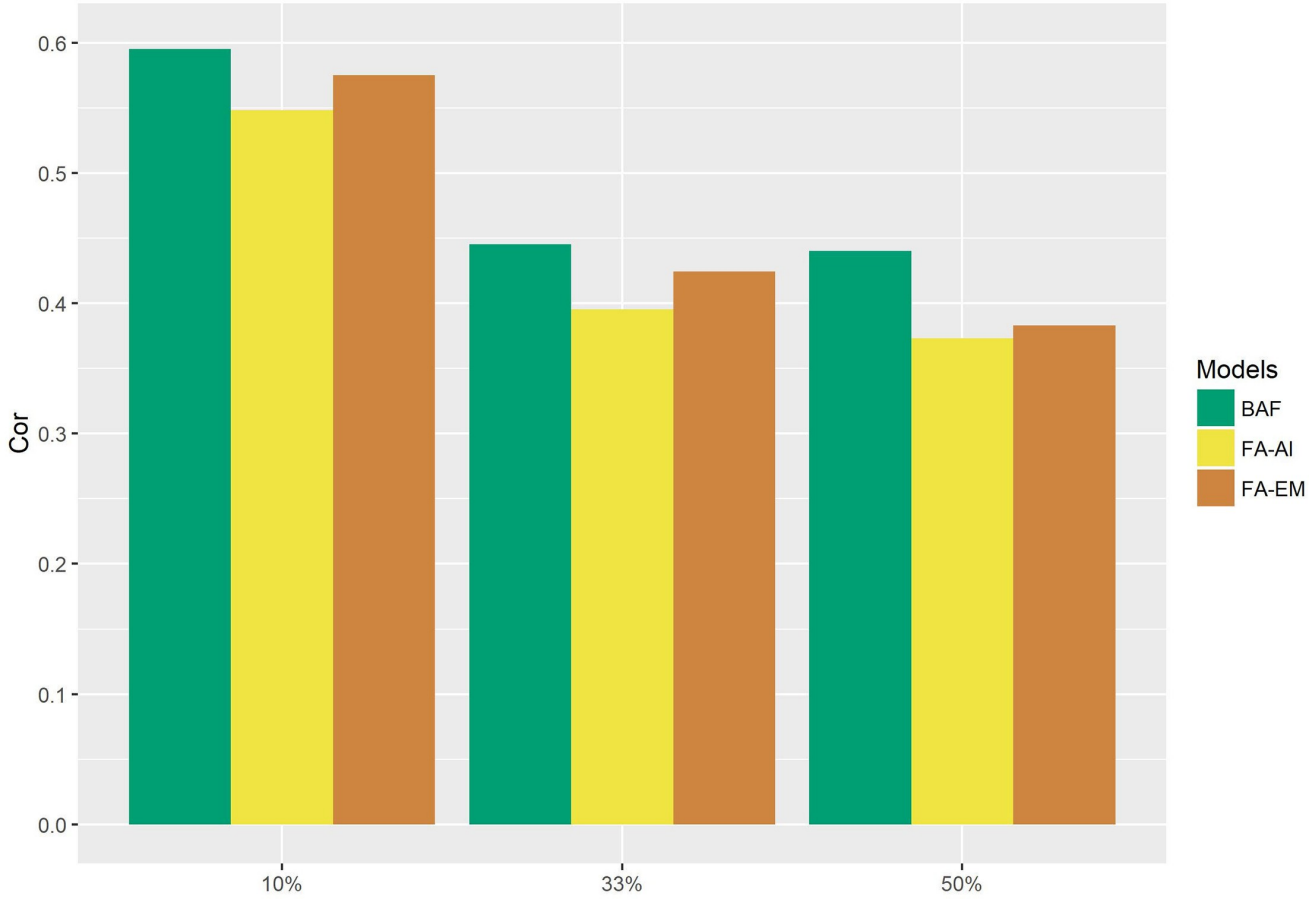
Bar chart related to the correlation for 10-fold, 3-fold and 2-fold scenarios using the Bayesian FA (BAF) and FA models (FA-EM and FA-AI) for simulated data.

Regardless of the unbalanced level applied to the hybrid panel, the magnitude of the correlation values was higher than 0.30 (Fig 2) and the Bayesian model showed the highest prediction ability for all scenarios.

**Fig 3:**
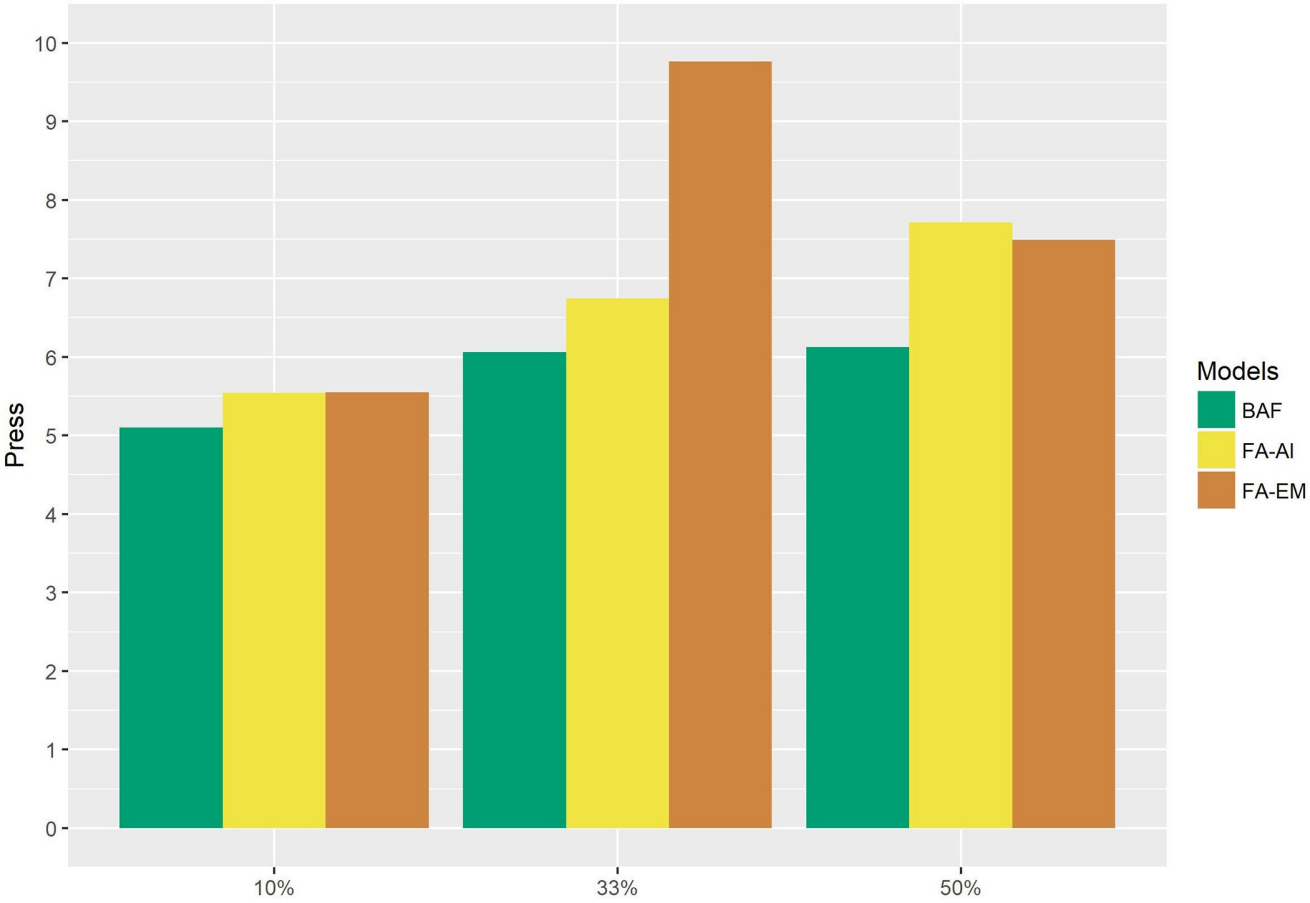
Bar chart related to the PRESS for 10-fold, 3-fold and 2-fold scenarios using the Bayesian FA (BAF) and FA models (FA-EM and FA-AI) for simulated data.

The PRESS in the10-fold scenario (as expected) was lowest when compared to the other folds for the three models (Fig 3). At all considered unbalanced levels, the BAF model had the lowest PRESS. At 10%, the FA-AI and FA-EM models had the same PRESS and alternated precision at the 33% and 50% levels. In other words, the scenarios proposed to PRESS using these two FA approaches were inconclusive.

#### FA model selection based on cross validation

The numbers of latent factors to be retained in the model were selected using a 10-fold cross-validation. Therefore, the FA5, FA4, FA3, FA2 and FA1 models were adjusted and the model selection was based on the PRESS criterion and statistical efficiency (SE) (defined as the ratio between the full FA model versus the low-dimension ones).

**Fig 4:**
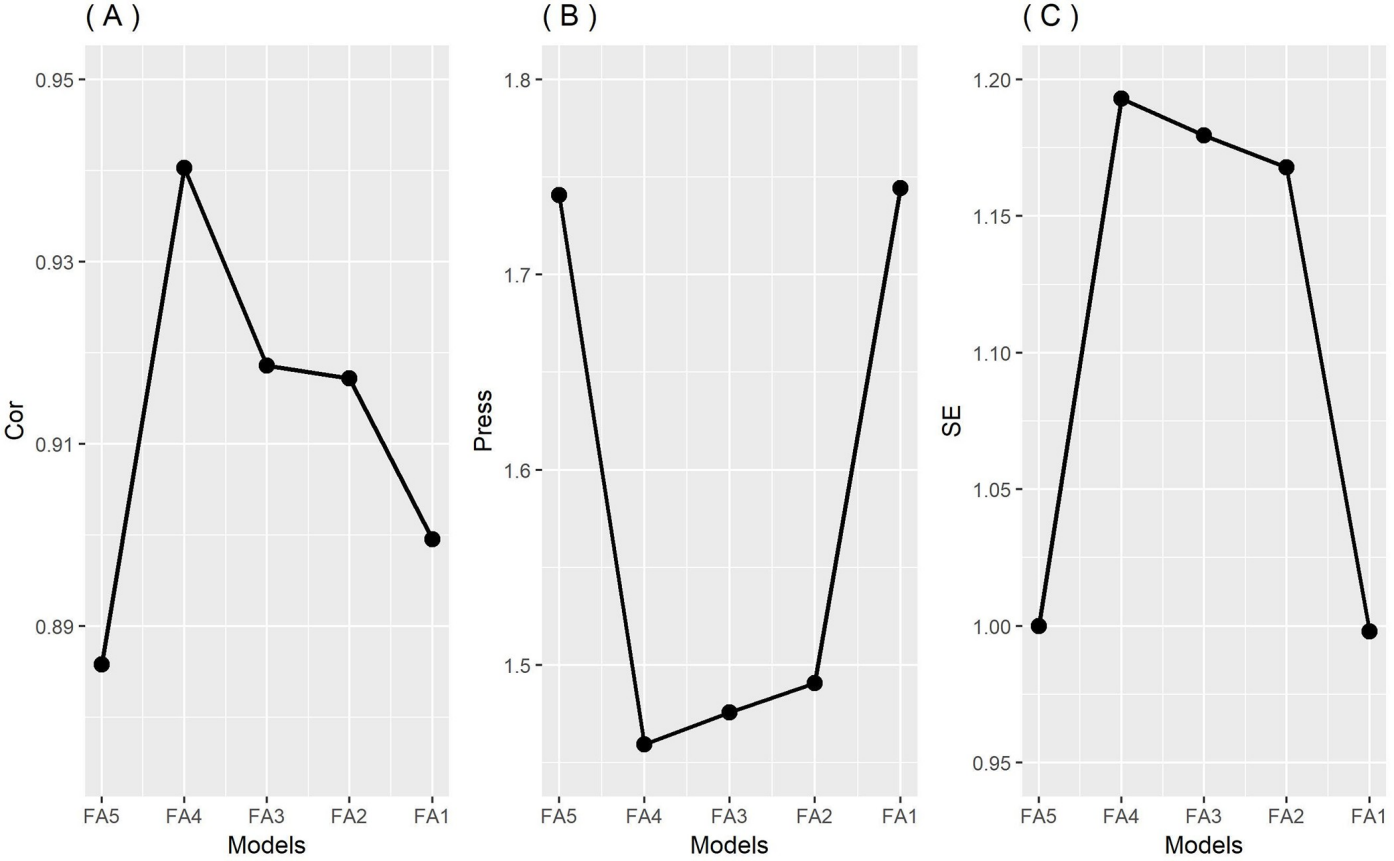
Ockham's plot referring to the BAF model performance using the correlation between the observed and predicted phenotypic values (A) (higher, better), the predicted sum square (PRESS) (B) (lower, better) and the statistical efficiency (SE) (C) (higher, better) for simulated data.

Fig 4B and 4A present the PRESS and the correlation between the simulated phenotypic value and the predicted one using each model. Observing Fig 4B, one can verify that the FA4 model (with *k =* 4) presented the highest predictive accuracy compared to the others candidate models, indicating that this model is the best one. Additionally, using the PRESS criterion, the best model again was FA4 (Fig 4A).

Fig 4C shows the graphic of the statistical efficiency of the competing models. In this graphic, it is notable that the efficiency increases from FA1 to FA4 where we find the maximum efficiency. This demonstrates that the use of *k* = 4 would be the best choice for these data representation.

The FA2 model is generally considered parsimonious and interpretable for plant breeders, since the first axis can be seen as related to adaptability and the second related to stability, such as in the SREG2/GGE model [7,29]. However, it was verified that the FA4 model showed better results in the three used criteria.

### Experimental data

For the real data scenario, 85,000 Markov chains were simulated and (similar to the previous analysis), the first 9,800 observations were burned-in and thinned for every eight samples. A final MCMC chain with 9,400 observations was obtained for each parameter. The chain convergence was verified by Raftery and Lewis [35], Heidelberger and Welch [36] and and Geweke criterion [45]. The trace pattern related to the posterior distributions also indicated good convergence. The computational time spent in the MCMC sampling process was 13,938.34 minutes.

The point and interval estimates for the components of variance $\sigma_{k}^{2}$ are presented in Table 2. It is possible to notice that the environments E8 and E2 presented the highest and lowest residual variances, respectively.

**Table 2:** Posterior means (PM), credibility regions (CI 95%, LL: Lower limit, UL: Upper limit) for the residual variance $\left(\sigma_{e_{k}}^{2}\right)$ obtained by the BFA model for real data.

			HPD 95%	
Parameter	PM	SD	LL	UL
$\sigma_{e_{1}}^{2}$	3.4642	0.2869	2.9392	3.9960
$\sigma_{e_{2}}^{2}$	1.4465	0.2187	1.2076	1.6734
$\sigma_{e_{3}}^{2}$	1.9769	0.1655	1.6827	2.2918
$\sigma_{e_{4}}^{2}$	1.6672	0.1634	1.3776	1.9581
$\sigma_{e_{5}}^{2}$	3.2711	0.3030	2.7526	3.7986
$\sigma_{e_{6}}^{2}$	1.8623	0.1538	1.5850	2.1727
$\sigma_{e_{7}}^{2}$	2.1392	0.1968	1.8324	2.4674
$\sigma_{e_{8}}^{2}$	4.0689	0.2984	3.4846	4.6402
$\sigma_{e_{9}}^{2}$	1.6871	0.1472	1.4090	1.9773
$\sigma_{e_{10}}^{2}$	3.3718	0.2624	2.8713	3.8595

**Fig 5:**
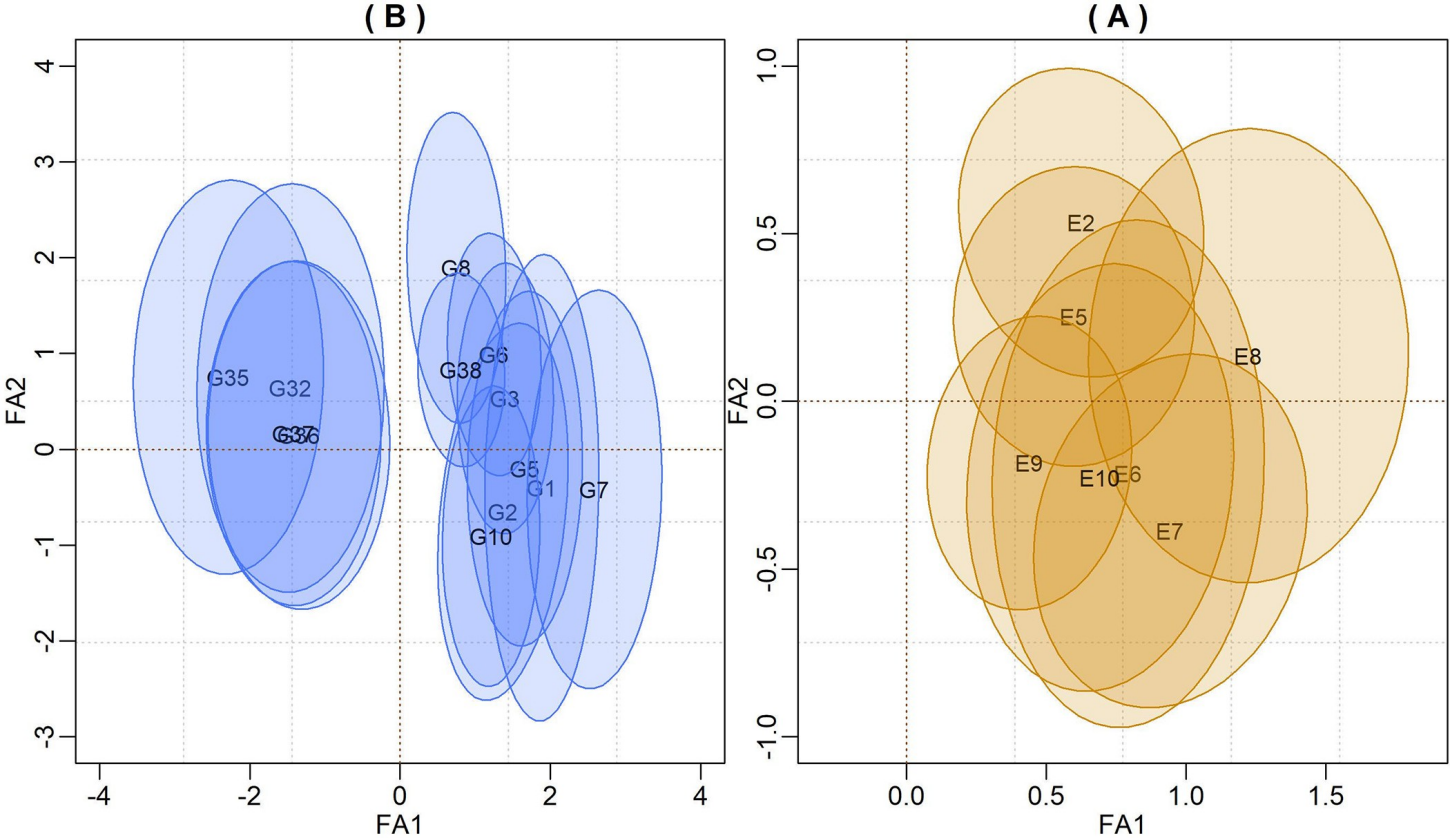
Credibility regions at the 95% probability for genotypic factor scores (a) and for factor loadings of environments (b) using the Bayesian approach for real data. Only the intervals that did not include the biplot origin were represented.

Fig 5A shows the HPD credibility regions at the 95% probability for the genotypic factor scores. For simplicity, only genotypes that did not include the biplot origin (0,0) were represented. A correlation between the first factor scores and the marginal genotypic BLUPs was r^2^ = 0.897 in the Bayesian FA model and r^2^ = 0.929 for the FA mixed model, which would also justify the GGE biplot interpretation.

Moreover, for the real data scenario, all factor loadings related to environments were positive and with high overlapping for the credibility intervals (Fig 5B). In this same Figure, it is observed that environments with low residual variances (Table 2) show more concentrated credible regions with respect to those with higher residual variances. Thus, the elliptical range depends on each specific experimental variance. Estimates for factor loadings and factor scores can be found in S3 and S4 Tables.

**Fig 6:**
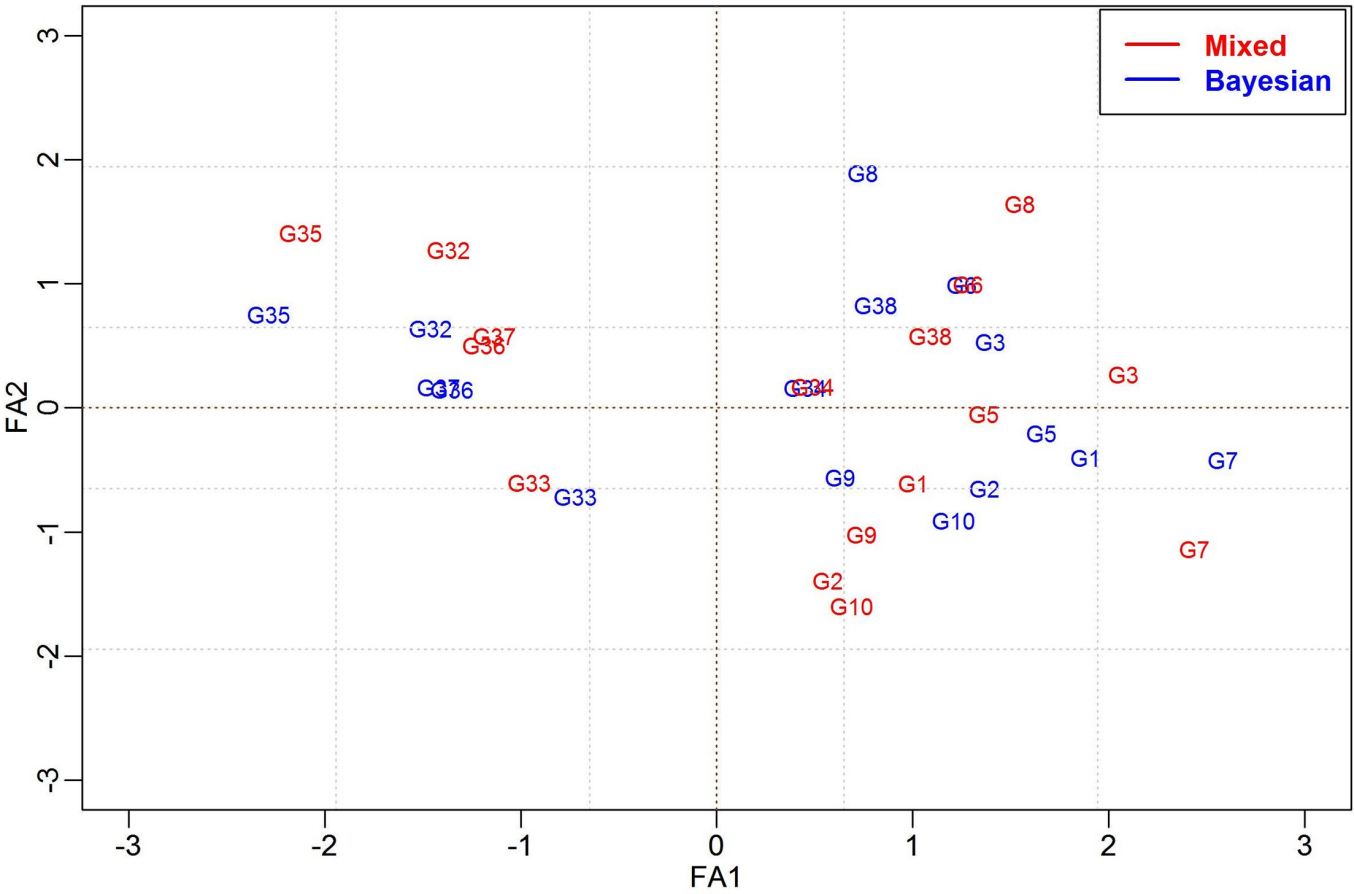
Biplot analysis of genotype scores using the FA-AI model (Red) and the BAF (Blue), considering 50 genotypes evaluated in 10 environments for real data.

**Fig 7:**
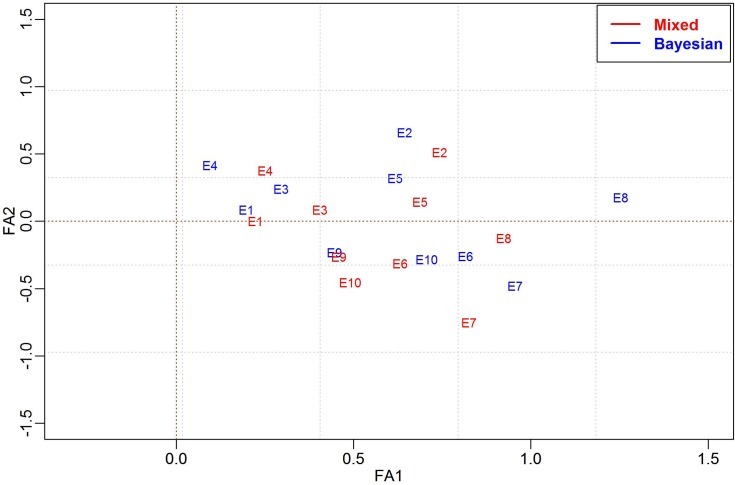
Biplot analysis of environmental scores using the FA-AI model (Red) and the BAF (Blue), considering 50 genotypes evaluated in 10 environments for real data.

Figs 6 and 7 show a brief comparison of the biplot results obtained from the mixed model FA(2) method and our BFA model. Both methods produced similar patterns in the biplots separating the genotypes {G32, G35, G36, G37} from the genotypes {G1, G2, G3, G5, G6, G7, G8, G10, G38}, where each group was clustered in opposite biplot quadrants.

In these same Figures, it was verified that the estimates of the two models were coincident for factor scores and factor loadings. Similarities can be observed when comparing the distribution of environmental scores in standard biplot analyses (Fig 6) compared to those obtained from the Bayesian model. It is possible to verify the change in the E8 position from the FA-mixed model (fourth quadrant) to the Bayesian FA (second quadrant) that may result in small differences in the mega-environments formation and the specific adaptability.

#### Model selection

In the Bayesian factors analysis, one of the most important issues to be addressed is the choice of the appropriate number of factors to be retained in the model.

In Table 3, we present the log-likelihood AICM values of the ten competing models for the real data set. It was verified that the AICM criterion was unable to select the best FA model since the AICM were practically equal.

**Table 3:** AICM values and ΔAICM (difference between the AICM of the complete model and the others) for the selection of FAk models, (k = 1, …, 10) and the ranking of models for real data.

Model	AICM	Rank	ΔAICM	Rank
FA1	-4.351	1^st^-FA10	-0.007	1^st^ -FA2
FA2	-4.347	2^nd^ -FA2	-0.003	2^nd^ FA4
FA3	-4.351	3^rd^- FA4	-0.007	3^rd^ -FA1
FA4	-4.35	4^th^ -FA1	-0.006	4° -FA3
FA5	-4.351	5^th^ -FA3	-0.007	5^th^ -FA5
FA6	-4.351	6^th^-FA5	-0.007	6^th^ -FA6
FA7	-4.354	7^th^ -FA6	-0.01	7^th^ -FA9
FA8	-4.354	8^th^-FA9	-0.01	8^th^ -FA7
FA9	-4.353	9^th^-FA7	-0.009	9^th^ -FA8
FA10	-4.344	10^th^ -FA8	-	-

In this same table, it is possible to verify the ΔAICM for each model. Similarly, there is not a fair criterion to select the number of factor loadings using this information. The results presented in Table 3 indicate the difficulty of selecting the best model since the criterion differentiation occurs only in the third decimal place, making it necessary to add an additional measure for model selection.

In FA models, the genetic covariance is estimated by [**Σ** = (**ΓΓ**^T^)^*k*−1^+**Ψ**]. To ensure the FA model’s identifiability, the loading matrix must be conditioned to the following equality (**ΓΓ**^T^ = **Σ**−**Ψ**).

**Fig 8:**
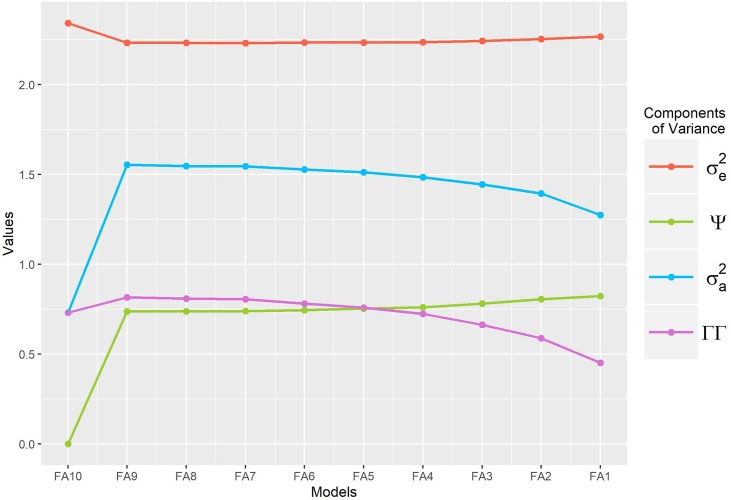
Residual variance ($\sigma_{e}^{2}$), specific (ψ) variance, loading matrix (**ΓΓ**) and recovered genetic variance $\left[\sigma_{g}^{2}=\sqrt[{10}]{\left(diag\left|{\left(\Gamma \Gamma^{T}\right)}^{k-1}+\Psi \right|\right)}\right]$ on different FA(k) structures for real data.

In Fig 8, we can verify that when the full model-FA *k* is adjusted, the proportion of marginal genetic variance given by the geometric mean $\left[\sigma_{g}^{2}=\sqrt[{10}]{\left(diag\left|\Sigma \right|\right)}\right]$ is fully recovered by the loadings $\left[\sqrt[{10}]{diag\left(\Gamma \Gamma^{T}\right)}\right]$ with a geometric mean of the residual variance given by $\left[\sigma_{e}^{2}=\sqrt[{10}]{\left|R\right|}\right]$. This situation corresponds to an unstructured model. When the one axis is removed from the full model, one can verify the decreasing residual variance and the fast increase of the specific variance that merges the genetic and residual (noise) variances.

It can be verified that starting from FA *(k-1)* model to model FA1, the variance $\sigma_{e}^{2}$ remains constant, but the harmonic mean related to the specific variance $\left[\psi =\sqrt[{10}]{diag\left|\Psi \right|}\right]$ increases as the FA model becomes more parsimonious. In addition, (as expected) the genotypic variance recovered by the loadings $\sqrt[{10}]{diag\left(\Gamma \Gamma^{T}\right)}$ decreases. It is possible to note that the use of complex models (high *k*-dimensional FA) has no advantage or gain in the calculation of $\sigma_{g}^{2}$ or $\sigma_{e}^{2}$. Thus, under the identifiability imposed in this study, the performance of the model selection tests using the likelihood can be complicated, since the specific variances (or noise) are estimated separately from the experimental error and the missing variance unrecovered by the loading in low-dimension FA structure recovered by the specific variances given in **Ψ**.

## Discussion

The development of models able to describe the response of genotypes in environmental networks has become a great challenge to quantitative breeders, mainly in the genome selection context [46] The use of factorial analytical structures in MET analyses has contributed greatly to the analysis and meta-analysis of phenotypic data in trial networks, presenting unbalanced data and different experimental accuracies. The main difference between the BFA method and the classical mixed model FA lies in the BFA model assumptions that are founded on factor analysis via spectral decomposition of the genetic covariance matrix. This approach allows one to incorporate inference in the biplot of the loadings and rotationally, which is not directly performed in classical FA analysis.

This particularity ensures the model’s identifiability and avoids the occurrence of estimates outside of the parametric space (Heywood cases), as observed in Smith, Cullis and Thompson [6]. In addition, it eliminates the need for loadings’ rotationality, providing robust estimates for covariance parameters through loadings and factor scores. Thus, the BFA method guarantees the ability to test all latent factors that cannot be ensured by classical FA model. For example, it was observed that the FA analysis conducted in Asreml-R on real data did not converge on more complex FA models (FA≥3) and a fair scanning for the best model was not possible using the real data. The selection of the number of *k* factors is still a non-trivial issue and inadequate choices can result in biased and unstable estimates of **Σ** and **R** [47,48].

In this study, we assumed independence among environments assigning the scaled-inverse chi-squared distributions for each diagonal element of the residual variance. However, if this assumption is relaxed, we can use an inverse Wishart prior for the residual covariance matrix (as in de Los Campos and Gianola [25]) or the sparse prior matrix for the loading matrices (as proposed by Runcie and Mukherjee [48]).

It is important to highlight that some problems related to improper posterior may emerge in hierarchical models when non informative priors are used. This issue was discussed in Hobert and Casella [49] and Gelman [50]. While it is not ease checking all marginal posterior in complex models some tips can be observed during MCMC sampling such as non convergence or bimodal posterior presenting high mass on zero value.

In this study, we did not observed such problems as those verified in our previous work using Bayesian hierarchical AMMI model [20]. On the scenario presented by these authors, some *ad hoc* procedures were applied to obtain proper posteriors in family of inverted Gamma-Gaussian hierarchical models when improper Jeffrey’s prior replaces the inverted Gamma distribution. Silva et al. [20] proposed the correction of degree of freedom based on Ter Braak [51] approach and suggested such correction when there is evidence of improper posterior.

The rotationally issue is not discussed in the study of de Los Campos and Gianola [25]. Runcie and Mukherjee [48] argue that the rotationality may be guaranteed by imposing constraints on the loading matrixin the prior specification (hierarchical modeling). As it is known, the rotationality of the loading matrix does not influence the estimation of model parameters. However, it may blur some biological interpretations [6] and hamper the MCMC’s convergence [52].

Moreover, the parametric advantages of the BFA model in its efficiency in representing the FA pattern can be seen in Figs 6 and 7, where the BFA is compared with the FA-AI. The same FA pattern was expected, given the BFA’spriors based on the FA model’s assumptions reported in Smith et al. [6]. Furthermore, in the study involving the simulated data, the estimates obtained for residual variances were close to the true values and the recovery of the first two factor loadings by the eigenvalues and eigenvectors (Table 1).

Other proposals for multi-environment data analysis can be found in the literature; some highlight the use of Bayesian AMMI or SREG models [17–19,23,34]. One limitation of these approaches with respect to BAF is to assume homogeneity of variances and other model assumptions based on ANOVA.

When the homogeneity of variances is assumed, the sampling of the eigenvectors is performed in hyperspherical space through the von Mises-Fisher distributions, as proposed by Liu [52] for the AMMI models. Here, given that different residual variances were assumed across the environments, it was not possible to approximate a von Mises-Fisher distribution as the posterior distribution; rather, the eigenvectors are sampled from a multivariate Gaussian distribution that provides hyper-ellipses instead of hyper-sphere over multivariate spaces. The eigenvectors were further placed in the correct subspace by orthogonal transformation, thus meeting the restrictions imposed by spectral decomposition.

Although the aim of our study is not to provide an intensive comparison of the predictive ability between the BAF and FA-based mixed models, the results showed that even using different likelihood approaches for FA mixed models (AI and EM), the BFA outperformed these models in most of the missing data scenarios (S1–S6 Figs). Given the intra-class correlation (or the average heritability) obtained for the simulated data, one could expect a maximum correlation between the observed and predicted values of 0.82; however, this threshold was exceeded in some folds **(**folds 3 and 5 in S1 Fig), showing that the model’s accuracy may be higher than expected in some scenarios, especially when the group to be predicted is composed of stable genotypes.

The BFA was not superior in all *k*-fold scenarios. For instance, in some *k-*folds, we can verify a marginal loss of BFA with respect to FA-EM. This result was observed under low levels of missing data (10%). Additionally, the predictive ability of the model was similar for some folds, which were 33%, and 50% of data were missing. This result suggests that BFA might be inferior to FA-based mixed models under some data scenarios. As emphasized by Wolpert and Macready [53], the high performance of some algorithms in a class of problems is compensated by the low performance in another class (No Free Lunch Theorem).

The main drawback of the Bayesian approach is the high demand for computational studies of for the MCMC’s convergence. However, this disadvantage can be offset by the greater model flexibility and guaranteed convergence in the parameter space. However, high-technology computers associated with optimized codes and parallel processing can alleviate this disadvantage of MCMCs.

The FA model proposed by Smith et al. [6] and improved by Thompson et al. [54] uses one-stage analysis. Meyer [16] describes this same model using a factor analysis approach that was implemented using a two-stage FA analysis (FA-EM) by Nuvunga et al. [40]. In this study, it was observed that the FA-EM was slightly superior to the classic FA. The relative gain of the FA-EM with respect to the FA-AI can be explained by the structure of the simulated data. According to Nuvunga et al. [40], the FA-EM is estimated in two stages. In the first stage, an unstructured covariance matrix (UN) is estimated by EM. In the second stage, the factor analysis is used to estimate the loading and FA scores using the varimax rotationally. In turn, the FA-AI estimates the covariance matrix approximating the FA loadings and scores using a single-stage analysis. Since few environments were simulated and given that the FA-EM is based on previous UN analysis (which tends to be the best choice for low-dimensional data), under this scenario, the FA based on two-stage analysis may have advantages for low-dimensional FA.

Crossa [55] note that the FA model can be interpreted in a similar way to the SREG model when the G effect is confounded with GEI or as in the AMMI model if the G effects are marginalized from GEI. Burgueño et al. [56] notes that there is no clear difference in the predictions between these two approaches. However, it should be noted that the FA models using the confounding of G+GE (as presented here) are more parsimonious than those FA models that marginalize the genotype effect from the GEI.

The bivariate credibility regions (at 95% probability) were incorporated into the FA biplots (Figs 1A, 1B, 5A and 5B), identifying homogeneous subgroups of genotypes and environments for adaptability and stability. Through the credibility regions, it is possible to identify which environments show greater variability or contribute to GEI. The interpretation of the BFA biplot is similar to that in classical factor analysis, although the uncertainty is considered in the BFA biplot. The joint decomposition of G+GE becomes the model’s interpretation, similar to the GGE-biplot. However, it is noteworthy to highlight that this view must be assumed with caution, since the graphical representation must be performed separately for genotypes and environments, since their responses do not have the same scale or inner-product proprieties.

The selection of models for simulated data was performed by three complementary criteria: the PRESS criterion, the correlation between the observed and the predicted phenotypic value, and the statistical efficiency (SE). It was observed that the model chosen from the simulated data was FA(4). For the real data, we adopted a parametric criterion to select the best model, including the AICM and the ΔAICM information criteria [43]. As observed, the AICM was not informative enough to select the number of factors to be retained in the model, although it presented a tendency using the full model, which in theory recovers the UN matrix. The widely used FA(2) model did not present the best information criterion, even with it being very parsimonious, presenting graphical justifications and genetic interpretations [26,29,40]. The use of information criteria as a selection method for the number of factors has several ambiguities and is always subject to criticism since different criteria tend to select different models, as shown in Table 3 and in Smith et al. [28]. In addition, it can also be observed that the differentiation of models was small, which could be interpreted as a non-selection of models (Table 3).

In addition to information criteria, Smith et al. [28] emphasized the need to use additional measures in the model selection, such as the proportion of variance explained by the components. It was observed that as FA(*k-*1*)* model is adjusted, the residual variance decreases and the genetic variance increases, which is now explained by the loadings and specific variance (Fig 8). From the FA(*k*-2), (*k*-3), …, FA(1) model, it was verified that the specific and residual variances remain constant, showing that the application of information criteria as a model selection method is not easy t under the restrictions imposed in the BFA analysis.

Some other information criterion based on expectation of likelihood could be applied such us the averaged BIC, DIC, AEBIC and so on [57]. While these methods may present divergent behavior of AICM and the ΔAICM information criteria, we understand that, on the present scenario, the best Bayesian FA model selected by these information criterions could differ qualitatively from AICM (i.e. selecting the FA(1) model) but presenting low quantitative differences since the average marginal likelihood will be the same and the penalty criterion could not be large enough to efficiently separate the FA models within each information criterion. It worth to highlight that this occurrence is not a problem related to the information criterions, but a characteristic of our model since the restriction (**ΓΓ**^T^ = **Σ**−**Ψ**) ensure equivalence among the marginal likelihoods across the FA models.

In general, the choice of the FA2 model has become consensual among researchers. It is argued that increasing the number of components (such as FA3, FA4 and FA5) does not guarantee better prediction ability, but it will certainly increase the model’s complexity; therefore, it is doubtful that a better adjustment will be produced, as observed in our study with real and simulated data. The results obtained by Burgueño et al. [26] Burgueño et al. [7], Kelly et al. [9] and Nuvunga et al. [40] show that FA models with more than two components improved variance-covariance estimates, but this was not reflected in the genotypic predicted values (EBLUPs).

In this study, it was not our intention to give a fine perspective of model selection in FA bayesian framework; instead, our aim was to provide a bayesian perspective of FA models in MET analysis. While the model selection is an open issue in FA models, the cross-validation approaches used here, in general, were more informative to select FA scores than AIC criterion. Others methods based on transdimentional models such as reversible jump could be proposed in order to select FA scores [58]; but, until now, results in this area are scarce in Bayesian context.

Although both data sets used in this study may be relatively small when compared to the data available from experimental trials in breeding programs, it is sufficient to show the strength of the BFA method in MET analysis, presenting a better predictive ability than the classical FA based on mixed models. Given that in the MET analysis some environments are not present in the trial network and further years are unpredictable, some functional information may be included in the FA analysis to predict the genotypic values for coming years; for example, we can take the covariance matrix as a functional response surface related to some distance measures in the Hilbert space.

Our results demonstrate that the Bayesian FA model can be effectively implemented to study GEI patterns in MET networks and predict missing data with high levels of imbalance.

## Supporting information

S1 Text Justification of bayesian factor analytical model. (DOCX)

S2 Text Likelihood justification. (DOCX)

S3 Text Gibbs sampler algorithm. (DOCX)

S4 Text Traces and densities for the MCMC chains for some parameters of model. (DOCX)

S1 Table Description of the environments where the experiments were conducted. (DOCX)

S2 Table Posterior means (PM), regions of credibility (95%. LL: Lower limit. UL: Upper limit) and estimates of restricted maximum likelihood (REML) of FA-AI and genotypic scores (f_*i*1_−f_*i*2_), simulated data. (DOCX)

S3 Table Posterior means (PM), regions of credibility (95%. LL: Lower limit. UL: Upper limit) for the first two factor loadings (*λ*_1_α_1_− *λ*_2_α_2_), real data. (DOCX)

S4 Table Posterior means (PM), regions of credibility (95%. LL: Lower limit. UL: Upper limit) for the first two factor scores (f_*i*1_−f_*i*2_), real data. (DOCX)

S1 Fig Bar chart representing the correlation for the 10% loss level using the Bayesian FA (BAF) and FA (FA-EM and FA-AI) models for simulated data. (JPEG)

S2 Fig Bar chart representing the correlation for the 33% loss level using the Bayesian FA (BAF) and FA (FA-EM and FA-AI) models for simulated data. (JPEG)

S3 Fig Bar chart representing the correlation for the 50% loss level using the Bayesian FA (BAF) and FA (FA-EM and FA-AI) models for simulated data. (JPEG)

S4 Fig Bar chart representing the PRESS for the 10% loss level using the Bayesian FA (BAF) and FA (FA-EM and FA-AI) models for simulated data. (JPEG)

S5 Fig Bar chart representing the PRESS for the 33% loss level using the Bayesian FA (BAF) and FA (FA-EM and FA-AI) models for simulated data. (JPEG)

S6 Fig Bar chart representing the PRESS for the 50% loss level using the Bayesian FA (BAF) and FA (FA-EM and FA-AI) models for simulated data. (JPEG)

S1 Data Simulated data. (RAR)

S2 Data Real data. (RAR)

S3 Data R BFA package. (ZIP)
