# Supplementary material for: Bayesian factor analytic model: An approach in multiple environment trials
Source: PLoS One. 2019 Aug 22;14(8):e0220290. doi: 10.1371/journal.pone.0220290 (PMC6705866; doi:10.1371/journal.pone.0220290)
Supplement: S1 Text — (DOCX) [file pone.0220290.s001.docx]

# S1 Text

For illustration, with no loss of generality, considering the model for three genotypes (p = 3) and two environments (m = 2) with two replicates (r = 2).

$$\begin{matrix} \begin{matrix} & & \mathbf{G1} & & & \mathbf{G2} & & & \mathbf{G3} \end{matrix} \\ \begin{matrix} \mathbf{A1} \\ \mathbf{A2} \end{matrix}\left[\begin{matrix} y_{111} & y_{112} & y_{211} & y_{212} & y_{311} & y_{312} \\ y_{121} & y_{121} & y_{221} & y_{222} & y_{321} & y_{322} \end{matrix}\right] \end{matrix}$$

Using the standard multivariate mixed model,

$\mathbf{y=X\beta+Zu+\varepsilon}$ (1)

the system would be solved as follows:

Based on the model proposed by Smith et al. (2001), we have:$\mathbf{Zu}=\left[\mathbf{Z}_{\mathbf{1}}\mathbf{\ldots Z}_{m}\right]\left[\begin{matrix} u_{1} \\ \vdots\\ u_{m} \end{matrix}\right]=\mathbf{Z}_{\mathbf{1}}u_{1}+\cdots+\mathbf{Z}_{m}u_{m}$, em que $\mathbf{u=(\Gamma}_{\text{m}}\otimes\mathbf{I}_{\text{p}}\mathbf{)f+\delta}$.

So, the model (1) would be rewritten as:

$\mathbf{y=X\beta+Z(\Gamma}_{\text{m}}\otimes\mathbf{I}_{\text{p}}\mathbf{)f+Z\delta+\varepsilon}$ (2)

Describing only the terms $\mathbf{Zu=Z(\Gamma}_{\text{m}}\otimes\mathbf{I}_{\text{p}}\mathbf{)f+Z\delta}$ for a complete FA model $\mathbf{\delta}=\mathbf{0}$.

In (2), $\mathbf{I}_{\text{p}}$ is a diagonal matrix of dimension (*p* x *p*); $\mathbf{Z}$ is the design matrix of the random effects of dimension (12 x 6), and the terms $\mathbf{\Gamma}_{\text{m}}$and $\mathbf{f}$ denote the matrix of the factorial loads and the vector with the factorial scores, respectively, as represented below:

$\mathbf{\Gamma}_{\text{m}}=\left[\begin{matrix} \tau_{11} & \tau_{12} \\ \tau_{21} & \tau_{22} \end{matrix}\right]$ e $\mathbf{f}^{\top}=\left[\begin{matrix} f_{11} & f_{12} & f_{13} & f_{21} & f_{22} & f_{23} \end{matrix}\right]_{1\times6}^{\top}$

Solving the system $\mathbf{Z(\Gamma}_{\text{m}}\otimes\mathbf{I}_{\text{p}}\mathbf{)f}$:

i)

ii)

Thus, the system can be described as:

iii) $\left[\begin{matrix} \begin{matrix} y_{111} \\ \begin{matrix} y_{112} \\ y_{211} \\ \begin{matrix} y_{212} \\ y_{311} \\ y_{312} \end{matrix} \end{matrix} \\ y_{121} \\ y_{122} \end{matrix} \\ y_{221} \\ y_{222} \\ \begin{matrix} y_{321} \\ y_{322} \end{matrix} \end{matrix}\right]_{12\times1}=\left[\begin{matrix} \begin{matrix} 1 \\ 1 \\ 1 \\ \begin{matrix} 1 \\ 1 \\ 1 \\ \begin{matrix} 1 \\ 1 \\ 1 \\ \begin{matrix} 1 \\ 1 \\ 1 \end{matrix} \end{matrix} \end{matrix} \end{matrix} & \begin{matrix} 1 \\ 1 \\ 1 \\ \begin{matrix} 1 \\ 1 \\ 1 \\ \begin{matrix} 0 \\ 0 \\ 0 \\ \begin{matrix} 0 \\ 0 \\ 0 \end{matrix} \end{matrix} \end{matrix} \end{matrix} & \begin{matrix} 0 \\ 0 \\ 0 \\ \begin{matrix} 0 \\ 0 \\ 0 \\ \begin{matrix} 1 \\ 1 \\ 1 \\ \begin{matrix} 1 \\ 1 \\ 1 \end{matrix} \end{matrix} \end{matrix} \end{matrix} \end{matrix}\right]_{12\times3}\left[\begin{matrix} \mu\\ \beta_{1} \\ \beta_{2} \end{matrix}\right]_{3\times1}+\left[\begin{matrix} \tau_{11}f_{11}+\tau_{12}f_{21} \\ \tau_{11}f_{11}+\tau_{12}f_{21} \\ \tau_{11}f_{12}+\tau_{12}f_{22} \\ \tau_{11}f_{12}+\tau_{12}f_{22} \\ \tau_{11}f_{13}+\tau_{12}f_{23} \\ \tau_{11}f_{13}+\tau_{12}f_{23} \\ \tau_{21}f_{11}+\tau_{22}f_{21} \\ \tau_{21}f_{11}+\tau_{22}f_{21} \\ \tau_{21}f_{12}+\tau_{22}f_{22} \\ \tau_{21}f_{12}+\tau_{22}f_{22} \\ \tau_{21}f_{13}+\tau_{22}f_{23} \\ \tau_{21}f_{13}+\tau_{22}f_{23} \end{matrix}\right]_{12\times1}+\left[\begin{matrix} \begin{matrix} \varepsilon_{111} \\ \begin{matrix} \varepsilon_{112} \\ \varepsilon_{211} \\ \begin{matrix} \varepsilon_{212} \\ \varepsilon_{311} \\ \varepsilon_{312} \end{matrix} \end{matrix} \\ \varepsilon_{121} \\ \varepsilon_{122} \end{matrix} \\ \varepsilon_{221} \\ \varepsilon_{222} \\ \begin{matrix} \varepsilon_{321} \\ \varepsilon_{322} \end{matrix} \end{matrix}\right]_{12\times1}$

The model (2) can be reparametrized considering a structure based on spectral decomposition. In this approach, the matrix of factor loads is obtained by $\hat{\mathbf{\Gamma}}_{k}=\mathbf{V}\Lambda^{\frac{1}{2}}$, in which $\mathbf{\Lambda}$ is the diagonal matrix composed of eigenvalues $\mathbf{\Lambda}=diag(\lambda_{1},...\lambda_{k})$ subject to restriction of $\lambda_{k}\geq\lambda_{k+1}$ and $\mathbf{V}$is the matrix of singular vectors, subject to restriction of orthonormal.

So we have:

$$\mathbf{Z(\Gamma}_{\text{m}}\otimes\mathbf{I}_{\text{p}}\mathbf{)f}=\sum_{k=1}^{m} \lambda_{k}diag\left(\mathbf{X}_{\mathbf{2}}\mathbf{\alpha}_{k}\right)\mathbf{Z}_{f}\mathbf{f}_{\mathbf{k}}$$

In this way, it is possible to rewrite the model (2) as:

$\mathbf{y=X}_{\mathbf{1}}\mathbf{\beta+}\sum_{k=1}^{m} \lambda_{k}diag\mathbf{(X}_{\mathbf{2}}\mathbf{\alpha}_{\mathbf{k}}\mathbf{)Z}_{f}\mathbf{f}_{k}\mathbf{+Z\delta+\varepsilon}$ (3)

Considering the fit of the complete model ($\mathbf{Z\delta}=\mathbf{0}$) and solving only the term $\sum_{k=1}^{m} \lambda_{k}diag\mathbf{(X}_{\mathbf{2}}\mathbf{\alpha}_{k}\mathbf{)Z}_{f}\mathbf{f}_{k}$ we have the following expression:

$$\sum_{k=1}^{2} \lambda_{k}diag\mathbf{(X}_{\mathbf{2}}\mathbf{\alpha}_{k}\mathbf{)Z}_{f}\mathbf{f}_{\mathbf{k}}=\lambda_{1}diag\mathbf{(X}_{\mathbf{2}}\mathbf{\alpha}_{1}\mathbf{)Z}_{f}\mathbf{f}_{1}+\lambda_{2}diag\mathbf{(X}_{\mathbf{2}}\mathbf{\alpha}_{2}\mathbf{)Z}_{f}\mathbf{f}_{2}$$

that in matrix notation is represented by the linear system below:

$$\lambda_{1}diag\left(\left[\begin{matrix} 1 & 0 \\ 1 & 0 \\ 1 & 0 \\ 1 & 0 \\ 1 & 0 \\ 1 & 0 \\ 0 & 1 \\ \begin{matrix} 0 \\ 0 \\ 0 \\ 0 \\ 0 \end{matrix} & \begin{matrix} 1 \\ 1 \\ 1 \\ 1 \\ 1 \end{matrix} \end{matrix}\right]\left[\begin{matrix} \alpha_{11} \\ \alpha_{21} \end{matrix}\right]\right)\left[\begin{matrix} 1 & 0 & 0 \\ 1 & 0 & 0 \\ 0 & 1 & 0 \\ 0 & 1 & 0 \\ 0 & 0 & 1 \\ 0 & 0 & 1 \\ 1 & 0 & 0 \\ \begin{matrix} 1 \\ 0 \\ 0 \\ 0 \\ 0 \end{matrix} & \begin{matrix} 0 \\ 1 \\ 1 \\ 0 \\ 0 \end{matrix} & \begin{matrix} 0 \\ 0 \\ 0 \\ 1 \\ 1 \end{matrix} \end{matrix}\right]\left[\begin{matrix} f_{11} \\ \begin{matrix} f_{12} \\ f_{13} \end{matrix} \end{matrix}\right]+\lambda_{2}diag\left(\left[\begin{matrix} 1 & 0 \\ 1 & 0 \\ 1 & 0 \\ 1 & 0 \\ 1 & 0 \\ 1 & 0 \\ 0 & 1 \\ \begin{matrix} 0 \\ 0 \\ 0 \\ 0 \\ 0 \end{matrix} & \begin{matrix} 1 \\ 1 \\ 1 \\ 1 \\ 1 \end{matrix} \end{matrix}\right]\left[\begin{matrix} \alpha_{12} \\ \alpha_{22} \end{matrix}\right]\right)\left[\begin{matrix} 1 & 0 & 0 \\ 1 & 0 & 0 \\ 0 & 1 & 0 \\ 0 & 1 & 0 \\ 0 & 0 & 1 \\ 0 & 0 & 1 \\ 1 & 0 & 0 \\ \begin{matrix} 1 \\ 0 \\ 0 \\ 0 \\ 0 \end{matrix} & \begin{matrix} 0 \\ 1 \\ 1 \\ 0 \\ 0 \end{matrix} & \begin{matrix} 0 \\ 0 \\ 0 \\ 1 \\ 1 \end{matrix} \end{matrix}\right]\left[\begin{matrix} f_{21} \\ \begin{matrix} f_{22} \\ f_{23} \end{matrix} \end{matrix}\right]$$

So $\lambda_{1}diag\mathbf{(X}_{\mathbf{2}}\mathbf{\alpha}_{1}\mathbf{)Z}_{f}\mathbf{f}_{1}+\lambda_{2}diag\mathbf{(X}_{\mathbf{2}}\mathbf{\alpha}_{2}\mathbf{)Z}_{f}\mathbf{f}_{2}$, in which:

$\lambda_{1}diag\mathbf{(X}_{\mathbf{2}}\mathbf{\alpha}_{\mathbf{1}}\mathbf{)Z}_{f}\mathbf{f}_{\mathbf{1}}=\left[\begin{matrix} \lambda_{1}\alpha_{11}f_{11} \\ \lambda_{1}\alpha_{11}f_{11} \\ \lambda_{1}\alpha_{11}f_{12} \\ \lambda_{1}\alpha_{11}f_{12} \\ \lambda_{1}\alpha_{11}f_{13} \\ \lambda_{1}\alpha_{11}f_{13} \\ \lambda_{1}\alpha_{21}f_{11} \\ \lambda_{1}\alpha_{21}f_{11} \\ \lambda_{1}\alpha_{21}f_{12} \\ \lambda_{1}\alpha_{21}f_{12} \\ \lambda_{1}\alpha_{21}f_{13} \\ \lambda_{1}\alpha_{21}f_{13} \end{matrix}\right]$ and $\lambda_{2}diag\mathbf{(X}_{\mathbf{2}}\mathbf{\alpha}_{2}\mathbf{)Z}_{f}\mathbf{f}_{2}=\left[\begin{matrix} \lambda_{2}\alpha_{12}f_{21} \\ \lambda_{2}\alpha_{12}f_{21} \\ \lambda_{2}\alpha_{12}f_{22} \\ \lambda_{2}\alpha_{12}f_{22} \\ \lambda_{2}\alpha_{12}f_{23} \\ \lambda_{2}\alpha_{12}f_{23} \\ \lambda_{2}\alpha_{22}f_{21} \\ \lambda_{2}\alpha_{22}f_{21} \\ \lambda_{2}\alpha_{22}f_{22} \\ \lambda_{2}\alpha_{22}f_{22} \\ \lambda_{2}\alpha_{22}f_{23} \\ \lambda_{2}\alpha_{22}f_{23} \end{matrix}\right]$

And the system has as solution:

$$=\left[\begin{matrix} \lambda_{1}\alpha_{11}f_{11} \\ \lambda_{1}\alpha_{11}f_{11} \\ \lambda_{1}\alpha_{11}f_{12} \\ \lambda_{1}\alpha_{11}f_{12} \\ \lambda_{1}\alpha_{11}f_{13} \\ \lambda_{1}\alpha_{11}f_{13} \\ \lambda_{1}\alpha_{21}f_{11} \\ \lambda_{1}\alpha_{21}f_{11} \\ \lambda_{1}\alpha_{21}f_{12} \\ \lambda_{1}\alpha_{21}f_{12} \\ \lambda_{1}\alpha_{21}f_{13} \\ \lambda_{1}\alpha_{21}f_{13} \end{matrix}\right]+\left[\begin{matrix} \lambda_{2}\alpha_{12}f_{21} \\ \lambda_{2}\alpha_{12}f_{21} \\ \lambda_{2}\alpha_{12}f_{22} \\ \lambda_{2}\alpha_{12}f_{22} \\ \lambda_{2}\alpha_{12}f_{23} \\ \lambda_{2}\alpha_{12}f_{23} \\ \lambda_{2}\alpha_{22}f_{21} \\ \lambda_{2}\alpha_{22}f_{21} \\ \lambda_{2}\alpha_{22}f_{22} \\ \lambda_{2}\alpha_{22}f_{22} \\ \lambda_{2}\alpha_{22}f_{23} \\ \lambda_{2}\alpha_{22}f_{23} \end{matrix}\right]=\left[\begin{matrix} \lambda_{1}\alpha_{11}f_{11}+\lambda_{2}\alpha_{12}f_{21} \\ \lambda_{1}\alpha_{11}f_{11}+\lambda_{2}\alpha_{12}f_{21} \\ \lambda_{1}\alpha_{11}f_{12}+\lambda_{2}\alpha_{12}f_{22} \\ \lambda_{1}\alpha_{11}f_{12}+\lambda_{2}\alpha_{12}f_{22} \\ \lambda_{1}\alpha_{11}f_{13}+\lambda_{2}\alpha_{12}f_{23} \\ \lambda_{1}\alpha_{11}f_{13}+\lambda_{2}\alpha_{12}f_{23} \\ \lambda_{1}\alpha_{21}f_{11}+\lambda_{2}\alpha_{22}f_{21} \\ \lambda_{1}\alpha_{21}f_{11}+\lambda_{2}\alpha_{22}f_{21} \\ \lambda_{1}\alpha_{21}f_{12}+\lambda_{2}\alpha_{22}f_{22} \\ \lambda_{1}\alpha_{21}f_{12}+\lambda_{2}\alpha_{22}f_{22} \\ \lambda_{1}\alpha_{21}f_{13}+\lambda_{2}\alpha_{22}f_{23} \\ \lambda_{1}\alpha_{21}f_{13}+\lambda_{2}\alpha_{22}f_{23} \end{matrix}\right]$$

Therefore, the expansion of the model (2) is given by:

$$\mathbf{y=X}_{\mathbf{1}}\mathbf{\beta+}\sum_{k=1}^{m} \lambda_{k}diag\left(\mathbf{X}_{\mathbf{2}}\mathbf{\alpha}_{k}\right)\mathbf{Z}_{f}\mathbf{f}_{k}\mathbf{+Z\delta+\varepsilon}$$

$$\left[\begin{matrix} \begin{matrix} y_{111} \\ \begin{matrix} y_{112} \\ y_{211} \\ \begin{matrix} y_{212} \\ y_{311} \\ y_{312} \end{matrix} \end{matrix} \\ y_{121} \\ y_{122} \end{matrix} \\ y_{221} \\ y_{222} \\ \begin{matrix} y_{321} \\ y_{322} \end{matrix} \end{matrix}\right]_{12\times1}=\left[\begin{matrix} \begin{matrix} 1 \\ 1 \\ 1 \\ \begin{matrix} 1 \\ 1 \\ 1 \\ \begin{matrix} 1 \\ 1 \\ 1 \\ \begin{matrix} 1 \\ 1 \\ 1 \end{matrix} \end{matrix} \end{matrix} \end{matrix} & \begin{matrix} 1 \\ 1 \\ 1 \\ \begin{matrix} 1 \\ 1 \\ 1 \\ \begin{matrix} 0 \\ 0 \\ 0 \\ \begin{matrix} 0 \\ 0 \\ 0 \end{matrix} \end{matrix} \end{matrix} \end{matrix} & \begin{matrix} 0 \\ 0 \\ 0 \\ \begin{matrix} 0 \\ 0 \\ 0 \\ \begin{matrix} 1 \\ 1 \\ 1 \\ \begin{matrix} 1 \\ 1 \\ 1 \end{matrix} \end{matrix} \end{matrix} \end{matrix} \end{matrix}\right]_{12\times3}\left[\begin{matrix} \mu\\ \beta_{1} \\ \beta_{2} \end{matrix}\right]_{3\times1}+\left[\begin{matrix} \lambda_{1}\alpha_{11}f_{11}+\lambda_{2}\alpha_{12}f_{21} \\ \lambda_{1}\alpha_{11}f_{11}+\lambda_{2}\alpha_{12}f_{21} \\ \lambda_{1}\alpha_{11}f_{12}+\lambda_{2}\alpha_{12}f_{22} \\ \lambda_{1}\alpha_{11}f_{12}+\lambda_{2}\alpha_{12}f_{22} \\ \lambda_{1}\alpha_{11}f_{13}+\lambda_{2}\alpha_{12}f_{23} \\ \lambda_{1}\alpha_{11}f_{13}+\lambda_{2}\alpha_{12}f_{23} \\ \lambda_{1}\alpha_{21}f_{11}+\lambda_{2}\alpha_{22}f_{21} \\ \lambda_{1}\alpha_{21}f_{11}+\lambda_{2}\alpha_{22}f_{21} \\ \lambda_{1}\alpha_{21}f_{12}+\lambda_{2}\alpha_{22}f_{22} \\ \lambda_{1}\alpha_{21}f_{12}+\lambda_{2}\alpha_{22}f_{22} \\ \lambda_{1}\alpha_{21}f_{13}+\lambda_{2}\alpha_{22}f_{23} \\ \lambda_{1}\alpha_{21}f_{13}+\lambda_{2}\alpha_{22}f_{23} \end{matrix}\right]_{12\times1}+\left[\begin{matrix} \begin{matrix} \varepsilon_{111} \\ \begin{matrix} \varepsilon_{112} \\ \varepsilon_{211} \\ \begin{matrix} \varepsilon_{212} \\ \varepsilon_{311} \\ \varepsilon_{312} \end{matrix} \end{matrix} \\ \varepsilon_{121} \\ \varepsilon_{122} \end{matrix} \\ \varepsilon_{221} \\ \varepsilon_{222} \\ \begin{matrix} \varepsilon_{321} \\ \varepsilon_{322} \end{matrix} \end{matrix}\right]_{12\times1}$$

Knowing that $\lambda_{k}\alpha_{kr}=\tau_{kr}$, correspond to the factor loads, as has been shown, the models (1) and (2) are equivalent.

$$\left[\begin{matrix} \lambda_{1}\alpha_{11}f_{11}+\lambda_{2}\alpha_{12}f_{21} \\ \lambda_{1}\alpha_{11}f_{11}+\lambda_{2}\alpha_{12}f_{21} \\ \lambda_{1}\alpha_{11}f_{12}+\lambda_{2}\alpha_{12}f_{22} \\ \lambda_{1}\alpha_{11}f_{12}+\lambda_{2}\alpha_{12}f_{22} \\ \lambda_{1}\alpha_{11}f_{13}+\lambda_{2}\alpha_{12}f_{23} \\ \lambda_{1}\alpha_{11}f_{13}+\lambda_{2}\alpha_{12}f_{23} \\ \lambda_{1}\alpha_{21}f_{11}+\lambda_{2}\alpha_{22}f_{21} \\ \lambda_{1}\alpha_{21}f_{11}+\lambda_{2}\alpha_{22}f_{21} \\ \lambda_{1}\alpha_{21}f_{12}+\lambda_{2}\alpha_{22}f_{22} \\ \lambda_{1}\alpha_{21}f_{12}+\lambda_{2}\alpha_{22}f_{22} \\ \lambda_{1}\alpha_{21}f_{13}+\lambda_{2}\alpha_{22}f_{23} \\ \lambda_{1}\alpha_{21}f_{13}+\lambda_{2}\alpha_{22}f_{23} \end{matrix}\right]_{12\times1}=\left[\begin{matrix} \tau_{11}f_{11}+\tau_{12}f_{21} \\ \tau_{11}f_{11}+\tau_{12}f_{21} \\ \tau_{11}f_{12}+\tau_{12}f_{22} \\ \tau_{11}f_{12}+\tau_{12}f_{22} \\ \tau_{11}f_{13}+\tau_{12}f_{23} \\ \tau_{11}f_{13}+\tau_{12}f_{23} \\ \tau_{21}f_{11}+\tau_{22}f_{21} \\ \tau_{21}f_{11}+\tau_{22}f_{21} \\ \tau_{21}f_{12}+\tau_{22}f_{22} \\ \tau_{21}f_{12}+\tau_{22}f_{22} \\ \tau_{21}f_{13}+\tau_{22}f_{23} \\ \tau_{21}f_{13}+\tau_{22}f_{23} \end{matrix}\right]_{12\times1}$$
