# Supplementary material for: Bayesian factor analytic model: An approach in multiple environment trials
Source: PLoS One. 2019 Aug 22;14(8):e0220290. doi: 10.1371/journal.pone.0220290 (PMC6705866; doi:10.1371/journal.pone.0220290)
Supplement: S2 Text — (DOCX) [file pone.0220290.s002.docx]

# S2 Text

The classical factor analysis is performed using the following steps:

1. Given that the multivariate $\mathbf{y}$the linear model with latent factors is given by:

$$\mathbf{y}=\mu+\mathbf{\Gamma f}+\mathbf{\delta}; Var(\mathbf{y})=\mathbf{\Gamma\Gamma}^{\top}\mathbf{+\Psi}$$

1. The factor model with Gaussian response for the vector $\mathbf{y}$ is given by:

$P(\mathbf{y|\beta,\Gamma,f})\sim N(\mu+\mathbf{\Gamma f,\Psi})$,

In classical factor analysis the latent factors are random variables with $p(\mathbf{f})\sim N(0\mathbf{,I})$, assuming $\mathbf{\theta}=(\mu\mathbf{,} \mathbf{\Gamma})$. The joint probability of the random vectors is given by:

$$P(\mathbf{y,f|\theta})=P(\mathbf{y|f,\theta})P(\mathbf{f})$$

The marginal distribution for $\mathbf{y}$ is given integrating **f** out:

$$P(\mathbf{y|\theta})=\int P(\mathbf{y|f,\theta})P(\mathbf{f|\theta})df$$

$$P(\mathbf{y|\theta})=N\left(\mathbf{\mu},\mathbf{\Gamma\Gamma}^{\top}\mathbf{+\Psi}\right)$$

1. In the classical mixed models in multi-environments trials analysis proposed by (Smith, Cullis and Thompson, 2001) one have:

$$\mathbf{y}=\mathbf{X\beta}+\mathbf{Zu}+\mathbf{e}$$

where $\mathbf{u}$could be described by a factor structure $\mathbf{u=Lf+\delta}$ where:

$$\mathbf{u}\sim N(\mathbf{0},\mathbf{\Gamma\Gamma}^{\top}\mathbf{+\Psi})$$

Using the vector $\mathbf{y}$ and replacing $\mathbf{u=Lf+\delta}$ in the mixed models structure we have

$$\mathbf{y}=\mathbf{X\beta}+\mathbf{Z}[(\mathbf{\Gamma}\otimes\mathbf{I})\mathbf{f}+\mathbf{\delta}]+\mathbf{e}$$

The details about the kronecker product can be obtained in S1-Text in supplemental material. Given that the conditional likelihood is $\mathbf{y}\sim N(\mathbf{X\beta}+\mathbf{Zu},\mathbf{R})$, changing the vector $\mathbf{u}$ by $(\mathbf{\Gamma}\otimes\mathbf{I})\mathbf{f}+\mathbf{\delta}$, we have $L(\mathbf{\theta}|\mathbf{y})=N\left[\mathbf{X\beta}+\mathbf{Z}[(\mathbf{L}\otimes\mathbf{I})\mathbf{f}+\mathbf{\delta}, \mathbf{R}\right]$,

where $\mathbf{\theta}=(\mathbf{\beta,\Gamma,f,\delta})$. Thus, the observed data likelihood is given by: $L(\mathbf{\theta}|\mathbf{y})=N(\mathbf{X\beta},\mathbf{Z}(\mathbf{\Gamma\Gamma+\Psi})\mathbf{Z}+\mathbf{R}]$; where $\mathbf{\theta}$=$(\mathbf{\beta},\mathbf{\Gamma},\mathbf{\delta})$.

Changing the kronecker product $(\mathbf{L}\otimes\mathbf{I})\mathbf{f}$ by $\sum_{k=1}^{p} \lambda_{k}diag\left(\mathbf{X}_{2}\mathbf{\alpha}_{k}\right)\mathbf{Z}_{1}\mathbf{f}_{k}$according to the equations (3), (4), (5) (main text) and the steps described in e S1 Text the conditional likelihood can be described by

$$L\left(\mathbf{\beta,}\lambda\mathbf{,\alpha},\mathbf{f},\mathbf{R|y}\right)\mathbf{\sim N}\left[\mathbf{X}_{1}\mathbf{\beta+}\sum_{k=1}^{p} \lambda_{k}diag\left(\mathbf{X}_{2}\mathbf{\alpha}_{k}\right)\mathbf{Z}_{1}\mathbf{f}_{k}\mathbf{+Z\delta,} \mathbf{R}\right]$$
