# Supplementary material for: Bayesian factor analytic model: An approach in multiple environment trials
Source: PLoS One. 2019 Aug 22;14(8):e0220290. doi: 10.1371/journal.pone.0220290 (PMC6705866; doi:10.1371/journal.pone.0220290)
Supplement: S3 Text — (DOCX) [file pone.0220290.s003.docx]

# S3 Text

#### Gibbs sampler Algorithm

The implemented iterative sampling algorithm can be illustrated by the following steps:

First, the initial guesses are assigned to the model parameters:

$$\Theta_{1}^{0}=\left[\mathbf{\beta}^{0},\mathbf{R}^{0},\mathbf{\lambda}^{0},\mathbf{f}^{0},\mathbf{\alpha}^{0},\mathbf{\delta}^{0},\mathbf{\Psi}^{0}\right]$$

From these initial values, the *i*-th iteration can be obtained as follows:

a) Sample$\mathbf{\beta}^{i}|\mathbf{R}^{i-1},\text{λ}^{i-1},\mathbf{\alpha}^{i-1},\mathbf{f}^{i-1},\mathbf{\delta}^{i-1},\mathbf{\Psi}^{i-1}$from the conditional posterior distribution.

$$\mathbf{\beta}|...\sim N\left[\left(\mathbf{X}_{1}^{\top}\mathbf{R}^{-1}\mathbf{X}_{1}\right)^{-1}\mathbf{X}_{1}^{\top}\mathbf{R}^{-1}\mathbf{A}_{0} ,\left(\mathbf{X}_{1}^{\top}\mathbf{R}^{-1}\mathbf{X}_{1}\right)^{-1}\right]$$

b) Sample$\lambda_{k}^{i}|\mathbf{\beta}^{i},\mathbf{R}^{i},\alpha_{k}^{i-1},f_{k}^{i-1},\mathbf{\delta}^{i-1},\mathbf{\Psi}^{i-1}$from the conditional posterior distribution.

$\text{λ}_{k}|...\propto N^{+}\left[\left(\mathbf{A}_{2k}^{\top}\mathbf{R}^{-1}\mathbf{A}_{2k}\right)^{-1}\mathbf{A}_{2k}^{\top}\mathbf{R}^{-1}\mathbf{A}_{1k},\left(\mathbf{A}_{2k}^{\top}\mathbf{R}^{-1}\mathbf{A}_{2k}\right)^{-1}\right]$,

c) Sample$\mathbf{\alpha}_{k}^{i}|\mathbf{\beta}^{i},\mathbf{R}^{i},\lambda_{k}^{i},f_{k}^{i-1},\mathbf{\delta}^{i-1},\mathbf{\Psi}^{i-1}$ by taking the following steps:

1. Sample$\tilde{\mathbf{\alpha}}_{k}$ from the Gaussian distribution.

$$\tilde{\mathbf{\alpha}}_{k}|...\sim N\left[\mathbf{\alpha}_{k}^{*},\left(\text{C}_{k}^{\top}\text{H}_{k}^{\top}\left(\Delta_{k}^{\top}\mathbf{R}^{-1}\Delta_{k}\right)\text{H}_{k}C_{k}\right)^{-1}\right]$$

1. Apply the transformation $\mathbf{\alpha}_{k}\text{=} \mathbf{H}_{r}\mathbf{\alpha}_{k}^{*}$to obtain the vector in the correct subspace in $\Re^{m}$.

d) Sample$\mathbf{f}|\mathbf{\beta}^{i},\mathbf{R}^{i},\mathbf{\alpha}^{i},\mathbf{\lambda}^{i},\mathbf{\delta}^{i-1},\mathbf{\Psi}^{i-1}$ from the following conditional posterior distribution.

$$\mathbf{f}|...\sim\mathbf{N}\left[\left(\mathbf{I}+\mathbf{A}_{4}^{\top}\mathbf{R}^{-1}\mathbf{A}_{4}\right)^{-1}\mathbf{A}_{4}^{\top}\mathbf{R}^{-1}\mathbf{A}_{5};\left(\mathbf{I}+\mathbf{A}_{4}^{\top}\mathbf{R}^{-1}\mathbf{A}_{4}\right)^{-1}\right]$$

For an FA model where *k*<p (low order FA dimension, the following steps "**e**" and "**f**" are added to the algorithm.

e) Sample$\mathbf{\delta}^{i}|\mathbf{\beta}^{i},\mathbf{R}^{i},\mathbf{\alpha}^{i},\text{λ}^{i},\mathbf{f}^{i},\mathbf{\Psi}^{i-1}$ from the following conditional posterior distribution.

$$\mathbf{\delta}|...\sim N\left[\left(\mathbf{Z}^{\top}\mathbf{V}^{-1}\mathbf{Z}+\mathbf{M}^{-1}\right)^{-1}\mathbf{Z}^{\top}\mathbf{R}^{-1}\mathbf{A}_{7},\left(\mathbf{Z}^{\top}\mathbf{V}^{-1}\mathbf{Z}+\mathbf{M}^{-1}\right)^{-1}\right]$$

f) Sample$\mathbf{\Psi}^{i}|\mathbf{\beta}^{i},\mathbf{R}^{i},\lambda^{i}\mathbf{\alpha}^{i},\mathbf{f}^{i},\mathbf{\delta}^{i}$ from the conditional posterior distribution.

$\psi_{kk}|...\sim Scale-\chi^{-2}\left[m_{k}+\nu_{k},\frac{\left(m{}_{k}(\mathbf{\delta}^{\top}\mathbf{\delta}/m{}_{k})+\nu_{k}S_{k}^{2}\right)}{m_{k}+\nu_{k}}\right]$, for *k = 1, …, p*.

g) Given that **R** is a diagonal matrix, the sampling was performed through the individual elements$\sigma_{e_{k}}^{2}$that were sampled from the following conditional posterior distribution.

$$\sigma_{e_{k}}^{2}|...\sim Scale-\chi^{-2}\left[n_{k},\frac{\left(\mathbf{y}_{k}-\theta_{k}\right)^{\top}\left(\mathbf{y}_{k}-\theta_{k}\right)}{n_{k}}\right]$$

The steps from "a" to "d" and “g” are requested for the full FA model and from "a" to "g" for model (k<p). After concluded the iterative process and checked the chains’ convergence, the samples were considered to have resulted from the marginal densities. The convergence diagnostic was performed using the Raftery and Lewis [35] and Heidelberger and Welch [36] criterion. All inference processes were performed using the R statistical software [37].
