# Supplementary material for: Bayesian factor analytic model: An approach in multiple environment trials
Source: PLoS One. 2019 Aug 22;14(8):e0220290. doi: 10.1371/journal.pone.0220290 (PMC6705866; doi:10.1371/journal.pone.0220290)
Supplement: S4 Text — (DOCX) [file pone.0220290.s004.docx]

**S4 Text**

Next, the figures of the traces and densities for the MCMC chains for the factorial scores ($\mathbf{f}_{1},\mathbf{f}_{2}$) and residual variances ($\sigma_{ek}^{2}$) of the simulated data are presented.


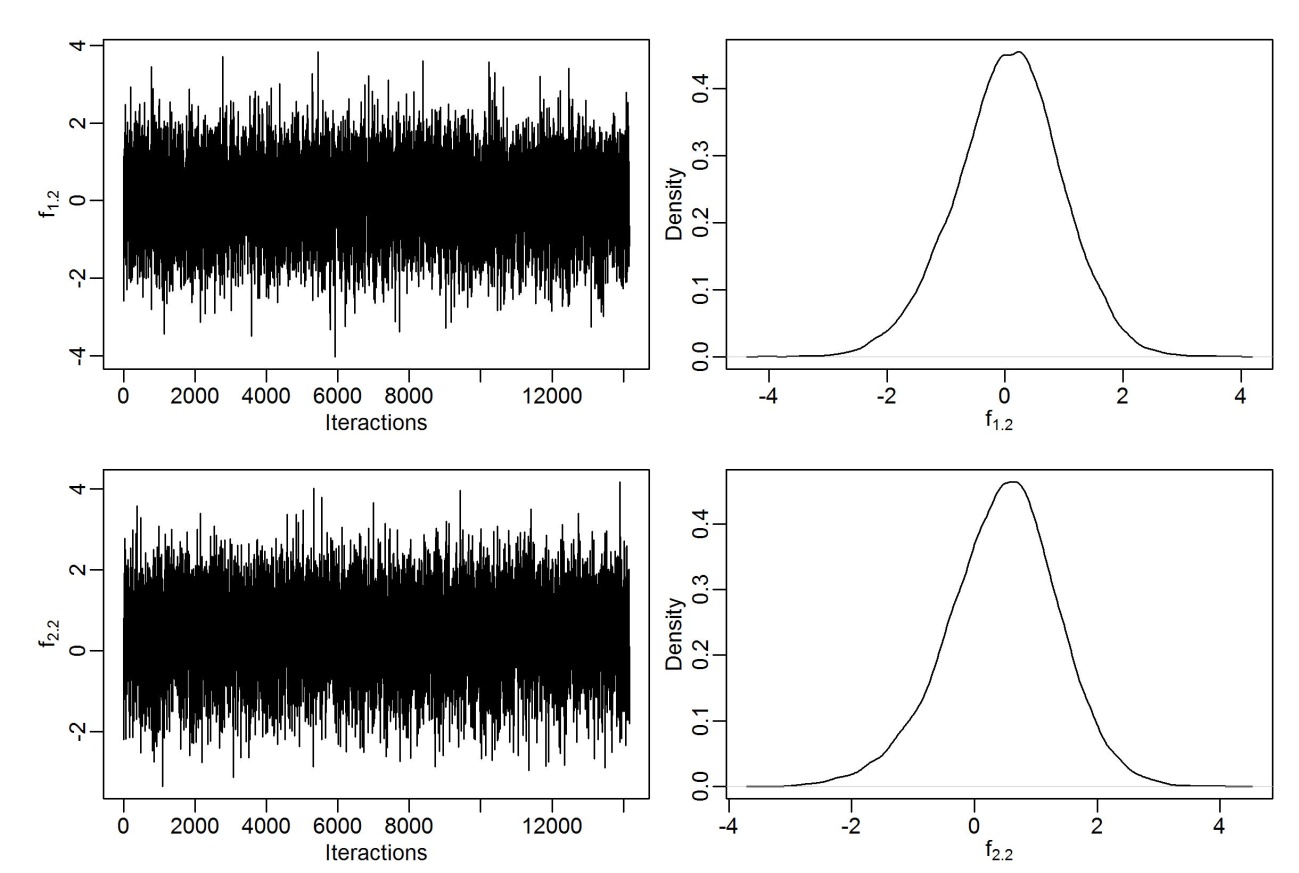
**Figure 1** - Traces and a posteriori densities for the first two coordinates relating to factorial scores $f_{1,2}$ and $f_{2,2}$ (simulated data).


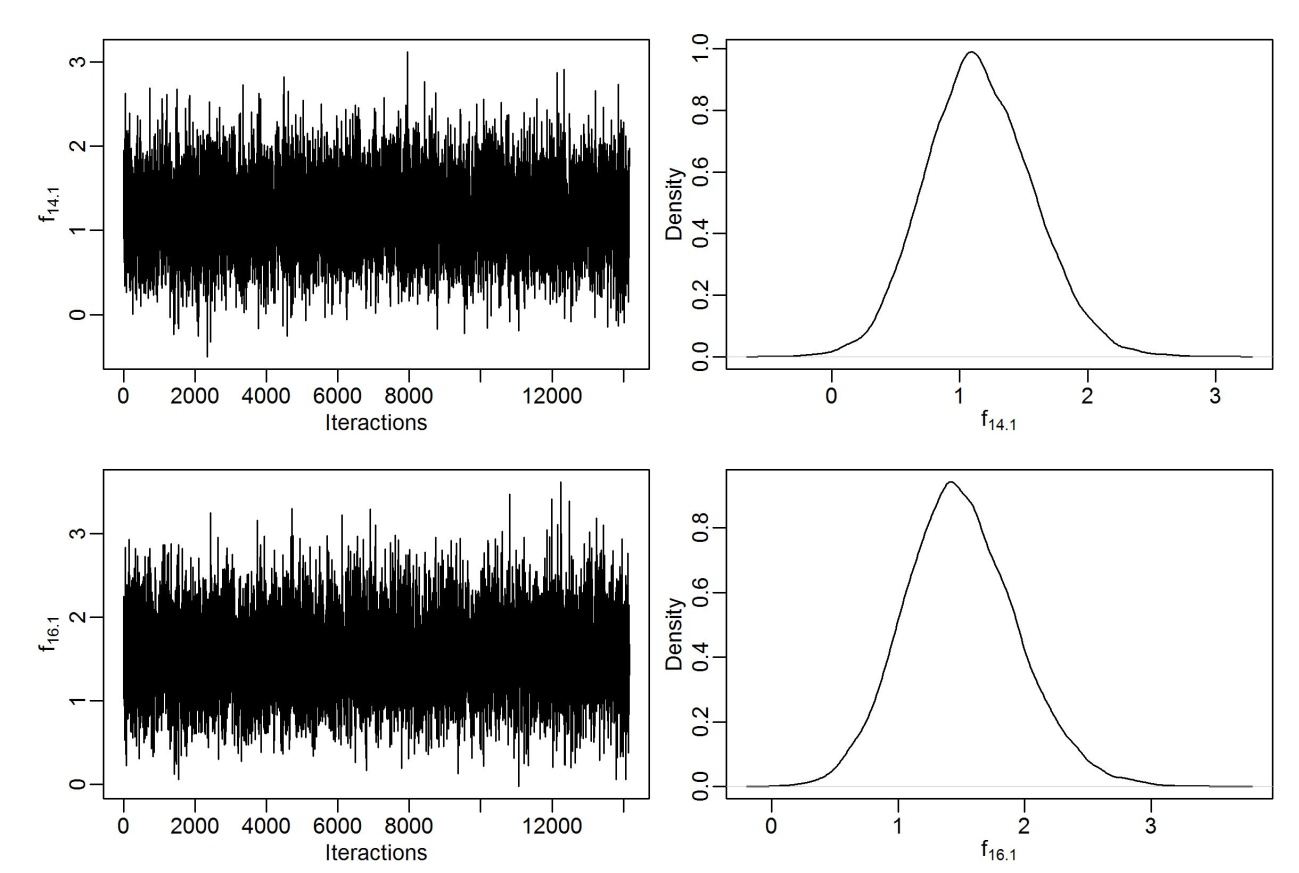


**Figure 2** - Traces and a posteriori densities for the first coordinates relating to factorial scores $f_{14,1}$ and $f_{16,1}$ (simulated data).


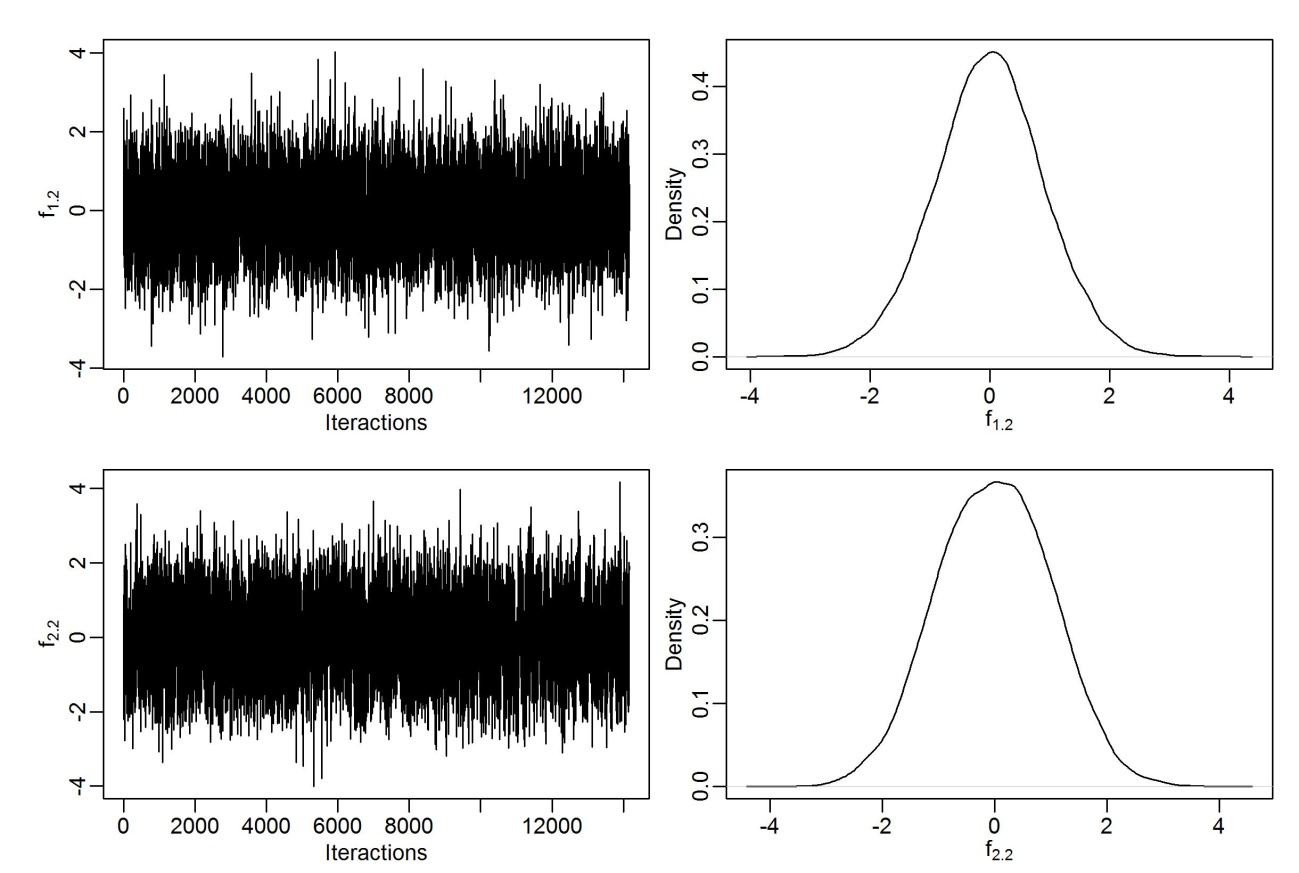


**Figure 3** - Traces and a posteriori densities for the second coordinates relating to factorial scores $f_{2,1}$ and $f_{2,2}$ (simulated data).


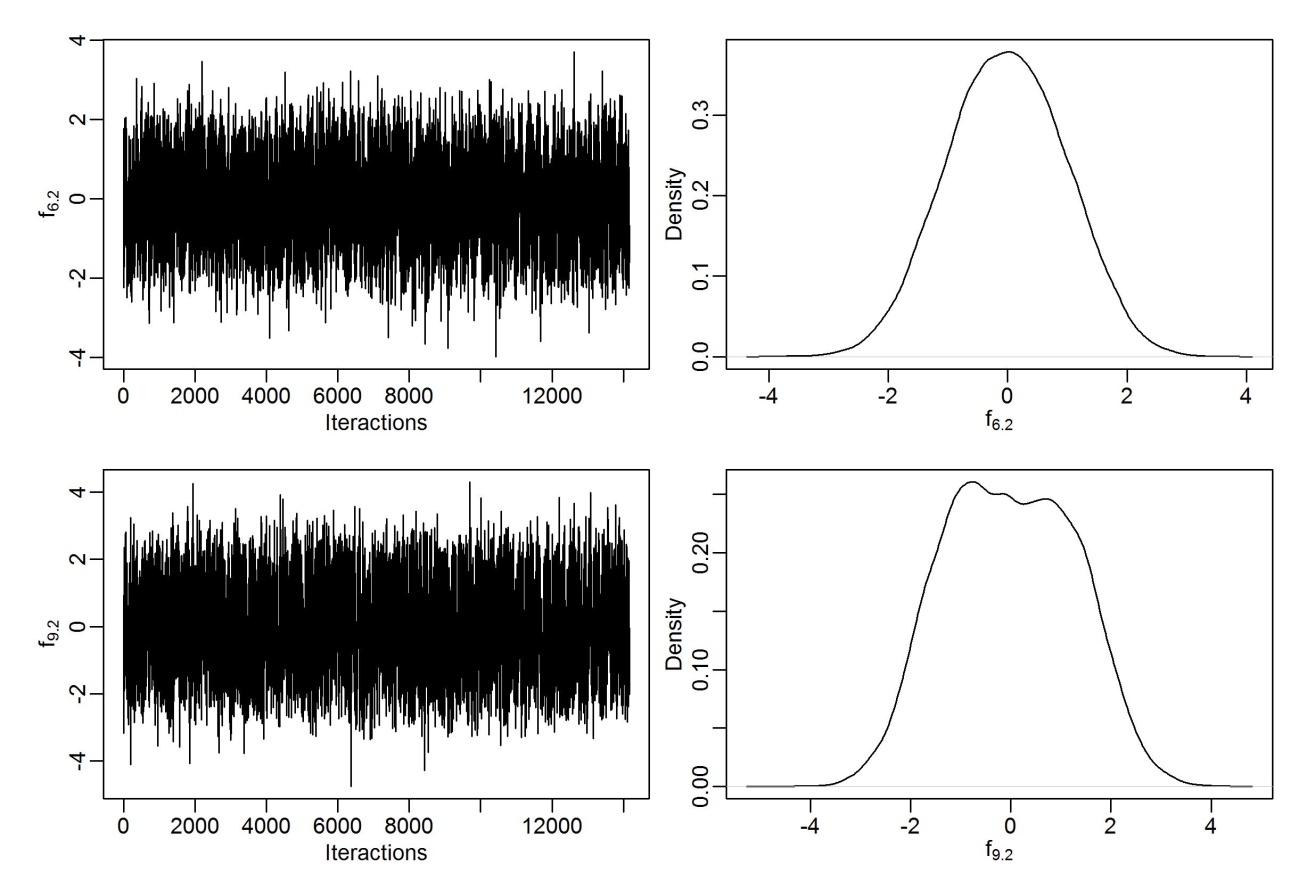


**Figure 4** - Traces and a posteriori densities for the second coordinates relating to factorial scores $f_{6,2}$ e $f_{9,2}$ (simulated data).


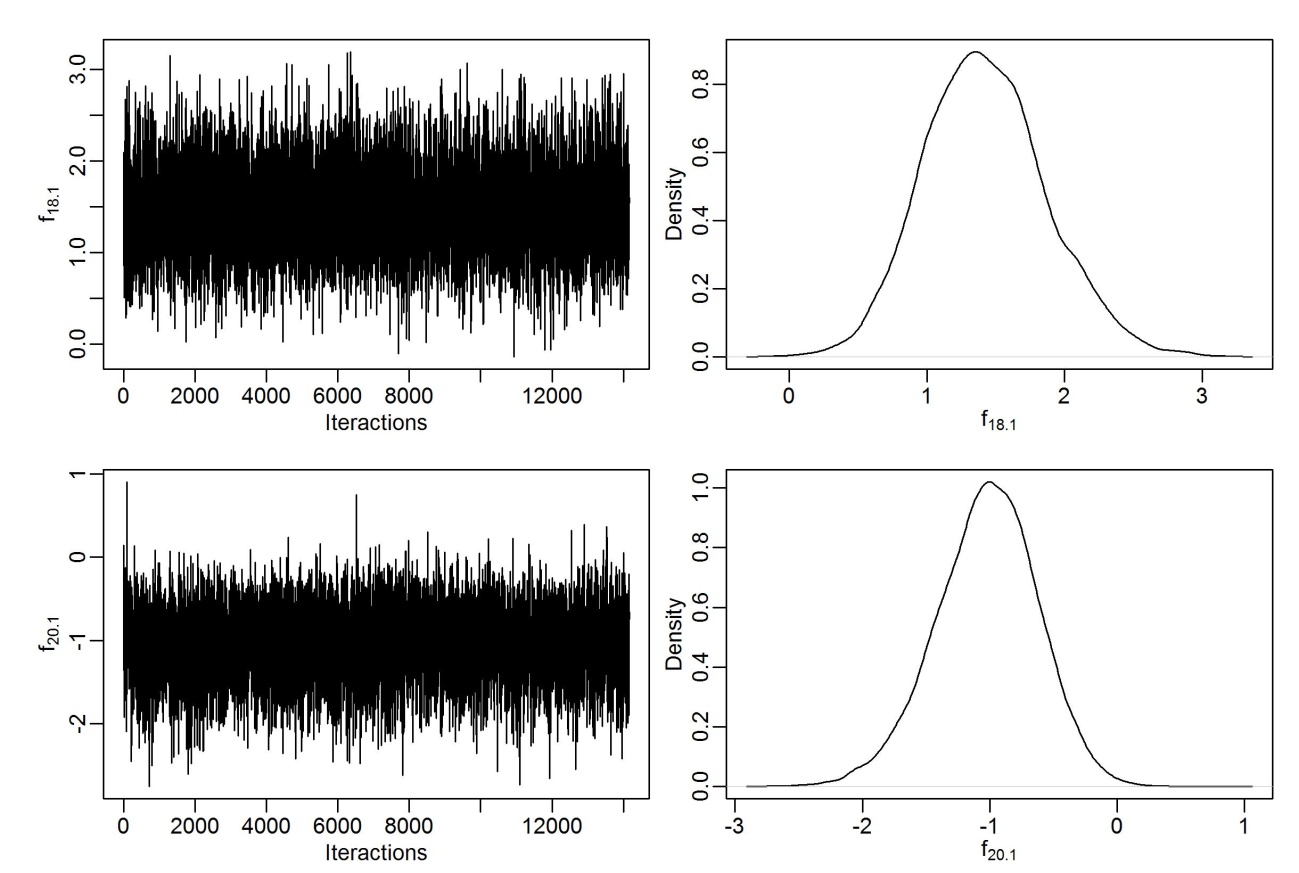


**Figure 5** - Traces and a posteriori densities for the first coordinates relating to factorial scores $f_{18,1}$ and $f_{20,1}$ (simulated data).


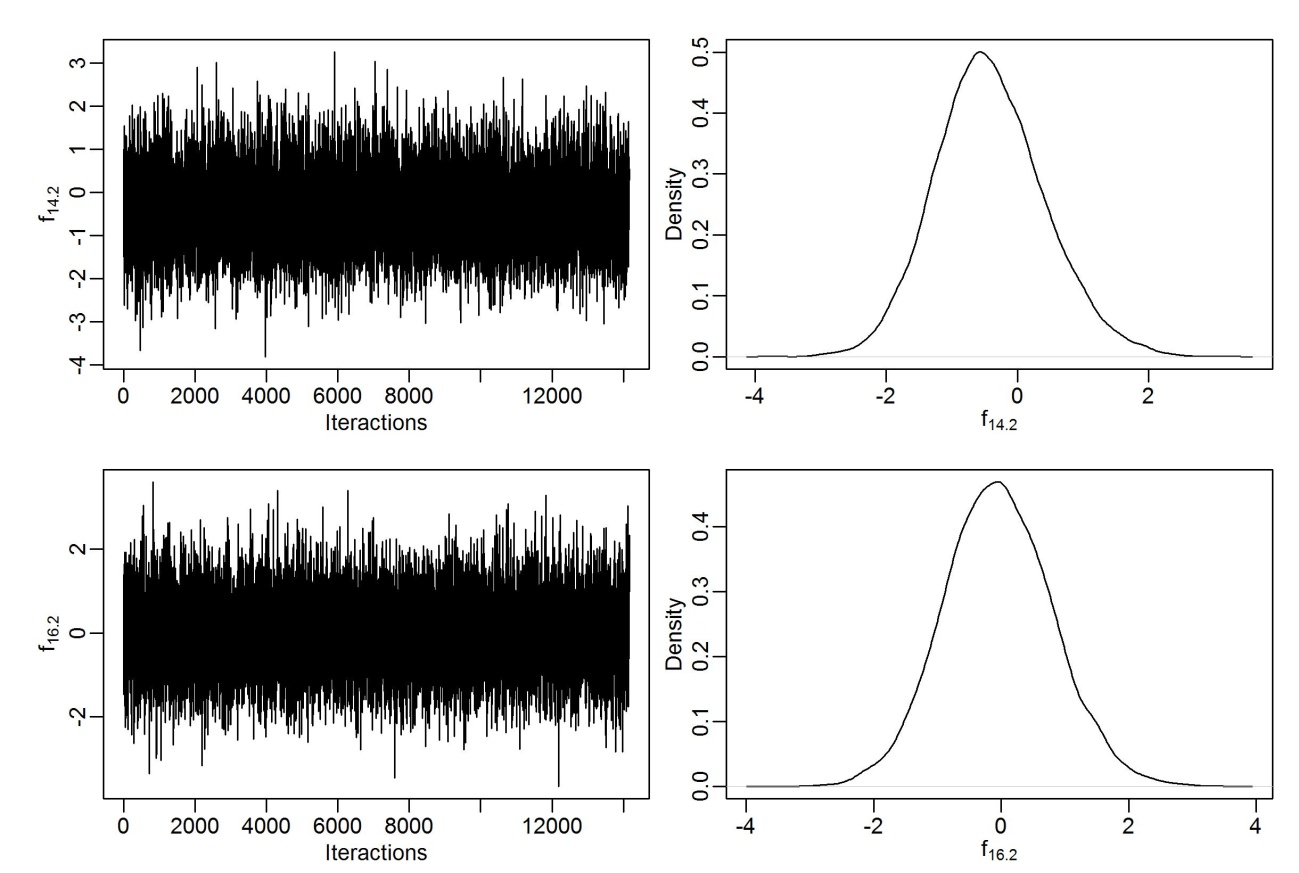


**Figure 6** - Traces and a posteriori densities for the second coordinates relating to factorial scores $f_{14,2}$ and $f_{16,2}$ (simulated data).


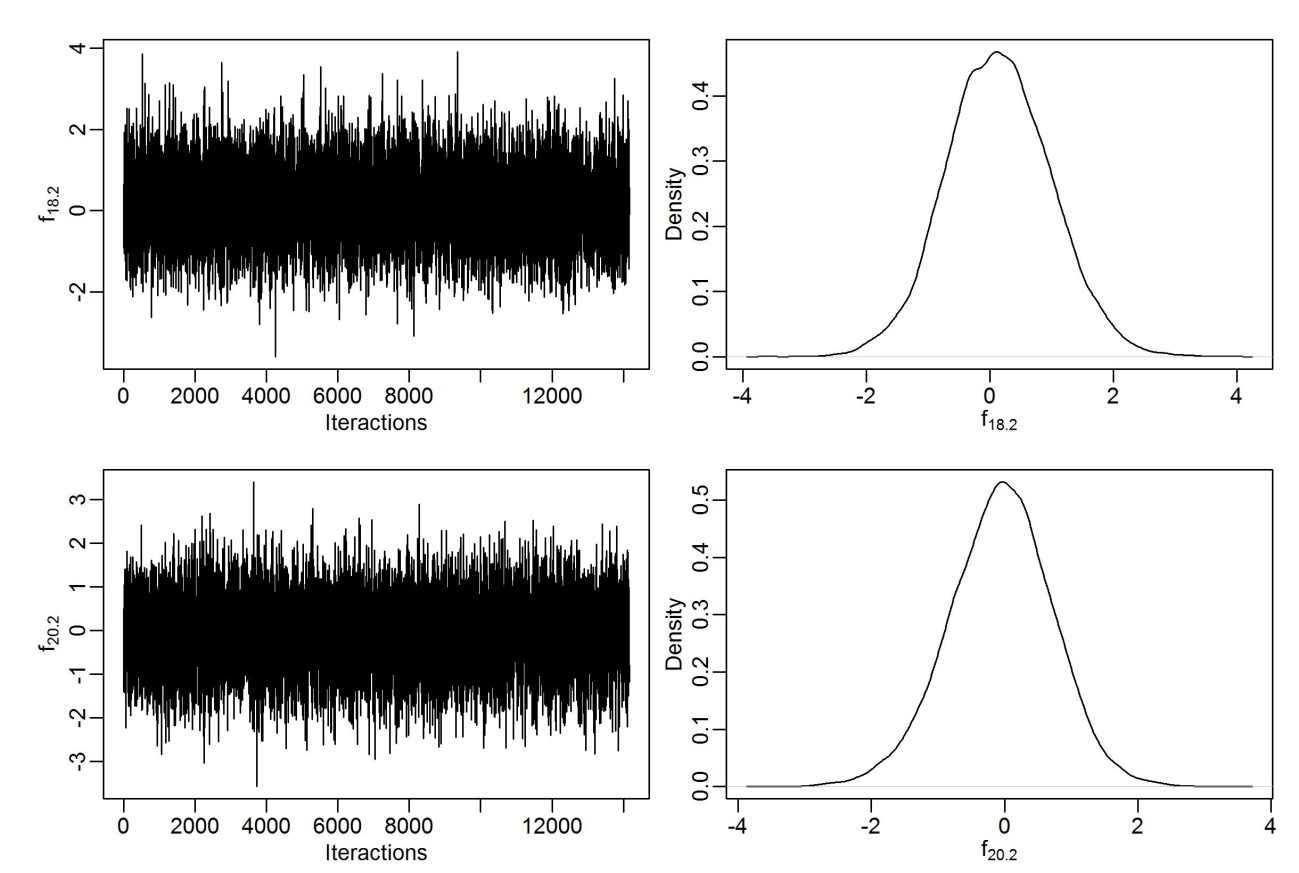
**Figure 7** - Traces and a posteriori densities for the second coordinates relating to factorial scores $f_{18,2}$ and $f_{20,2}$ (simulated data).


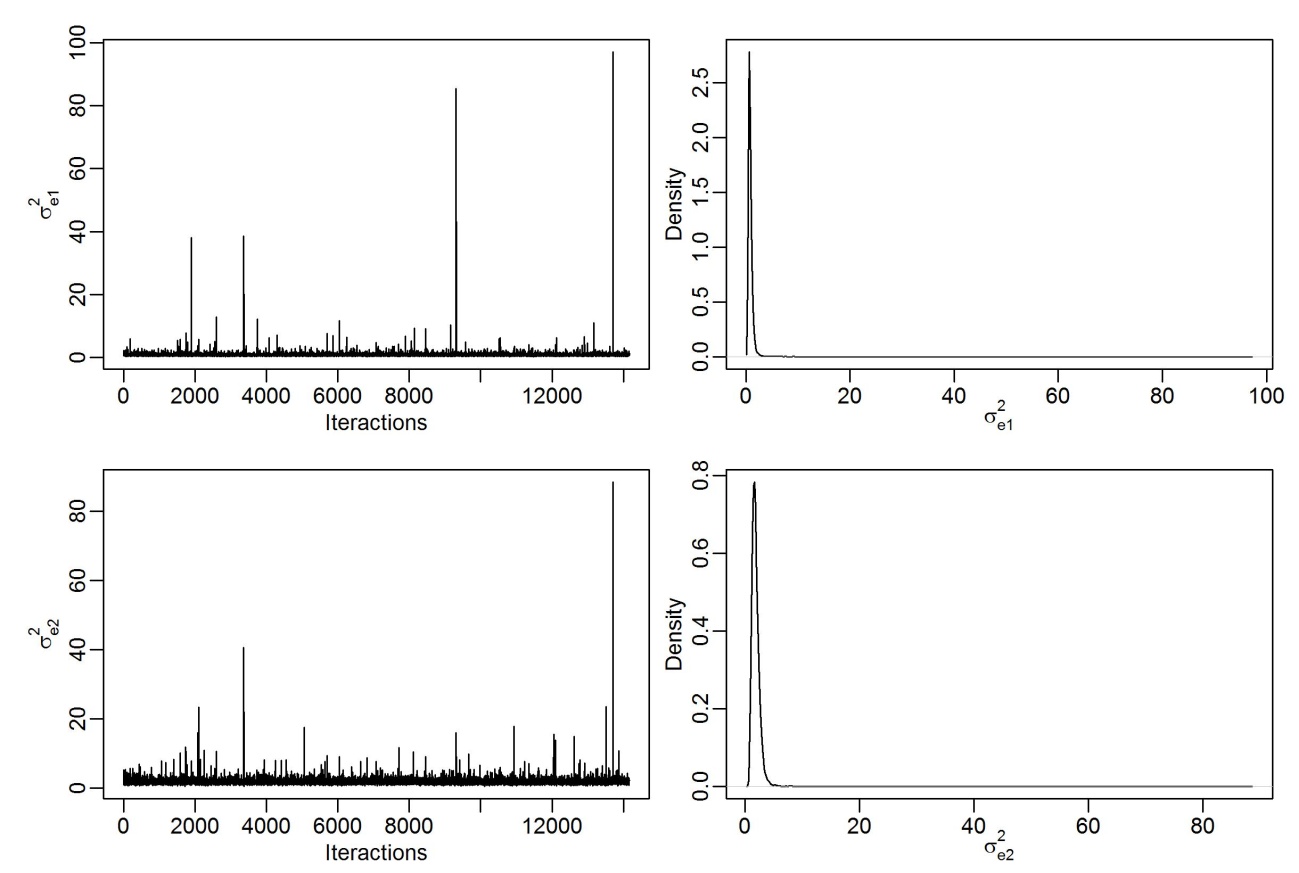
**Figure 8** - Traces and a posteriori densities for the components of variance $\sigma_{e1}^{2}$ and$\sigma_{e2}^{2}$ (simulated data).


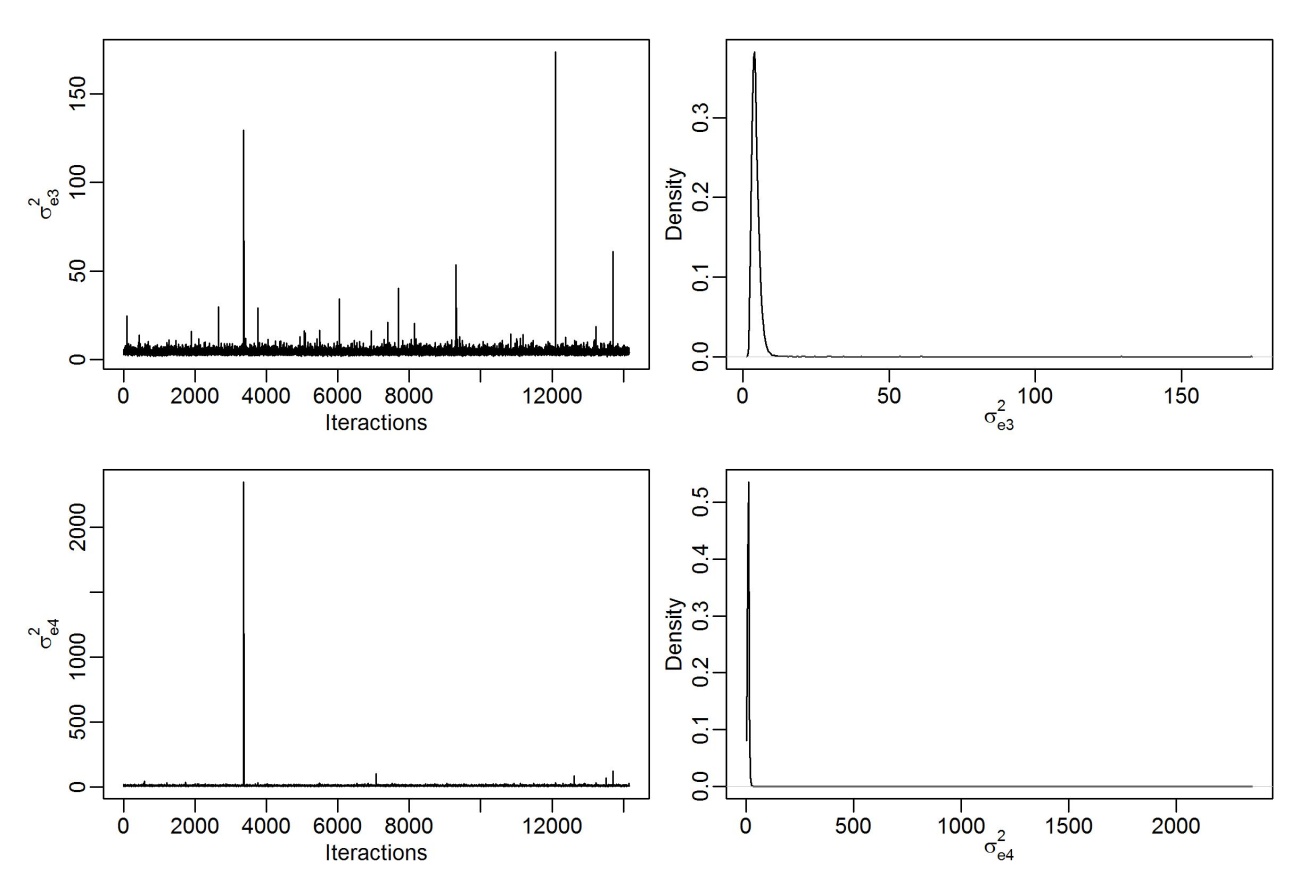
**Figure 9** - Traces and a posteriori densities for the components of variance $\sigma_{e3}^{2}$ and$\sigma_{e4}^{2}$ (simulated data).


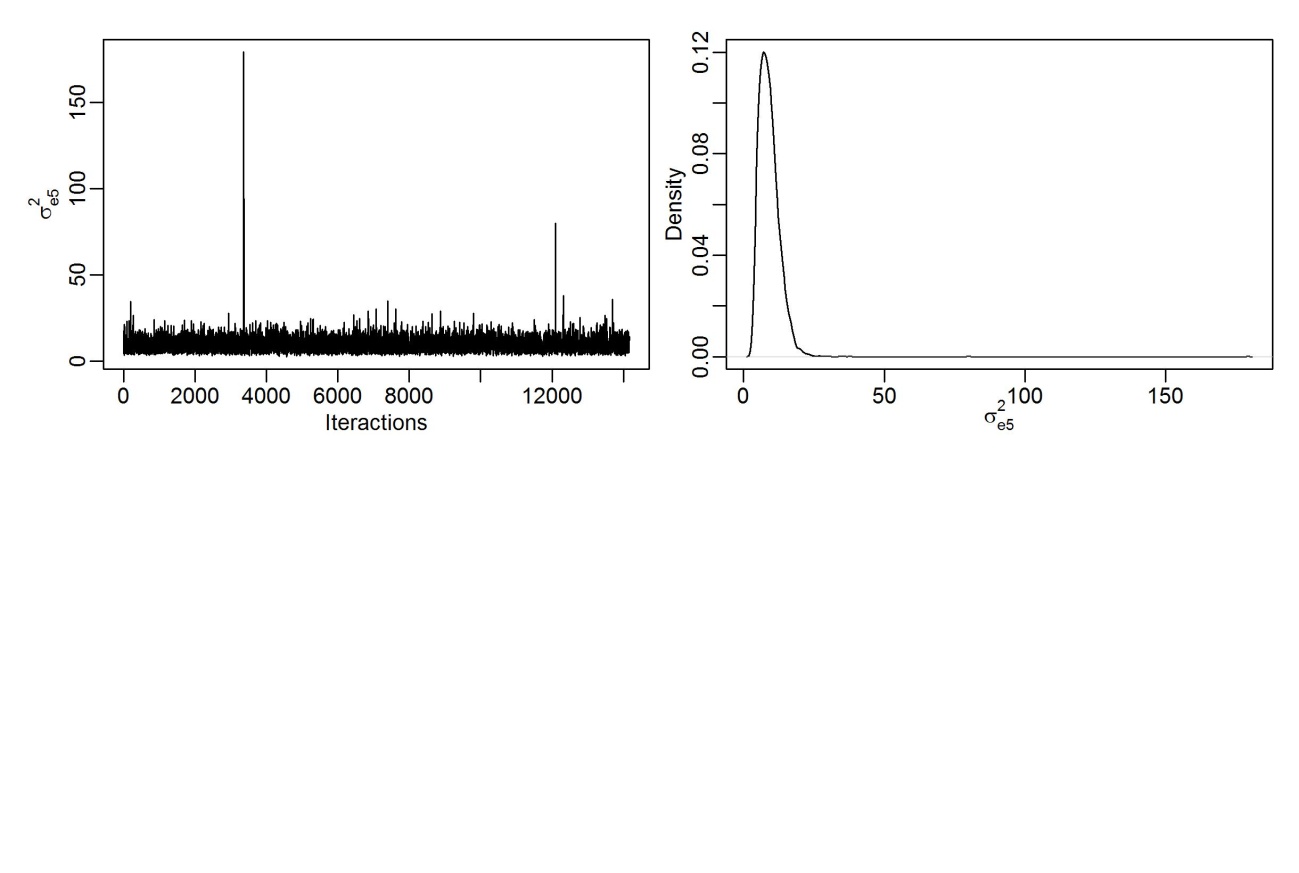
**Figure 10** - Traces and a posteriori density for the component of variance $\sigma_{e5}^{2}$ (simulated data).
