# Supplementary material for: Bayesian factor analytic model: An approach in multiple environment trials
Source: PLoS One. 2019 Aug 22;14(8):e0220290. doi: 10.1371/journal.pone.0220290 (PMC6705866; doi:10.1371/journal.pone.0220290)
Supplement: S1 Table — (DOCX) [file pone.0220290.s005.docx]

**S1 Table**

**Table S1** – Description of the environments where the experiments were conducted.

| Environment | City | Latitude | Longitude | Altitude (m) |
| --- | --- | --- | --- | --- |
| E1 | Guarapuava –PR | 25°23'S | 51°27'W | 1,120 |
| E2 | Vacaria –RS | 28°30'S | 50°56'O | 971 |
| E3 | Ipiranga –PR | 25°01'S | 50°35'O | 800 |
| E4 | Sananduva –RS | 27°57'S | 51°48'W | 636 |
| E5 | Faxinal –PR | 24°0'S | 51°19'W | 820 |
| E6 | Presidente Olegario -MG | 18°25'S | 46°25'W | 947 |
| E7 | Uberaba –MG | 19°44'S | 47°55'W | 823 |
| E8 | Capinópolis –MG | 18°40'S | 49°34'W | 530 |
| E9 | Araguari –MG | 18°38'S | 48°11'W | 1,013 |
