# Supplementary material for: Bayesian factor analytic model: An approach in multiple environment trials
Source: PLoS One. 2019 Aug 22;14(8):e0220290. doi: 10.1371/journal.pone.0220290 (PMC6705866; doi:10.1371/journal.pone.0220290)
Supplement: S2 Table — (DOCX) [file pone.0220290.s006.docx]

**S2 Table**

**Table S2** - Posterior means (PM), regions of credibility (95%. LL: lower limit. UL: upper limit) and estimates of restricted maximum likelihood (REML) of FA-AI and genotypic scores ($\mathbf{f}_{i1}-\mathbf{f}_{i2}$), simulated data.

| Par. | REML | PM | sd | LL | UL | Par. | REML | PM | sd | LL | UL |
| --- | --- | --- | --- | --- | --- | --- | --- | --- | --- | --- | --- |
| $f_{1,1}$ | -0.959 | -1.141 | 0.423 | -1.970 | -0.310 | $f_{1,2}$ | -0.529 | 0.062 | 0.904 | -1.722 | 1.837 |
| $f_{2,1}$ | -1.351 | -1.261 | 0.445 | -2.135 | -0.414 | $f_{2,2}$ | -0.024 | 0.454 | 0.908 | -1.447 | 2.173 |
| $f_{3,1}$ | 0.043 | -0.093 | 0.367 | -0.824 | 0.621 | $f_{3,2}$ | -0.463 | -0.247 | 0.733 | -1.699 | 1.238 |
| $f_{4,1}$ | 1.127 | 0.496 | 0.481 | -0.456 | 1.410 | $f_{4,2}$ | -1.538 | -1.092 | 0.966 | -2.778 | 1.009 |
| $f_{5,1}$ | -0.841 | -0.787 | 0.393 | -1.607 | -0.064 | $f_{5,2}$ | -0.134 | 0.203 | 0.811 | -1.357 | 1.846 |
| $f_{6,1}$ | 0.735 | 1.066 | 0.452 | 0.222 | 1.970 | $f_{6,2}$ | 1.438 | 0.581 | 0.809 | -0.992 | 2.200 |
| $f_{7,1}$ | -0.294 | -0.222 | 0.375 | -0.956 | 0.512 | $f_{7,2}$ | -0.206 | -0.115 | 0.819 | -1.778 | 1.479 |
| $f_{8,1}$ | -1.229 | -0.816 | 0.425 | -1.684 | -0.008 | $f_{8,2}$ | 0.692 | 0.659 | 0.871 | -1.183 | 2.266 |
| $f_{9,1}$ | -1.678 | -1.123 | 0.474 | -2.056 | -0.211 | $f_{9,2}$ | 0.762 | 0.712 | 1.081 | -1.495 | 2.741 |
| $f_{10,1}$ | -0.526 | -0.601 | 0.386 | -1.397 | 0.104 | $f_{10,2}$ | -0.627 | -0.247 | 0.794 | -1.844 | 1.345 |
| $f_{11,1}$ | -0.149 | -0.471 | 0.397 | -1.281 | 0.277 | $f_{11,2}$ | -0.965 | -0.433 | 0.788 | -1.975 | 1.163 |
| $f_{12,1}$ | -0.172 | -0.146 | 0.367 | -0.871 | 0.577 | $f_{12,2}$ | -0.046 | -0.038 | 0.757 | -1.565 | 1.475 |
| $f_{13,1}$ | 0.509 | 0.900 | 0.454 | 0.046 | 1.796 | $f_{13,2}$ | 1.566 | 0.717 | 0.880 | -1.087 | 2.413 |
| $f_{14,1}$ | 1.270 | 1.156 | 0.419 | 0.371 | 2.002 | $f_{14,2}$ | -0.085 | -0.421 | 0.834 | -2.029 | 1.249 |
| $f_{15,1}$ | 0.078 | 0.239 | 0.378 | -0.492 | 0.984 | $f_{15,2}$ | 0.617 | 0.312 | 0.768 | -1.233 | 1.845 |
| $f_{16,1}$ | 1.439 | 1.498 | 0.444 | 0.642 | 2.387 | $f_{16,2}$ | 0.655 | -0.044 | 0.856 | -1.678 | 1.669 |
| $f_{17,1}$ | 0.745 | 0.565 | 0.399 | -0.2192 | 1.356 | $f_{17,2}$ | -0.538 | -0.519 | 0.804 | -2.006 | 1.186 |
| $f_{18,1}$ | 1.369 | 1.424 | 0.446 | 0.555 | 2.291 | $f_{18,2}$ | 0.938 | 0.150 | 0.847 | -1.528 | 1.802 |
| $f_{19,1}$ | 0.825 | 0.359 | 0.413 | -0.484 | 1.144 | $f_{19,2}$ | -0.934 | -0.653 | 0.831 | -2.259 | 1.041 |
| $f_{20,1}$ | -0.936 | -1.024 | 0.401 | -1.848 | -0.275 | $f_{20,2}$ | -0.589 | -0.058 | 0.780 | -1.625 | 1.446 |

Par.= Parameter
