# Supplementary material for: Bayesian factor analytic model: An approach in multiple environment trials
Source: PLoS One. 2019 Aug 22;14(8):e0220290. doi: 10.1371/journal.pone.0220290 (PMC6705866; doi:10.1371/journal.pone.0220290)
Supplement: S3 Table — (DOCX) [file pone.0220290.s007.docx]

**S3 Table**

**Table S3** - Posterior means (PM), regions of credibility (95%. LL: lower limit. UL: upper limit) for the first two factor loadings ($\lambda_{1}\mathbf{\alpha}_{1}-\lambda_{2}\mathbf{\alpha}_{2}$), real data.

|  |  |  | HPD 95% | |  |  |  | HPD 95% | |
| --- | --- | --- | --- | --- | --- | --- | --- | --- | --- |
| Par. | PM | sd | LL | UL | Par. | PM | sd | LL | UL |
| $\lambda_{1}\alpha_{1,1}$ | 0.190 | 0.178 | -0.179 | 0.516 | $\lambda_{2}\alpha_{1,2}$ | 0.070 | 0.231 | -0.432 | 0.477 |
| $\lambda_{1}\alpha_{2,1}$ | 0.625 | 0.179 | 0.283 | 0.980 | $\lambda_{2}\alpha_{2,2}$ | 0.533 | 0.188 | 0.106 | 0.887 |
| $\lambda_{1}\alpha_{3,1}$ | 0.291 | 0.151 | 0.002 | 0.591 | $\lambda_{2}\alpha_{3,2}$ | 0.197 | 0.207 | -0.242 | 0.568 |
| $\lambda_{1}\alpha_{4,1}$ | 0.091 | 0.140 | -0.195 | 0.359 | $\lambda_{2}\alpha_{4,2}$ | 0.340 | 0.169 | -0.015 | 0.664 |
| $\lambda_{1}\alpha_{5,1}$ | 0.600 | 0.176 | 0.262 | 0.953 | $\lambda_{2}\alpha_{5,2}$ | 0.253 | 0.182 | -0.118 | 0.606 |
| $\lambda_{1}\alpha_{6,1}$ | 0.794 | 0.199 | 0.379 | 1.163 | $\lambda_{2}\alpha_{6,2}$ | -0.216 | 0.310 | -0.761 | 0.454 |
| $\lambda_{1}\alpha_{7,1}$ | 0.944 | 0.200 | 0.547 | 1.327 | $\lambda_{2}\alpha_{7,2}$ | -0.386 | 0.216 | -0.810 | 0.066 |
| $\lambda_{1}\alpha_{8,1}$ | 1.223 | 0.234 | 0.760 | 1.665 | $\lambda_{2}\alpha_{8,2}$ | 0.136 | 0.277 | -0.426 | 0.657 |
| $\lambda_{1}\alpha_{9,1}$ | 0.440 | 0.150 | 0.134 | 0.720 | $\lambda_{2}\alpha_{9,2}$ | -0.184 | 0.179 | -0.552 | 0.165 |
| $\lambda_{1}\alpha_{10,1}$ | 0.693 | 0.195 | 0.311 | 1.074 | $\lambda_{2}\alpha_{10,2}$ | -0.227 | 0.261 | -0.707 | 0.328 |
