# Supplementary material for: Bayesian factor analytic model: An approach in multiple environment trials
Source: PLoS One. 2019 Aug 22;14(8):e0220290. doi: 10.1371/journal.pone.0220290 (PMC6705866; doi:10.1371/journal.pone.0220290)
Supplement: S4 Table — (DOCX) [file pone.0220290.s008.docx]

**S4 Table**

**Table S4** - Posterior means (PM), regions of credibility (95%. LL: lower limit. UL: upper limit) for the first two factor scores ($\mathbf{f}_{i1}-\mathbf{f}_{i2}$), real data.

|  |  |  | HPD 95% | |  |  |  | HPD 95% | |
| --- | --- | --- | --- | --- | --- | --- | --- | --- | --- |
| Par. | PM | sd | LL | UL | Par. | PM | sd | LL | UL |
| $f_{1.1}$ | 1.884 | 0.310 | 1.273 | 2.477 | $f_{1.2}$ | 0.098 | 1.068 | -1.830 | 2.237 |
| $f_{2.1}$ | 1.366 | 0.354 | 0.730 | 2.107 | $f_{2.2}$ | 0.101 | 1.030 | -1.884 | 2.046 |
| $f_{3.1}$ | 1.398 | 0.261 | 0.896 | 1.911 | $f_{3.2}$ | -0.051 | 0.784 | -1.521 | 1.438 |
| $f_{4.1}$ | 0.540 | 0.314 | -0.040 | 1.181 | $f_{4.2}$ | -0.061 | 0.818 | -1.579 | 1.439 |
| $f_{5.1}$ | 1.659 | 0.312 | 1.069 | 2.289 | $f_{5.2}$ | 0.060 | 0.781 | -1.475 | 1.572 |
| $f_{6.1}$ | 1.251 | 0.255 | 0.752 | 1.754 | $f_{6.2}$ | -0.114 | 1.111 | -1.851 | 1.729 |
| $f_{7.1}$ | 2.585 | 0.366 | 1.875 | 3.304 | $f_{7.2}$ | 0.067 | 0.945 | -1.735 | 1.893 |
| $f_{8.1}$ | 0.747 | 0.269 | 0.213 | 1.273 | $f_{8.2}$ | -0.236 | 1.993 | -2.851 | 2.850 |
| $f_{9.1}$ | 0.631 | 0.252 | 0.147 | 1.140 | $f_{9.2}$ | 0.069 | 0.743 | -1.291 | 1.407 |
| $f_{10.1}$ | 1.210 | 0.265 | 0.685 | 1.719 | $f_{10.2}$ | 0.139 | 1.101 | -1.901 | 1.957 |
| $f_{11.1}$ | -0.114 | 0.245 | -0.569 | 0.385 | $f_{11.2}$ | -0.058 | 0.983 | -1.955 | 1.719 |
| $f_{12.1}$ | 0.190 | 0.408 | -0.597 | 0.994 | $f_{12.2}$ | 0.046 | 0.748 | -1.406 | 1.465 |
| $f_{13.1}$ | 0.124 | 0.435 | -0.730 | 0.992 | $f_{13.2}$ | -0.035 | 0.866 | -1.703 | 1.658 |
| $f_{14.1}$ | -0.312 | 0.433 | -1.163 | 0.533 | $f_{14.2}$ | 0.061 | 0.843 | -1.571 | 1.654 |
| $f_{15.1}$ | 0.292 | 0.425 | -0.504 | 1.160 | $f_{15.2}$ | 0.036 | 0.789 | -1.557 | 1.520 |
| $f_{16.1}$ | 0.175 | 0.454 | -0.666 | 1.105 | $f_{16.2}$ | -0.019 | 0.853 | -1.641 | 1.709 |
| $f_{17.1}$ | 0.197 | 0.442 | -0.672 | 1.064 | $f_{17.2}$ | -0.007 | 0.689 | -1.315 | 1.350 |
| $f_{18.1}$ | 0.104 | 0.435 | -0.720 | 0.964 | $f_{18.2}$ | -0.072 | 0.942 | -1.782 | 1.747 |
| $f_{19.1}$ | -0.622 | 0.442 | -1.493 | 0.243 | $f_{19.2}$ | -0.058 | 0.790 | -1.635 | 1.456 |
| $f_{20.1}$ | 0.122 | 0.451 | -0.766 | 1.011 | $f_{20.2}$ | 0.070 | 1.078 | -1.906 | 2.106 |
| $f_{21.1}$ | 0.115 | 0.454 | -0.838 | 0.944 | $f_{21.2}$ | -0.132 | 1.188 | -2.205 | 2.044 |
| $f_{22.1}$ | -0.536 | 0.453 | -1.410 | 0.360 | $f_{22.2}$ | -0.042 | 0.859 | -1.679 | 1.665 |
| $f_{23.1}$ | -0.508 | 0.438 | -1.401 | 0.323 | $f_{23.2}$ | -0.003 | 0.653 | -1.317 | 1.248 |
| $f_{24.1}$ | -0.561 | 0.437 | -1.399 | 0.337 | $f_{24.2}$ | -0.063 | 0.946 | -1.767 | 1.789 |
| $f_{25.1}$ | -0.690 | 0.460 | -1.632 | 0.187 | $f_{25.2}$ | 0.106 | 1.320 | -2.285 | 2.411 |
| $f_{26.1}$ | -0.024 | 0.437 | -0.894 | 0.812 | $f_{26.2}$ | 0.037 | 0.741 | -1.494 | 1.436 |
| $f_{27.1}$ | 0.187 | 0.462 | -0.748 | 1.078 | $f_{27.2}$ | -0.057 | 0.830 | -1.625 | 1.563 |
| $f_{28.1}$ | -0.062 | 0.433 | -0.899 | 0.786 | $f_{28.2}$ | -0.021 | 0.734 | -1.474 | 1.418 |
| $f_{29.1}$ | -0.497 | 0.430 | -1.340 | 0.347 | $f_{29.2}$ | -0.058 | 0.732 | -1.457 | 1.407 |
| $f_{30.1}$ | -0.481 | 0.468 | -1.414 | 0.422 | $f_{30.2}$ | 0.123 | 1.165 | -2.079 | 2.154 |
| $f_{31.1}$ | -0.833 | 0.489 | -1.768 | 0.150 | $f_{31.2}$ | -0.048 | 0.779 | -1.556 | 1.445 |
| $f_{32.1}$ | -1.460 | 0.509 | -2.428 | -0.429 | $f_{32.2}$ | -0.085 | 1.079 | -2.203 | 1.924 |
| $f_{33.1}$ | -0.721 | 0.507 | -1.770 | 0.237 | $f_{33.2}$ | 0.098 | 1.087 | -1.901 | 2.097 |
| $f_{34.1}$ | 0.453 | 0.479 | -0.485 | 1.395 | $f_{34.2}$ | -0.012 | 0.708 | -1.348 | 1.443 |
| $f_{35.1}$ | -2.285 | 0.517 | -3.289 | -1.291 | $f_{35.2}$ | -0.098 | 1.125 | -2.167 | 2.026 |
| $f_{36.1}$ | -1.349 | 0.494 | -2.289 | -0.359 | $f_{36.2}$ | -0.032 | 0.758 | -1.525 | 1.422 |
| $f_{37.1}$ | -1.417 | 0.475 | -2.396 | -0.541 | $f_{37.2}$ | -0.042 | 0.751 | -1.480 | 1.476 |
| $f_{38.1}$ | 0.812 | 0.237 | 0.372 | 1.284 | $f_{38.2}$ | -0.095 | 0.922 | -1.474 | 1.485 |
| $f_{39.1}$ | -0.033 | 0.232 | -0.484 | 0.424 | $f_{39.2}$ | -0.103 | 0.929 | -1.635 | 1.493 |
| $f_{40.1}$ | 0.453 | 0.504 | -0.547 | 1.421 | $f_{40.2}$ | 0.009 | 0.758 | -1.499 | 1.484 |
| $f_{41.1}$ | -0.132 | 0.476 | -1.046 | 0.800 | $f_{41.2}$ | -0.009 | 0.768 | -1.530 | 1.502 |
| $f_{42.1}$ | -0.527 | 0.486 | -1.495 | 0.395 | $f_{42.2}$ | 0.146 | 1.456 | -2.500 | 2.444 |
| $f_{43.1}$ | -0.795 | 0.489 | -1.760 | 0.184 | $f_{43.2}$ | 0.022 | 0.724 | -1.425 | 1.407 |
| $f_{44.1}$ | -0.357 | 0.514 | -1.354 | 0.676 | $f_{44.2}$ | -0.075 | 1.132 | -2.162 | 2.028 |
| $f_{45.1}$ | -0.550 | 0.486 | -1.527 | 0.395 | $f_{45.2}$ | 0.176 | 1.628 | -2.656 | 2.694 |
| $f_{46.1}$ | -0.871 | 0.449 | -1.787 | -0.016 | $f_{46.2}$ | 0.032 | 0.749 | -1.372 | 1.582 |
| $f_{47.1}$ | -0.074 | 0.481 | -0.976 | 0.910 | $f_{47.2}$ | 0.103 | 1.199 | -2.132 | 2.192 |
| $f_{48.1}$ | -0.188 | 0.500 | -1.157 | 0.789 | $f_{48.2}$ | 0.071 | 0.845 | -1.606 | 1.691 |
| $f_{49.1}$ | -0.705 | 0.502 | -1.671 | 0.293 | $f_{49.2}$ | 0.152 | 1.390 | -2.311 | 2.362 |
| $f_{50.1}$ | -0.046 | 0.474 | -0.993 | 0.861 | $f_{50.2}$ | 0.004 | 0.685 | -1.315 | 1.390 |
