# Supplementary figures and images for: Bayesian factor analytic model: An approach in multiple environment trials
Source: PLoS One. 2019 Aug 22;14(8):e0220290. doi: 10.1371/journal.pone.0220290 (PMC6705866; doi:10.1371/journal.pone.0220290)

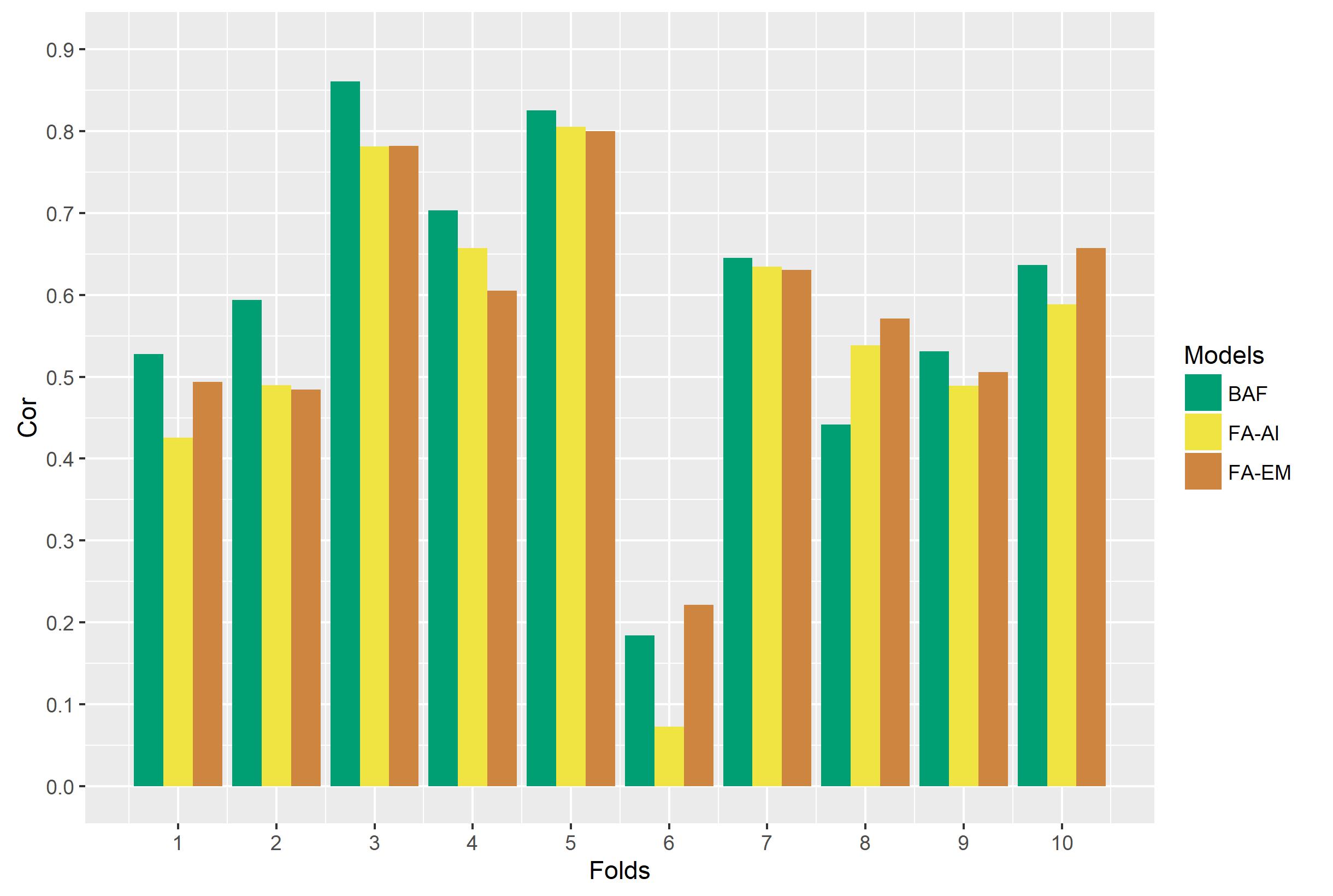

Supplement: S1 Fig — (JPEG) [file pone.0220290.s009.jpeg]

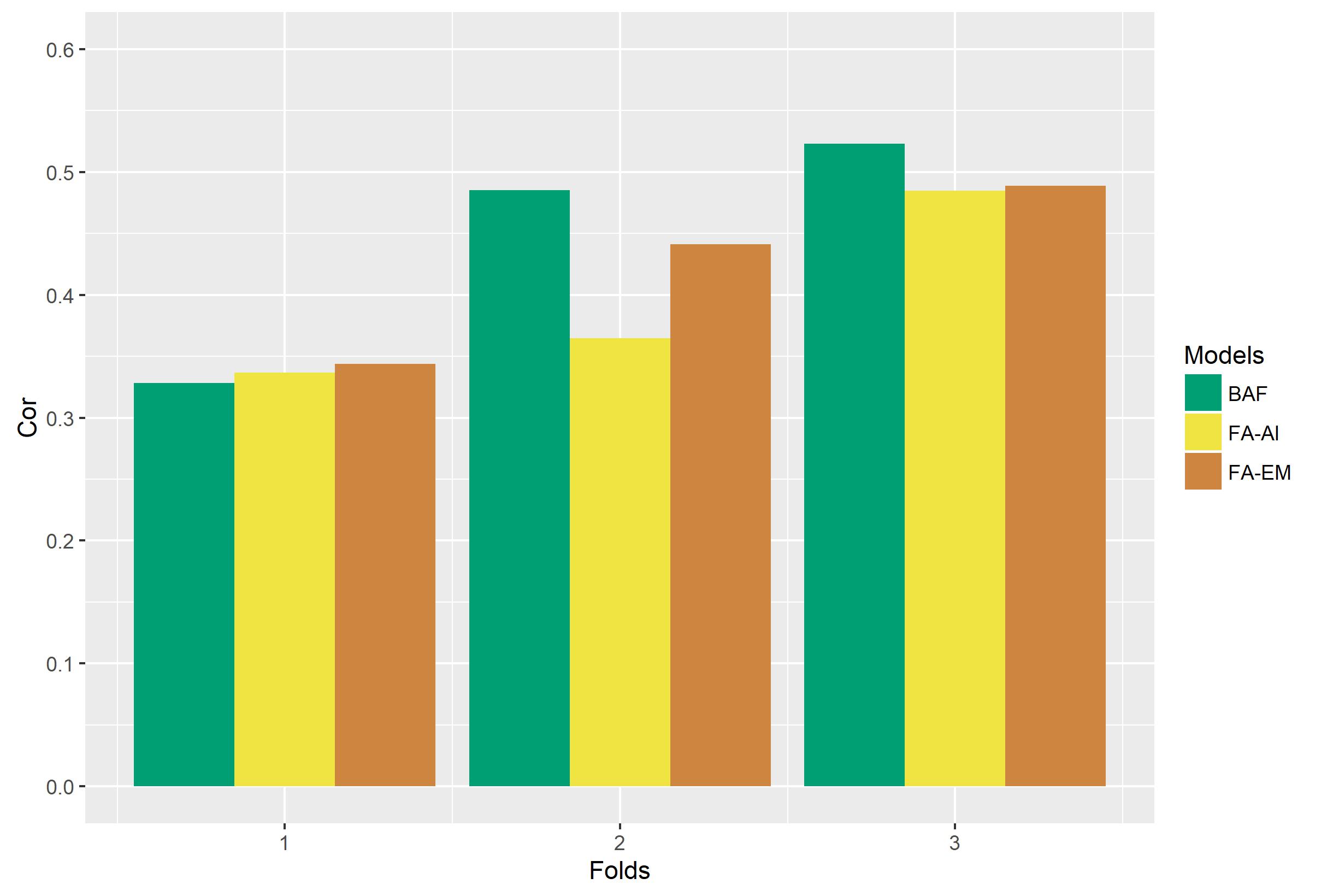

Supplement: S2 Fig — (JPEG) [file pone.0220290.s010.jpeg]

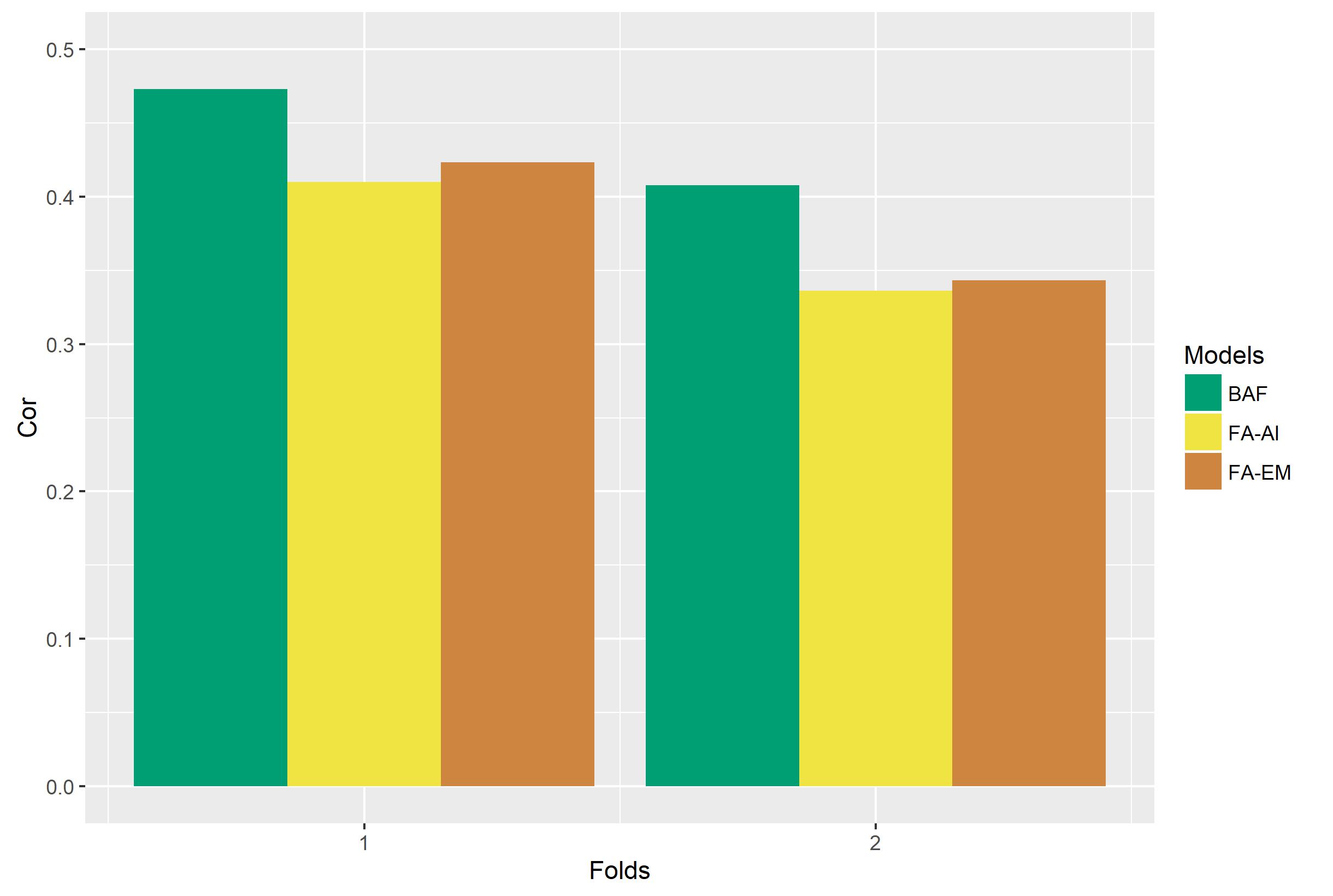

Supplement: S3 Fig — (JPEG) [file pone.0220290.s011.jpeg]

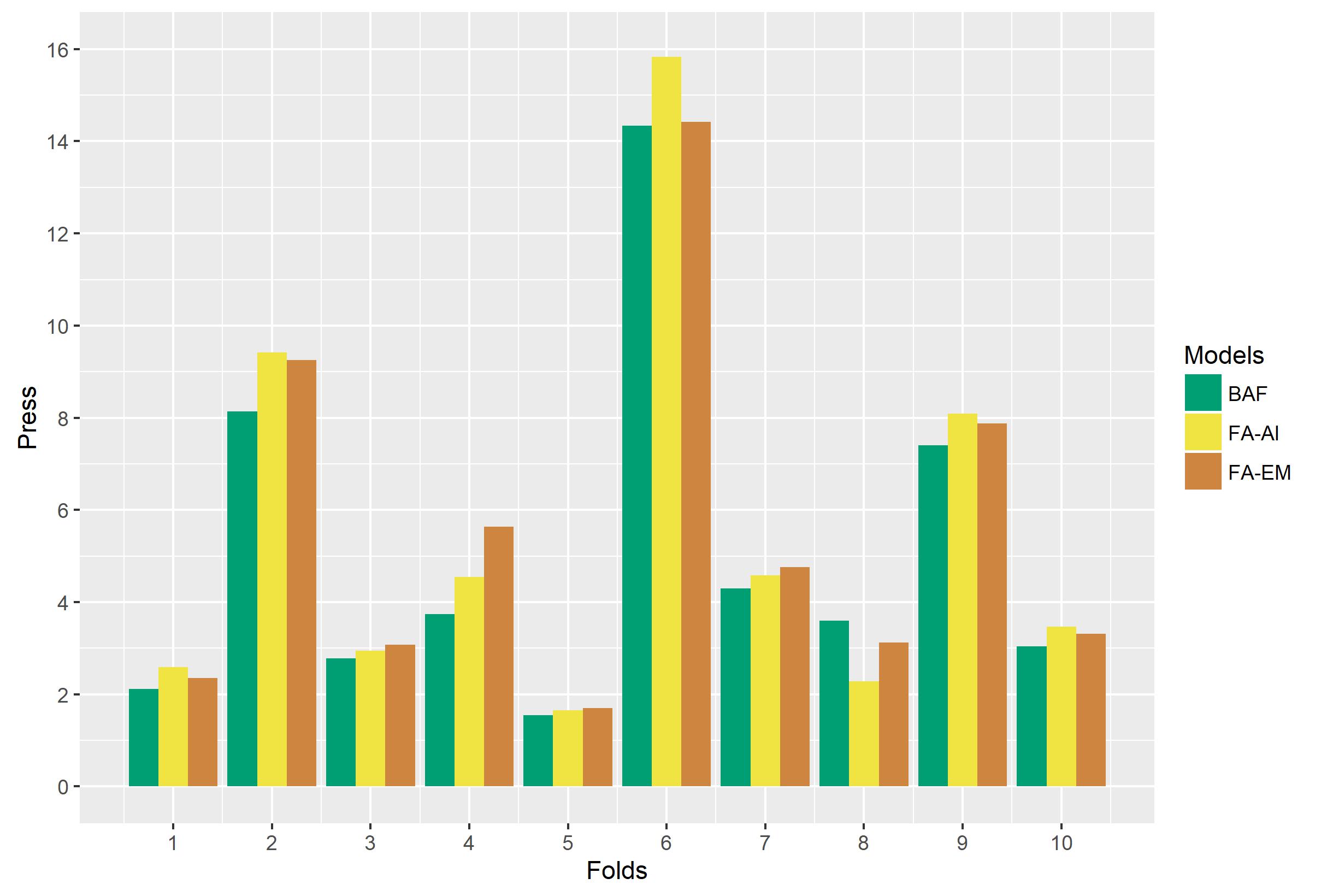

Supplement: S4 Fig — (JPEG) [file pone.0220290.s012.jpeg]

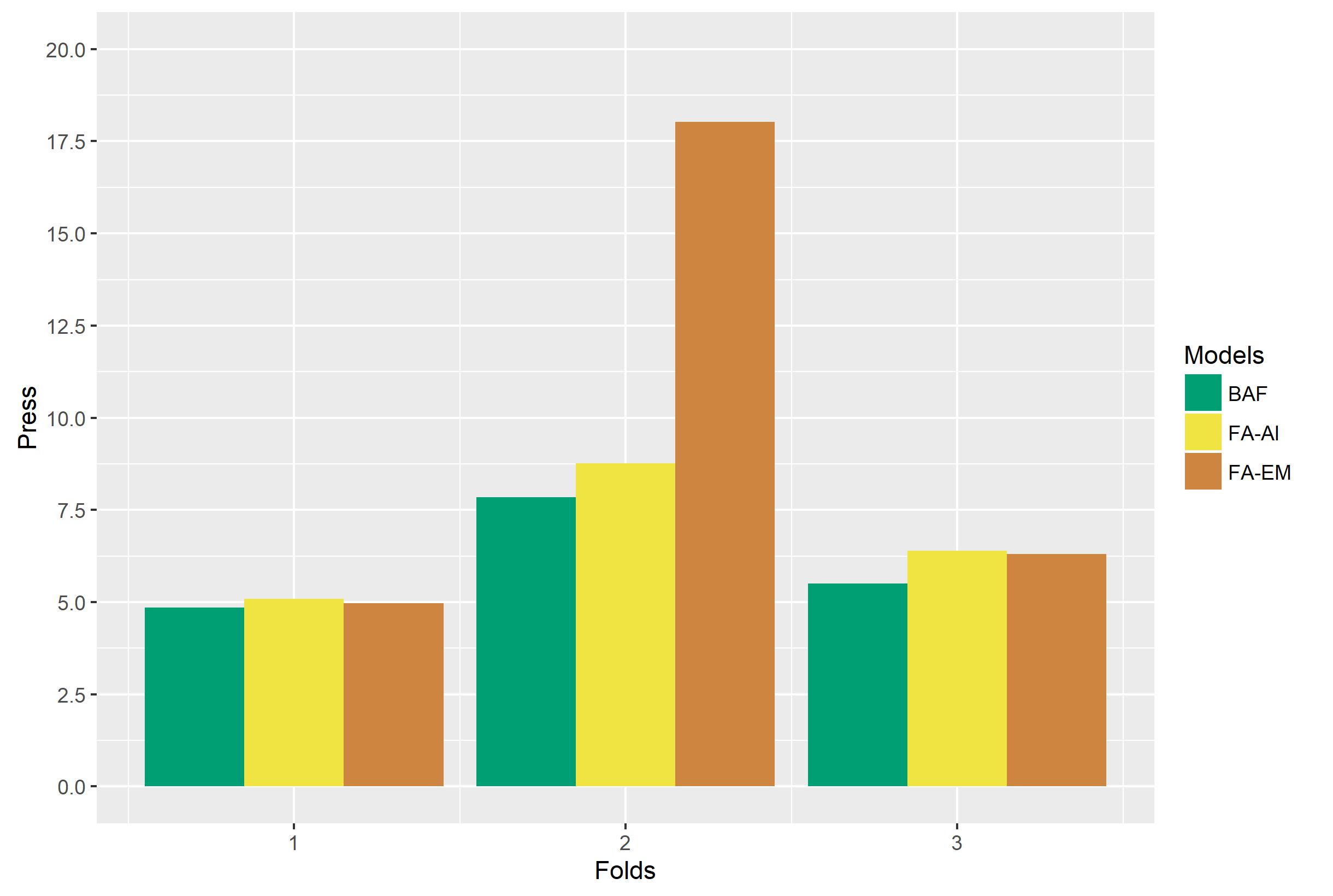

Supplement: S5 Fig — (JPEG) [file pone.0220290.s013.jpeg]

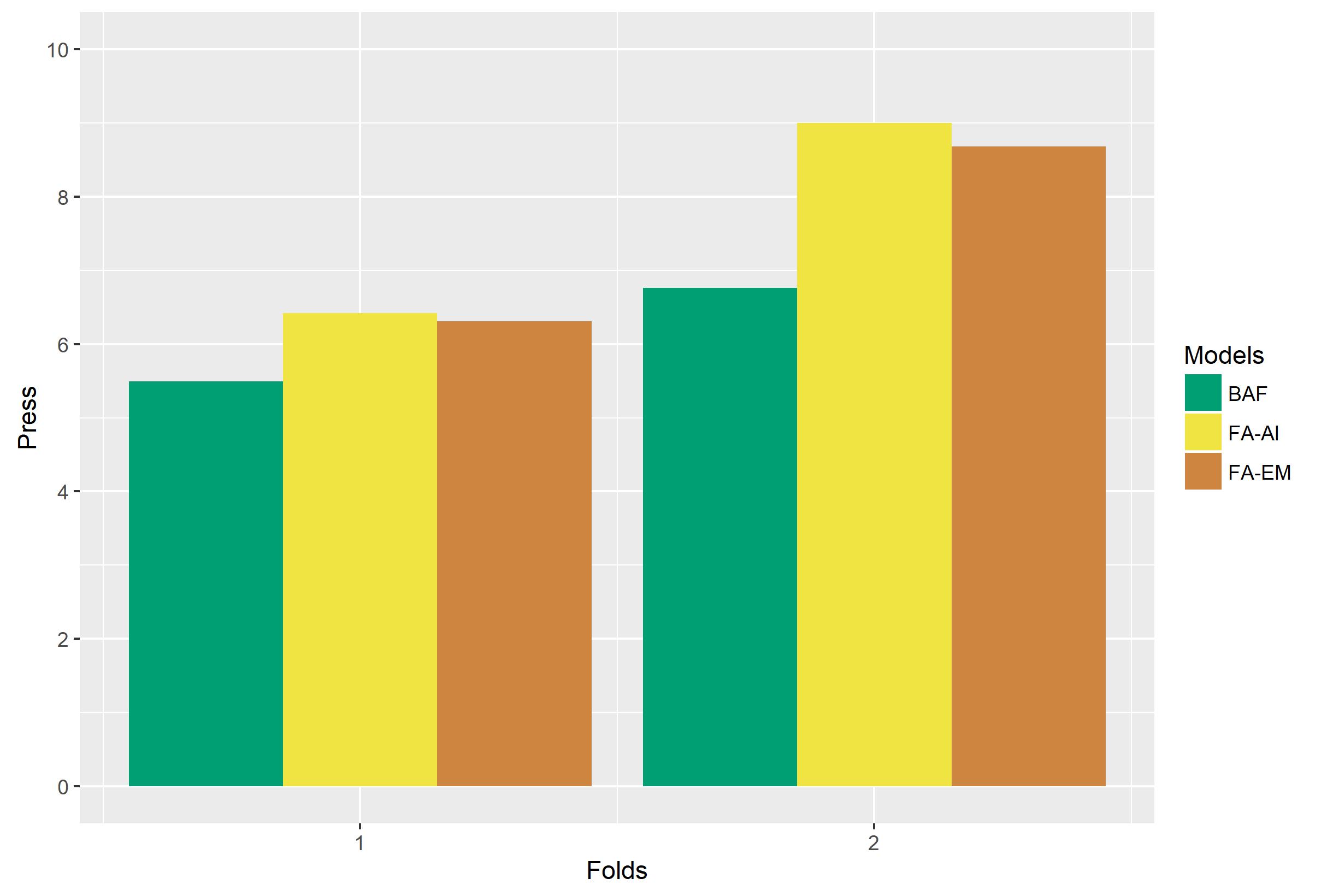

Supplement: S6 Fig — (JPEG) [file pone.0220290.s014.jpeg]
