# Supplementary material for: Bayesian factor analytic model: An approach in multiple environment trials
Source: PLoS One. 2019 Aug 22;14(8):e0220290. doi: 10.1371/journal.pone.0220290 (PMC6705866; doi:10.1371/journal.pone.0220290)
Supplement: S3 Data — (ZIP) [file pone.0220290.s017.zip › BAF/html/00Index.html]

R: Bayesian Factor Analytic

# Bayesian Factor Analytic

---

## Documentation for package ‘BAF’ version 1.0

- DESCRIPTION file.

## Help Pages

|  |  |
| --- | --- |
| BAF-package | Bayesian Factor Analityc (BAF) |
| BAF | Bayesian Factor Analityc (BAF) |
| data\_ge | data\_ge |
| GGE.FA | Bayesian GGE FA |
