# Supplementary material for: Bayesian factor analytic model: An approach in multiple environment trials
Source: PLoS One. 2019 Aug 22;14(8):e0220290. doi: 10.1371/journal.pone.0220290 (PMC6705866; doi:10.1371/journal.pone.0220290)
Supplement: S3 Data — (ZIP) [file pone.0220290.s017.zip › BAF/html/BAF.html]

R: Bayesian Factor Analityc (BAF)

|  |  |
| --- | --- |
| BAF-package {BAF} | R Documentation |

## Bayesian Factor Analityc (BAF)

### Description

Package for multi-environmental data analysis with the Bayesian GGE factorial analytic model.

### Details

|  |  |
| --- | --- |
| Package: | BAF |
| Type: | Package |
| Version: | 1.0 |
| Date: | 2017-11-27 |
| License: | GPL (>= 3) |
|  |
|  |

### Author(s)

Joel Jorge Nuvunga, Carlos Pereira da Silva, Luciano Antonio de Oliveira & Marcio Balestre;

Maintainer: <ccpsilva81@hotmail.com>

---

[Package *BAF* version 1.0 Index]
