# Supplementary material for: Bayesian factor analytic model: An approach in multiple environment trials
Source: PLoS One. 2019 Aug 22;14(8):e0220290. doi: 10.1371/journal.pone.0220290 (PMC6705866; doi:10.1371/journal.pone.0220290)
Supplement: S3 Data — (ZIP) [file pone.0220290.s017.zip › BAF/html/data_ge.html]

R: data\_ge

|  |  |
| --- | --- |
| data\_ge {BAF} | R Documentation |

## data\_ge

### Usage

```
data_ge
```

### Format

A data frame with 300 observations on the following 4 variables.

[,1] `amb:` a numeric vector


[,2] `rep:` a numeric vector


[,3] `gen:` a numeric vector


[,4] `prod:` a numeric vector

### Examples

```
data(data_ge)
## maybe str(data_ge);
```

---

[Package *BAF* version 1.0 Index]
