# Supplementary material for: Bayesian factor analytic model: An approach in multiple environment trials
Source: PLoS One. 2019 Aug 22;14(8):e0220290. doi: 10.1371/journal.pone.0220290 (PMC6705866; doi:10.1371/journal.pone.0220290)
Supplement: S3 Data — (ZIP) [file pone.0220290.s017.zip › BAF/html/GGE.FA.html]

R: Bayesian GGE FA

|  |  |
| --- | --- |
| GGE.FA {BAF} | R Documentation |

## Bayesian GGE FA

### Description

Package for multi-environmental data analysis with the Bayesian GGE factorial analytic model.

### Usage

```
GGE.FA(data = data, niter = 4000, nburni = 1, nthin = 1, n.FA = NULL)
```

### Arguments

|  |  |
| --- | --- |
| `data:` | The data must be organized into a four-columns array and the variables in the following sequence: environment, replication, genotype, and production. The replication variable is a confounding between replicates and environment. |
| `niter:` | Number of iterations of interest for the final MCMC sample. It is a combination between **(niter + nburni)\*nthin**. |
| `nburni:` | Number of jumps that must be performed between the observations to obtain the MCMC sample. |
| `nthin:` | Number of jumps that must be performed between the observations to obtain the MCMC sample. |
| `n.FA:` | Number of terms that are interesting to carry out the analysis of the model. Model family ***(FA1, FA2,..., FAk)*** |

### Details

If the singular vectors converge for two "bimodal" solutions only in signal, see the references below.

To obtain a posteriori means of the singular vectors, see Oliveira et al. (2015)

### Author(s)

Joel Jorge Nuvunga, Carlos Pereira da Silva, Luciano Antonio de Oliveira & Marcio Balestre.

### References

**[1]** Oliveira, L. A., Silva, C. P., Da Silva, Nuvunga, J. J., A. Q., & Balestre, M. (2015). **Credible intervals for scores in the AMMI with random effects for genotype**. Crop Science, 55(2), 465-476.

**[2]** da Silva, C. P., de Oliveira, L. A., Nuvunga, J. J., Pamplona, A. K. A., & Balestre, M. (2015). **A Bayesian shrinkage approach for AMMI models**. PloS one, 10(7), e0131414.

### Examples

```
###------------------------------------------------------------------
### Analysis MCMC

library(msm)            ### package dependency "msm"

data(data_ge)

set.seed(13578913)

obj <- GGE.FA(data = data_ge, niter = 4000, nburni = 1, nthin = 1, n.FA = NULL)

### Objetos MCMC para modelo completo
load("result_GGE_FA.Rdata")

beta <- FAB$beta
R    <- FAB$R
f    <- FAB$f
V    <- FAB$V
L    <- FAB$L

###------------------------------------------------------------------

library(boa)

sample1 <- as.matrix(cbind(beta,f,V,L,R))
colnames(sample1) <- c(paste("VAR",seq(ncol(sample1)),sep=""))

### Raftery and Lewis convergence diagnostics
randl <- boa.randl(sample1, 0.025, 0.005, 0.95, 0.001)
randl
max(randl[,5])


###------------------------------------------------------------------
### Analysis MCMC

library(msm)            ### package dependency "msm"

data(data_ge)

set.seed(13578913)

GGE.FA(data = data_ge, niter = 4000, nburni = 1, nthin = 1, n.FA = 2)

### Objetos MCMC para modelo FAB2
load("result_GGE_FA.Rdata")

beta  <- FAB$beta
R     <- FAB$R
f     <- FAB$f
V     <- FAB$V
L     <- FAB$L
noise <- FAB$noise
delta <- FAB$delta

###------------------------------------------------------------------

library(boa)

sample2 <- as.matrix(cbind(beta,f,V,L,R,delta,noise))
colnames(sample2) <- c(paste("VAR",seq(ncol(sample2)),sep=""))

### Raftery and Lewis convergence diagnostics
randl <- boa.randl(sample2, 0.025, 0.005, 0.95, 0.001)
randl
max(randl[,5])

### plot factorial scores 

n1 <- 20 # number of genotypes

f1 <- apply(f[,1:n1],2,mean)
f2 <- apply(f[,(n1+1):(n1*2)],2,mean)

plot(f1,f2)

### plot factorial loads

n2 <- 5  # number of environments

C1 <- apply(V,2,mean)
C2 <- svd(C1)
v  <- C2$u%*%t(C2$v)
l  <- apply(L,2,mean)
c1 <- v[,1]*l[1]
c2 <- v[,2]*l[2]

names(c1) <- 1:length(l)
plot(c1,c2,pch="*",cex=0.6)
text(c,d, names(c),col='blue',cex=0.85)
```

---

[Package *BAF* version 1.0 Index]
